# Evaluation of variant identification methods for whole genome sequencing data in dairy cattle

**DOI:** 10.1186/1471-2164-15-948

**Published:** 2014-11-01

**Authors:** Christine F Baes, Marlies A Dolezal, James E Koltes, Beat Bapst, Eric Fritz-Waters, Sandra Jansen, Christine Flury, Heidi Signer-Hasler, Christian Stricker, Rohan Fernando, Ruedi Fries, Juerg Moll, Dorian J Garrick, James M Reecy, Birgit Gredler

**Affiliations:** School of Agricultural, Forest and Food Sciences HAFL, Bern University of Applied Sciences, Länggasse 85, CH-3052 Zollikofen, Switzerland; Qualitas AG, Chamerstrasse 56a, CH-6300 Zug, Switzerland; Department VESPAUniversità degli Studi di Milano, 20133 Milan, Italy; University of Veterinary Medicine Vienna, Veterinärplatz 1, A-1210 Vienna, Austria; Department of Animal Science, Iowa State University, 1221 Kildee Hall, 50011-3150 Ames, IA USA; Technische Universität München, Liesel-Beckmann-Str. 1, D-85354 Freising, Germany; agn Genetics GmbH, 8b Börtjistrasse, CH-7260 Davos, Switzerland

**Keywords:** Next-generation sequencing analysis, Single nucleotide variant identification, Pipeline

## Abstract

**Background:**

Advances in human genomics have allowed unprecedented productivity in terms of algorithms, software, and literature available for translating raw next-generation sequence data into high-quality information. The challenges of variant identification in organisms with lower quality reference genomes are less well documented. We explored the consequences of commonly recommended preparatory steps and the effects of single and multi sample variant identification methods using four publicly available software applications (Platypus, HaplotypeCaller, Samtools and UnifiedGenotyper) on whole genome sequence data of 65 key ancestors of Swiss dairy cattle populations. Accuracy of calling next-generation sequence variants was assessed by comparison to the same loci from medium and high-density single nucleotide variant (SNV) arrays.

**Results:**

The total number of SNVs identified varied by software and method, with single (multi) sample results ranging from 17.7 to 22.0 (16.9 to 22.0) million variants. Computing time varied considerably between software. Preparatory realignment of insertions and deletions and subsequent base quality score recalibration had only minor effects on the number and quality of SNVs identified by different software, but increased computing time considerably. Average concordance for single (multi) sample results with high-density chip data was 58.3% (87.0%) and average genotype concordance in correctly identified SNVs was 99.2% (99.2%) across software. The average quality of SNVs identified, measured as the ratio of transitions to transversions, was higher using single sample methods than multi sample methods. A consensus approach using results of different software generally provided the highest variant quality in terms of transition/transversion ratio.

**Conclusions:**

Our findings serve as a reference for variant identification pipeline development in non-human organisms and help assess the implication of preparatory steps in next-generation sequencing pipelines for organisms with incomplete reference genomes (pipeline code is included). Benchmarking this information should prove particularly useful in processing next-generation sequencing data for use in genome-wide association studies and genomic selection.

**Electronic supplementary material:**

The online version of this article (doi:10.1186/1471-2164-15-948) contains supplementary material, which is available to authorized users.

## Background

Practical application of genomic technologies, such as large-scale use of single nucleotide variant (SNV) arrays in animal and plant breeding, has become routine in many areas of the life sciences. Taking both polygenic additive (pedigree) effects and genomic (SNV) effects into account, between 71 and 85% of the genetic variance observed in phenotypic traits of interest in cattle can be explained solely by SNV effects [1] and the number of genotyped animals in cattle populations worldwide is increasing steadily [2]. Fueled by decreasing costs, advances in next-generation sequencing (NGS) technologies enable identification of more complex forms of genetic variation (e.g. short insertions and deletions (InDels), copy number variations (CNVs), etc.). These advances will inevitably foster our ability to partition the genetic variance underlying traits of interest. While some applications of NGS require *de novo* sequencing of an individual organism (sample), re-sequencing may also be possible if a reference genome for the species of interest is available.

The translation of raw NGS reads into tangible variants (SNVs, InDels, CNVs, etc.) via re-sequencing is a specific, delicate and computationally demanding task [3] and comprises three steps. First, short reads of DNA are aligned to an existing reference genome (referred to as alignment). Second, sequence differences between the sample being sequenced and the reference genome are identified (referred to as variant calling [4]). A myriad of alignment (see [5] for a review) and variant identification software programmes are available (e.g. the UnifiedGenotyper (UG) or the HaplotypeCaller (HC) of the genome analysis toolkit (GATK) [6, 7]; Platypus (PL) [8]; SAMtools (SAM) [9]; etc.), the majority of which can be obtained free of charge. As a final step, variants are screened and filtered to remove potential false positives common in most NGS technologies (see [10]).

As sequencing costs decline, reference genomes are becoming available for an increasing number of organisms, including agriculturally important species such as cattle (see [11] for a review). The *Bos taurus* reference genome UMD3.1 is similar in size to the human genome and contains ~2.8 billion base pairs, approximately 10% of which are not assigned to any chromosome [12]. The N50 size can be used to compare the quality of genome assemblies of similar size: it represents contig size such that 50% of the genome is contained in contigs of length *N* or greater [12]. Because the N50 size for the UMD3.1 reference genome (UMD3.1 accession number GCA_000003055.3: N50 = 96,955b) is much shorter than that of the current human reference (GRCh38 accession number GCA_000001405.15: N50 = 56,413,054b), the UMD3.1 reference genome will not likely allow the same accuracy in alignment, variant identification and further downstream analysis as the human reference allows. Nevertheless, algorithms and software developed for alignment and variant identification of human NGS data provide an excellent resource for translating NGS data of other non-human organisms, such as cattle, into genetic variants for application in genome wide association studies and genomic selection programmes.

Several approaches to variant identification are possible. The simplest variant detection methods identify variants on a per-sample basis, one position at a time. Once a variant locus is found, the most likely genotype for that locus is determined stochastically based on a consensus of aligned reads. If multiple samples are analyzed simultaneously, an *a priori* likelihood of finding a variant locus given the observed data is derived, and the most likely genotype at a given position is determined. Either single or multi sample variant identification methods can be implemented in the UG [6, 7]; and SAM [9]. More advanced haplotype-based methods incorporate the correlation between adjacent variants within the variant detection procedure. Such methods use linkage disequilibrium between nearby variants to further enrich variant identification. Haplotype-based methods are implemented in PL [8] and the GATK HC [6, 7]. The haplotype-based variant detection approach can also be conducted in either single or multi sample settings.

Read chimerism, base pair tautomerisms and signal intensity issues can contribute to false positive variant detection by causing stochastic inaccuracies, general sequencing errors, and misalignments in NGS data [7, 10]. Aside from the variant identification approach itself, a number of optional auxiliary steps have been recommended to improve the quality of NGS-derived variants. These steps are conducted before (preparatory steps) or after variant identification (filtering). The first generally recommended preparatory step is the identification of falsely duplicated reads (mainly artifacts caused by PCR), which reduces bias in variant detection [13]. Secondly, local realignment around single or multiple bases that are either missing in the reference (insertion) or missing in the DNA sequence being analyzed (deletion) is also commonly recommended [7]. Realignment cleans up spurious SNVs that result from misalignment of reads around known alignment gaps and helps detect false negative SNVs in the near vicinity of InDels (insertions and deletions). Furthermore, the full alignment context is used to determine whether the reported divergence from the reference (i.e. the insertion or deletion) actually exists [14]. Finally, flow cell lane, machine cycle (base position within the read), sequencing context (preceding and current nucleotide) or other technical aspects may influence base quality scores, which help characterize the quality of the bases in the individual reads. Base quality score recalibration is recommended to lower the number of falsely identified SNVs and to lower false confidence in identified bases [14]. After variant identification, further filters can be implemented according to the individual dataset under consideration.

Variant detection methods can be evaluated using genome simulations based on reference genomes [15]. Given that all mammalian reference genomes are incomplete and whole-genome alignment is imperfect, simulations may not provide realistic results, a difficulty that has prompted the use of real data [16]. Another common evaluation method in human studies is to compare variants from NGS data to those of array genotyping (e.g. [16–18]), to results from the 1000 genomes project [19], or to other existing resources of human sequence (i.e. [20]). Resources for cattle are not as comprehensive, however low, medium and high density SNV arrays are available (e.g. Illumina BovineHD BeadChip® or Affymetrix Axiom® Genome-Wide BOS 1 Array), thus allowing an estimation of relative accuracy between software (Table 1).

**Table 1:** **Variant genotypes compared for concordance between the array-based and sequence based methods to determine concordance, sensitivity and discrepancy between the two assays (a) and measures of concordance (b)**

a) **NGS-based genotypes**			**Array-based genotypes (gold standard)**		
			Homozygous reference	Heterozygous	Homozygous alternative
			**AA**	**AB**	**BB**
	Homozygous reference	**AA**	*a*	*b*	*c*
	Heterozygous	**AB**	*d*	*e*	*f*
	Homozygous alternative	**BB**	*g*	*h*	*i*
	Genotype not identified	**–**	*k*	*l*	*m*
b) **Measures of concordance**	SNP concordance				
	Genotype concordance				
	Non-reference sensitivity				
	Non-reference discrepancy				

Array-based information from the Illumina BovineHD BeadChip® (BovineSNP50 v1 DNA Analysis BeadChip® not shown) was considered the “gold-standard” and compared to next-generation sequencing-based variants obtained using a Illumina HiSeq2000 platform with various variant identification software, where genotypes are identified as:

a = homozygous reference in both NGS-based data and array-based data.

b = homozygous reference in NGS-based data, but as heterozygous in array-based data.

c = homozygous reference in NGS-based data, but as homozygous alternative in array-based data.

d = heterozygous in NGS-based data, but as homozygous reference in array-based data.

e = heterozygous in both NGS-based data and array-based data.

f = heterozygous in NGS-based data, but as homozygous alternative in array-based data.

g = homozygous reference in NGS-based data, but as homozygous reference in array-based data.

h = heterozygous in NGS-based data and array-based data, but as heterozygous in array-based data.

i = homozygous alternative in both NGS-based data and array-based data.

k = not found in NGS-based data, but as homozygous reference in array-based data.

l = not found in NGS-based data and array-based data, but as heterozygous in array-based data.

m = not found in NGS-based data, but as homozygous alternative in array-based data.

*(Table adapted from DePristo et al.,* [7] *and Jansen et al.,* [30] *).*

Aside from measuring concordance between NGS data and array data, the ratio of transitions (pyrimidine-pyrimidine or purine-purine mutations) to transversions (pyrimidine-purine or purine-pyrimidine mutations; Ti/Tv ratio) can be used as a convenient diagnostic to measure the quality of NGS data (e.g. [7, 21]). The genome-wide Ti/Tv ratio is reported between 2.0 and 2.2 in human whole-genome sequence data [7, 21], whereby this ratio is higher in exomes due to the increased presence of methelated cytosine in CpG dinucleotides in exonic regions [22]. Because this bias in favour of mutations between bases with similar biochemical properties (transitions) over those with dissimilar properties (transversions) is dependent on both CpG and GC content of the region, the Ti/Tv ratio is a useful diagnostic to measure quality across the genome [14].

The objective of this study was to investigate which methods and software work best for detection of high quality genetic variants using NGS data in cattle, with a specific focus on single nucleotide variants. Using whole-genome sequence information from 65 individuals, we a) explore the implications of preparatory steps commonly recommended in human analysis, b) compare results of single and multi sample variant detection achieved using four publicly available variant detection software programmes, c) provide a comparison of computational processing time, and d) compare accuracy and completeness of SNVs identified in NGS data by comparing them to genotypes from the same individuals generated with either high- or medium-density SNV arrays, as well as to analyse genome-wide Ti/Tv ratios. Through benchmarking different variant detection methods in cattle, preliminary recommendations for variant identification in other organisms can be extrapolated. Our findings can serve as a reference for choosing variant identification software and can help assess the implication of preparatory steps in NGS pipelines for species with lower-quality or unfinished reference genomes.

## Results and Discussion

### Alignment and coverage

Approximately 24 billion paired-end reads were obtained for the 65 sequenced animals. An average of 96.8% of these reads (range 86.6% - 98.2%) were mapped to 30 chromosomes (autosomes 1 – 29, X) of the bovine reference genome assembly UMD3.1 [11]. Approximately 1.7 billion (PCR and optical) duplicate reads (average 7.3%, range 4.2% - 10.6%) were marked and excluded from further analysis. Average coverage was 12.1 reads per base; average coverage per-animal ranged from 10.1 - 17.5. See Additional file 1: Table S1 for individual alignment and coverage information.

### Single sample variant detection

**Figure 1:**
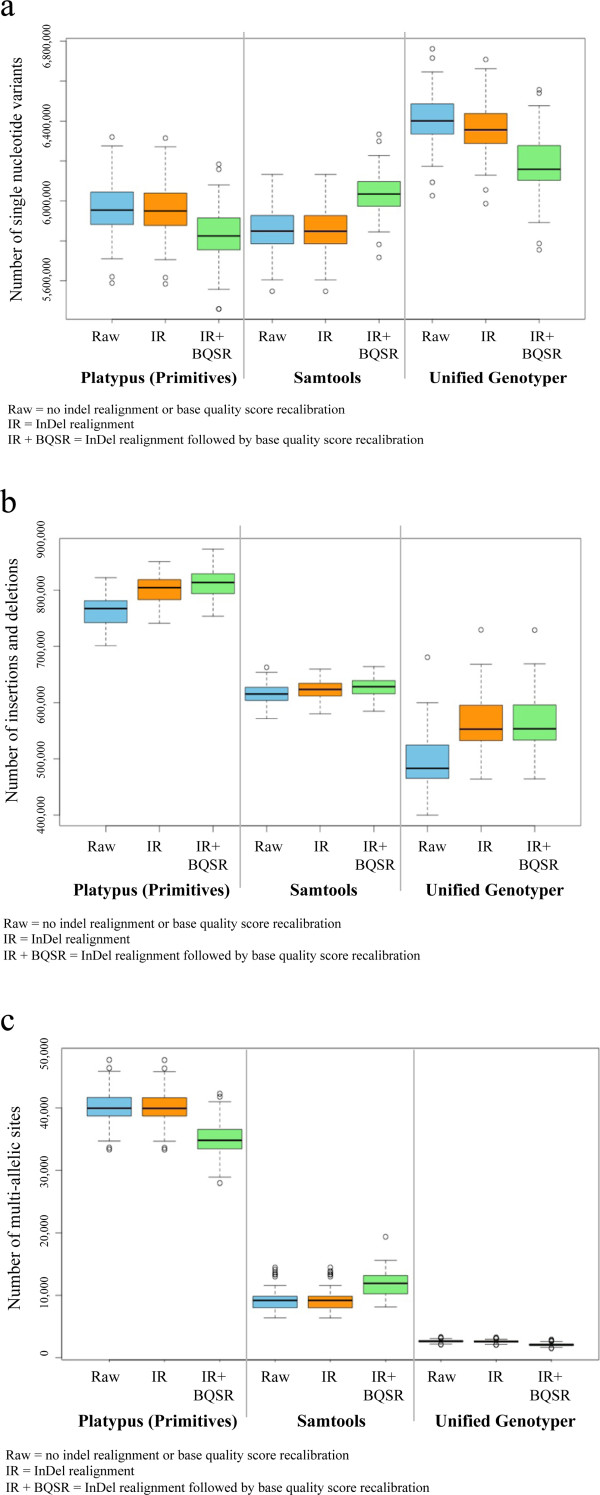
**Distributions of single nucleotide variant counts (a), insertion and deletion counts (b), and multi-allelic site counts (c) identified per animal.** For Platypus results, multi-nucleotide variants were split into allelic primitives for fair comparison between software. Single nucleotide variant counts **(a)**, insertion and deletion counts **(b)**, and multi-allelic site counts **(c)** identified per animal (n = 65; BTA1-29, BTAX) using single sample variant detection with Platypus, Samtools, and the UnifiedGenotyper following three pre-calling approaches.

The results of single sample variant detection are in Figure 1. Depending on software and preparatory steps, an average of 5,854,886 - 6,404,094 SNVs (Figure 1a), 496,203 – 832,689 InDels (Figure 1b) and 2,098 – 40,462 multi-allelic sites (i.e. sites with more than two alleles, Figure 1c) were identified per animal (n = 65). Detailed information on the sequencing process (variant counts per individual, etc.) is given in Additional file 2: Table S2. UG identified the most SNVs, followed by PL and SAM. Because PL identifies multi-nucleotide variants, PL originally had the lowest average number of SNV per sample (4,873,149) because some variants were “hidden” in multi-SNV replacements. After splitting multi-nucleotide variants into their allelic primitives (hereafter referred to as PL_PRIM), a fair comparison was achieved and the average per-sample number of variants identified with PL_PRIM increased to 5,826,468. When single-sample results of all 65 animals were combined, 20,647,891 - 21,984,283 unique SNV were identified (Table 2).

**Table 2:** **Total number of single nucleotide variants (SNVs), insertions and deletions (InDels), and Transition/Transversion Ratios found using single and multi sample calling methods with HaplotypeCaller (HC), Platypus (PL), Platypus results after multi-nucleotide variants were split into allelic primitives (PL_PRIM), Samtools (SAM), and the UnifiedGenotyper (UG) for 65 animals**

Calling method	Total number of SNVs		Total number of InDels		Transition/Transversion ratio	
	***Single sample calling, combined***	***Multi sample calling***	***Single sample calling, combined***	***Multi sample calling***	***Single sample calling, combined***	***Multi sample calling***
HC	-	19,901,885	-	2,685,032	-	2.138
PL	17,709,672	16,894,054	2,973,025	2,890,066	2.178	2.165
PL_PRIM	20,869,015	19,759,134	2,864,147	2,890,412	2.105	2.058
SAM	20,647,891	18,767,273	2,682,094	1,997,791	2.176	2.240
UG	21,984,283	22,048,382	2,485,677	2,741,468	2.024	1.974

The combined results of all 65 single samples represent single sample calling results.

### InDel realignment

**Figure 2:**
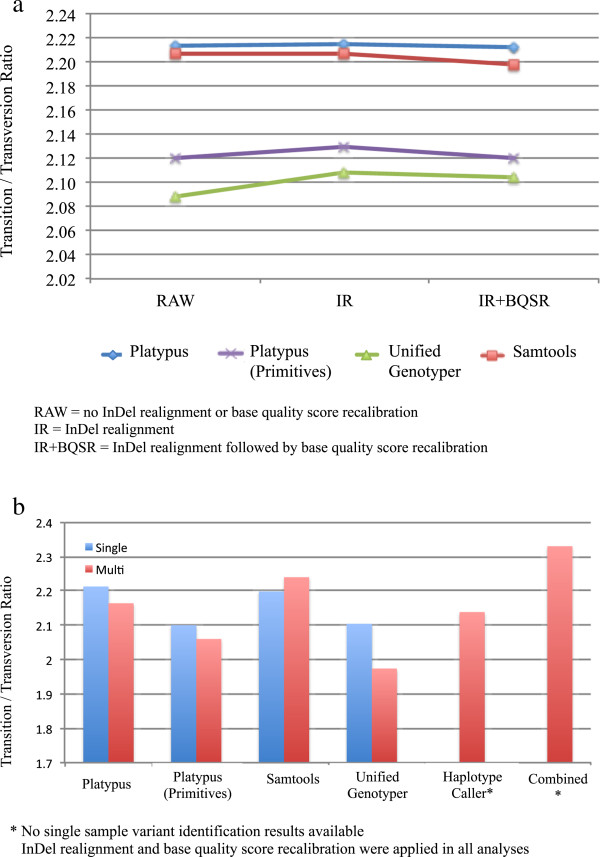
**Average transition/transversion ratios over all animals using single sample variant identification (a) and transition/transversion ratios for variant identification with single and multi sample detection methods, as well as combined over all multi sample detection methods (b).** Average transition/transversion ratios for variant identification with single sample detection methods using Platypus, Platypus Primitives, Samtools, UnifiedGenotyper and HaplotypeCaller (n = 65 samples, BTA1-29) are shown in **(a)**. Transition / transversion ratios for variant identification with single and multi sample detection methods using Platypus, Platypus Primitives, Samtools, UnifiedGenotyper and HaplotypeCaller (n = 65 samples, BTA1-29) and a consensus data set (variants called by Platypus Primitives + Samtools + UnifiedGenotyper + HaplotypeCaller) are shown in **(b)**.

InDel realignment had a slight effect on the number of SNVs and InDels identified, with the largest effects observed in UG. InDel realignment reduced the number of SNVs identified (PL: -4,548; SAM: -1,518; UG: -47,338), increased the number of InDels detected (PL: +37,194; SAM: +8,517; UG: +68,758), and decreased the number of multi-allelic sites identified (PL: -32; SAM: -7; UG: -53) (Figure 1a, b, c) in all samples. InDel realignment did not heavily reduce the number of SNVs identified by SAM compared to PL or UG, indicating that SAM effectively removed false positives prior to IR with GATK (only 1,518 less SNVs were identified with SAM when InDel realignment was done prior to variant detection). InDel realignment resulted in less SNVs being identified, because spurious SNVs caused by incorrect alignments in the close vicinity of real InDels were no longer detected as variants. During InDel realignment, the RealignerTargetCreator of the GATK creates a list of regions in which InDels are likely to occur depending on a set of known InDels and SNVs. Following this initial identification step, local realignment of reads spanning the InDel occurs [14]. However, if the InDel is incorrect in the primary alignment (possibly due to chimeric read fragments, structural variations and/or misassemblies due to a poor-quality reference genome), InDel realignment may incorrectly realign the read segments surrounding the InDel. InDel realignment therefore relies on a trustworthy set of known InDels and SNVs, which may not yet be available for all species.Figure 2a shows the average Ti/Tv ratios of RAW, IR and IR + BQSR for single sample results of PL, SAM, UG and PL after multi-nucleotide variants were split into their allelic primitives (PL_PRIM). The Ti/Tv ratios are in the same range as those in human data. The benefit of InDel realignment is most apparent in UG, where an increase in Ti/Tv ratio can be observed compared to RAW. For PL and SAM the Ti/Tv ratio remained virtually unchanged after realignment. Splitting multi-nucleotide variants identified by PL into allelic primitives lowered the average Ti/Tv ratio, however a slight improvement after InDel realignment was observed. The use of InDel realignment is therefore recommended for variant identification with UG and PL_PRIM, however SAM and PL do not benefit markedly from additional realignment with GATK.

### Base quality score recalibration

Base quality score recalibration reduced the average number of SNVs identified in PL (-127,292) and UG (-181,119), but not in SAM (+182,943) (Figure 1a). The number of SNVs identified after InDel realignment and base quality score recalibration is expected to decrease, as quality scores are initially overestimated and more SNVs fall below the cut-off after recalibration. By lowering base quality scores through recalibration, confidence in weak variants should decrease and, as a result, the number of false positives is expected to drop [14]. The observed increase in SNVs identified with SAM after base quality score recalibration likely corresponds with the effective removal of false positive SNVs by SAM prior to IR and BQSR and the less stringent default quality scores in SAM, though the increase is minimal. Recalibration of base quality scores slightly increased the number of InDels for PL and SAM (no change in UG, Figure 1b).The effect of IR + BQSR on the number of SNVs identified was more obvious than the effect of IR alone for PL and SAM. Depending on variant detection software used, the total average number of SNVs identified per animal increased (SAM) or decreased (PL and UG) by around 3.0 – 4.0%. In contrast, the number of InDels identified after IR + BQSR did not change markedly compared to the number of InDels identified after IR alone. The Ti/Tv ratio decreased slightly for PL_PRIM, SAM and UG (no change in PL, Figure 2a) after BQSR, indicating that BQSR using default settings and the current available resources may actually decrease overall variant quality for these variant identification methods.

Liu et al. [17] analysed the effect of preparatory steps in whole exome sequencing data and found no clear effect of InDel realignment or base quality score recalibration in five whole exome sequencing samples of breast cancer patients. The authors state that the relative contribution of each preparatory step to the accuracy of variant identification is dependent on read depth; the lack of a sufficient number of reads in a low-coverage scenario limits the power of local multiple sequence alignment. In contrast, local realignment can benefit from consistent alignment among reads when coverage is high, thus effectively reducing the number of false positive SNVs. Li [16] focused on deep Illumina sequencing data from two human cell lines (55–100 fold coverage) and found “only” a 0.1% difference in the number of variants before and after InDel realignment and base quality score recalibration using both SAM and GATK. Although the authors regarded this difference as negligible considering the increased computational costs, a 0.1% difference in the number of variants may represent a good proportion of false positives being eliminated. Unfortunately, the resulting variants were not further analysed for quality (e.g. Ti/Tv ratio) leaving the key question regarding the effect of InDel realignment or base quality score recalibration unanswered.

Both InDel realignment and base quality score recalibration rely on a reference set of high-quality known InDels and SNVs. Existing resources for human sequence (e.g. HapMap [20], the Omni family of arrays from Illumina [23] or results from the 1000 genomes project [19]) provide qualitatively solid references, whereas the quality of bovine resources such as dbSNP [24] is notably lower. Although there are currently close to 70 million bovine SNVs included in dbSNP, only very few of them are validated (i.e. at least one clustered SNV determined using a non-computational technique or both population frequency data and genotype data included in the entry). In contrast, approximately 28 million human SNV are validated. High-density array information, such as that from the Illumina (BovineHD BeadChip®) or Affymetrix (Axiom® Genome-Wide BOS 1 Array), provides higher-quality information, but for only a limited number of SNVs (there are a total of 908,866 mapped SNVs on these two chips combined). As the number and quality of known InDels and SNVs in the bovine genome increase and reference information improves, we can expect better and more dependable effects of InDel realignment and base quality score recalibration in variant identification pipelines.

### Multi sample variant detection

The differences between single and multi sample variant identification methods were generally slight in terms of number of SNVs and Ti/Tv ratios, and depended on software. Results of multi sample variant detection (conducted after InDel realignment and base quality score recalibration) are shown in Table 2. Variant detection with UG (multi sample) resulted in the highest number of variants (22,048,382), followed by HC (19,901,885) and SAM (18,767,273). As observed in single sample variant identification, PL originally had the lowest number of variants (16,894,054), because some variants were “hidden” in multi-SNV replacements as described previously. After splitting multi-nucleotide variants into their allelic primitives, the number of variants identified with PL increased with PL_PRIM to 19,759,134. Le Roex et al. [25] compared the number of SNVs identified with SAM and GATK in African buffalo and identified considerably more SNVs with GATK than with SAM using multi sample variant identification methods. Though not as pronounced, this agrees with both our single sample and multi sample results.

The total number of SNVs identified by combining single sample results of all 65 animals was higher for PL and SAM than when multi sample variant identification was carried out on all 65 animals simultaneously (Table 2). This was not the case for the UG, although the difference was very slight. Similarly, Liu et al. [26] found that the UG multi sample pipeline resulted in 16.6% more raw SNVs than single sample results, although they found no difference in the number of SNVs found in single and multi sample SAM pipelines.In terms of variant quality, Ti/Tv ratios for single sample calling with PL, PL_PRIM and UG were higher than those observed in multi sample calling (Figure 2b), whereby the Ti/Tv ratio for single sample calling with SAM was lower than that for multi sample calling (no single sample results available for HC). This difference was most prominent in UG, in which the Ti/Tv ratio dropped by approximately 6% when multi sample calling was applied.

Surprisingly, the total number of InDels identified using single sample variant detection methods was also higher for PL (+4%) and SAM (+34%) than when multi sample methods were used. Again, this was not the case for the UG (-10%). In contrast, Liu et al. [26] analysed human data and found that multi sample analysis increased the number of InDels identified considerably (SAM: +88.2%, UG: +92.6%) compared to single sample variant detection. It is possible that the number of InDels is inherently lower in cattle populations because their effective population size is much smaller than it is in humans, however a more likely reason for this discrepancy could also be the quality of the reference genome. The percentage of variants identified as InDels in the human genome has been estimated at up to 18% [27] whereas the number of InDels in cattle has been estimated at only 5.65% [28] of the total variants identified, although this may also be only a difference in reference quality. Depending on software and methods used, we found 7.19 – 12.25% of the variation observed was due to InDels.

**Figure 3:**
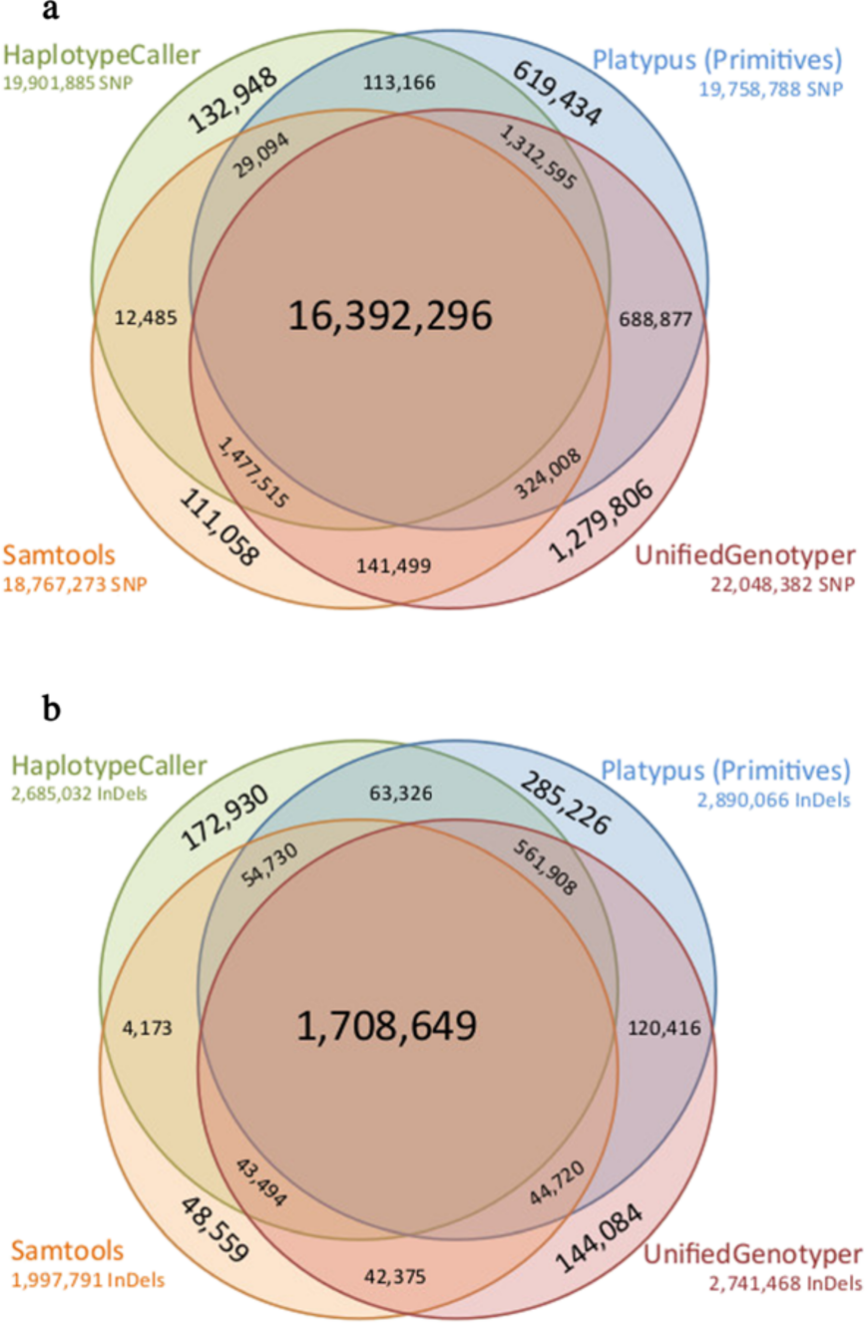
**Consensus single nucleotide variants (a) and insertions and deletions (b) identified using multi sample variant detection methods.** Consensus single nucleotide variants **(a)** and insertions and deletions **(b)** identified from whole genome sequencing data using four multi sample variant detection methods (Platypus Primitives, Samtools, UnifiedGenotyper and HaplotypeCaller).

Consensus SNVs and InDels for multi sample results may provide a simple method to ensure higher variant accuracy, although this approach is computationally intensive. Analysis of variant sets produced in phase one of the human 1000 genomes project [19] showed that a consensus approach to identifying variants led to a higher quality data set; variants identified in all software applications were more accurate than those identified in any single variant set [19]. Li [16] also recommended taking the intersection of raw variants from independent variant identification methods and applying a software-oblivious filter to derive a final variant set. We used the vcf-intersect command of VCFTools [29] to count consensus SNVs and InDels (Figure 3). In this study, 16.4 million SNV positions (unfiltered) were found by all four multi sample variant detection methods (Figure 3a), and 1.7 million InDels were found by all four multi sample variant detection methods (Figure 3b). A consensus vcf file was created using the CombineVariants walker of the GATK [6, 7] in which only SNV found in all multi sample vcf files (HC, PL_PRIM, SAM and UG) were included. The Ti/Tv ratio in the consensus vcf was higher than the Ti/Tv ratios in the individual single and multi sample variant sets (Figure 2b), indicating higher variant quality in the consensus variant set. This approach is, however, extremally computationally intensive, as variant identification must be conducted using multiple software applications and then combined. An alternative could be to use only two variant sets (i.e. SAM + PL_PRIM), which also results in improved Ti/Tv ratios (Ti/Tv =2.30), but is computationally less intensive than including all possible variant sets.

By using default parameters, we did not fine-tune all possible options available in the individual software applications. Nevertheless, using default settings in both single and multi sample variant identification yielded good performance while maintaining output quality. Our goal was to provide an initial overview of methods using the default settings recommended; it should be noted that each dataset must be treated uniquely and alternative parameter settings may deliver more accurate results.

If possible, we recommend a consensus approach for variant identification using SNVs identified by all software, which resulted in the highest SNV quality and should be considered the “golden standard” for variant identification in organisms with lower-quality reference genomes. If computational constraints do not allow a consensus approach to variant identification, the tradeoff between quality and quantity of SNVs must be faced (computation time is discussed in the next section). The UG identified the highest number of SNV in both single and multi sample methods, however the number of false positive SNVs was also highest. SNVs identified with PL had the highest quality of single sample methods, however the number of SNV identified by PL may appear to be low if “hidden” SNVs are not split into allelic primitives. SAM identified a good number of SNVs, which were of comparable quality to those identified by PL. Both PL and SAM are likely a good choice of software for organisms with lower-quality reference genomes, as the built-in InDel realignment algorithm seems to efficiently remove false positives, making the use of lower quality resources (i.e. lower-quality bovine dbSNP information) superfluous.

### Computation time

**Figure 4:**
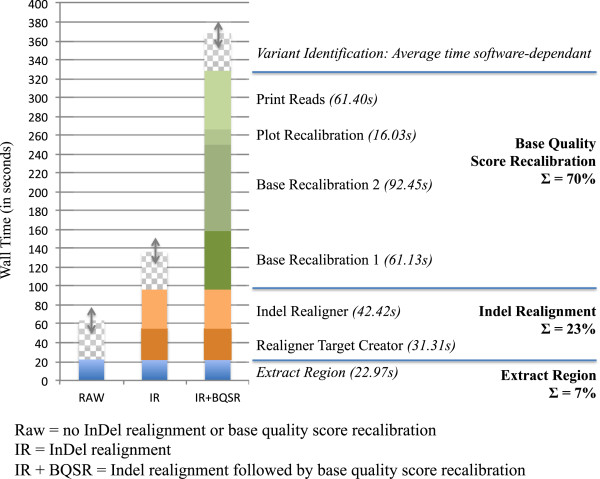
**Average per-sample wall clock computation time required for common preparatory steps InDel realignment and base quality score recalibration (n = 65 samples, chromosomal region 5 Mb in length).**

**Figure 5:**
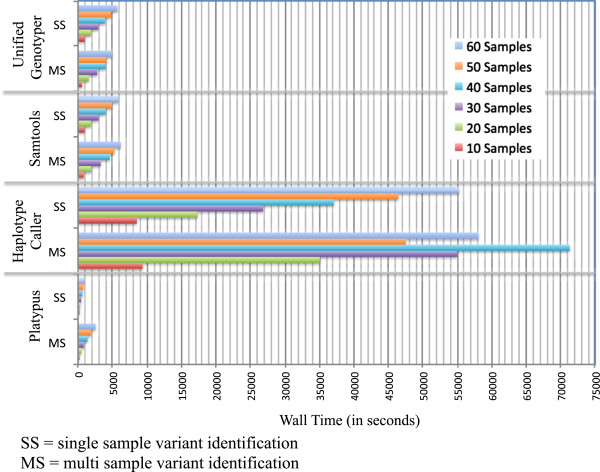
**Wall clock computation time required for variant identification using Platypus, HaplotypeCaller, Samtools and UnifiedGenotyper on a chromosomal region 5 Mb in length with single (SS) or multi (MS) sample variant identification methods and varying numbers of samples (10, 20, 30 40, 50, 60).**

**Figure 6:**
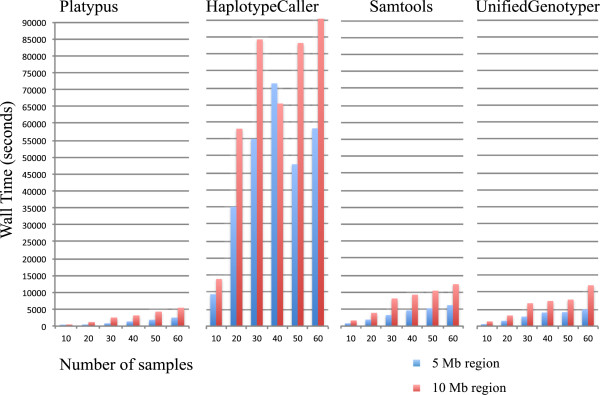
**Average wall clock computation time required for multi sample variant identification with varying numbers of samples (10, 20, 30, 40, 50, 60) and different lengths of chromosomal regions (5 Mb and 10 Mb) using different software (Platypus, HaplotypeCaller, Samtools and UnifiedGenotyper).**

Average computation time required for IR and IR + BQSR is depicted in Figure 4; InDel realignment was considerably faster than base quality score recalibration. The time required for single sample variant detection varied considerably between software applications, whereby PL was fastest, followed by SAM and UG, and HC was slowest. To compare time required for single and multi sample variant detection, the time required for several single sample runs was summed and compared to multi sample runs for the same number of samples (Figure 5). Surprisingly, the difference in runtime between single and multi sample methods for the same number of samples was negligible with the exception of PL, in which a clear speed advantage of single over multi sample variant detection was observed. As expected, increasing the length of the chromosomal region and the number of samples analysed also linearly increased computation time (Figure 6), except for MS HC. This may have been caused by limited resources, which can cause unexpected behaviour. For PL, SAM and UG pipelines including InDel Realignment and base quality score recalibration, more than half of the computing time was for preparatory steps. In pipelines involving HC, which was markedly slower than the other software, variant identification required more than double the amount of time necessary for preparatory steps.

InDel realignment and base quality score recalibration had only slight effects on the number of SNVs, InDels and multi-allelic sites identified. The effect of InDel realignment on Ti/Tv ratio was only positive for UG, and the effect of base quality score recalibration on Ti/Tv ratio was negligible (PL) or even slightly negative (SAM, UG). Given that computational costs in terms of time are very high, we recommend InDel realignment only in combination with UG. The use of BQSR for organisms with lower-quality resource information seems superfluous until better resources become available.

### Concordance

Concordance, measured as non-reference sensitivity (NRS), non-reference discrepancy (NRD), SNV concordance and genotype concordance, was calculated by comparing variants identified in NGS data to array information from the Illumina BovineSNP50 v1 DNA Analysis BeadChip® (n = 17 samples) and the Illumina BovineHD BeadChip® (n = 48 samples; Table 1). Detailed results of NGS concordance with the Illumina BovineSNP50 v1 DNA Analysis BeadChip® are shown in Additional file 3: Table S3; results of NGS concordance with the Illumina BovineHD BeadChip® are given in Additional file 4: Table S4. In this section we discuss concordance results with the high-density array (medium-density array results mirrored those of the high-density analysis and are not discussed in detail).

**Figure 7:**
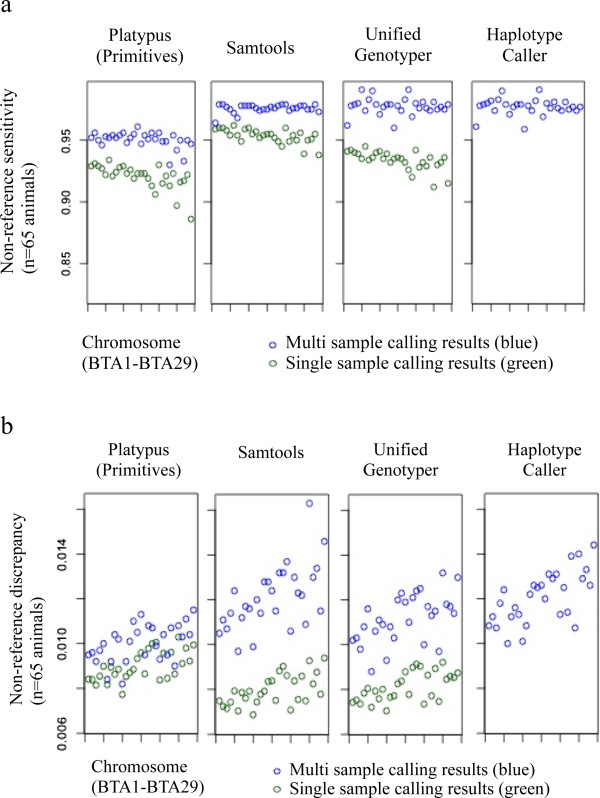
**Non-reference sensitivity (a) and non-reference discrepancy (b) for single nucleotide variants identified using Platypus Primitives, Samtools, UnifiedGenotyper and Haplotype Caller (single vs. multi sample variant identification) using variants identified with the Illumina BovineHD BeadChip® as a gold standard.** Indel realignment and base quality score recalibration were conducted for both single and multi sample calling results.

An NRS of unity represents perfect concordance between the NGS variant set and the array. Jansen et al. [30] compared NRS and NRD of array-derived genotypes with sequence-derived genotypes of 43 Fleckvieh animals for BTA1 using SAM and found that low coverage (<7x) had a negative effect on both of these parameters. In our study, the NRS was generally higher in multi sample methods than in single sample methods for all software (Figure 7a), however this effect was most pronounced in PL and least pronounced in SAM. Our NRS results for SAM are similar to those of Jansen et al. [30]. Liu et al. [26] compared sensitivity of single and multi sample methods using whole exome sequence data of 20 individuals and observed only a slight improvement in sensitivity when multi sample methods were applied, with the exception of SAM, in which a considerable drop (30%) in sensitivity was observed. For UG, Liu et al. [26] observed an increase in sensitivity of around 1%, whereas our results showed a slightly more pronounced improvement of NRS when multi sample methods were applied (4%). Surprisingly, Cheng et al. [18] found slightly better sensitivity in single sample results of SAM and UG compared to multi sample results in a population-based sample of 96 Southeast Asian Malays with deep whole genome sequence information.

**Figure 8:**
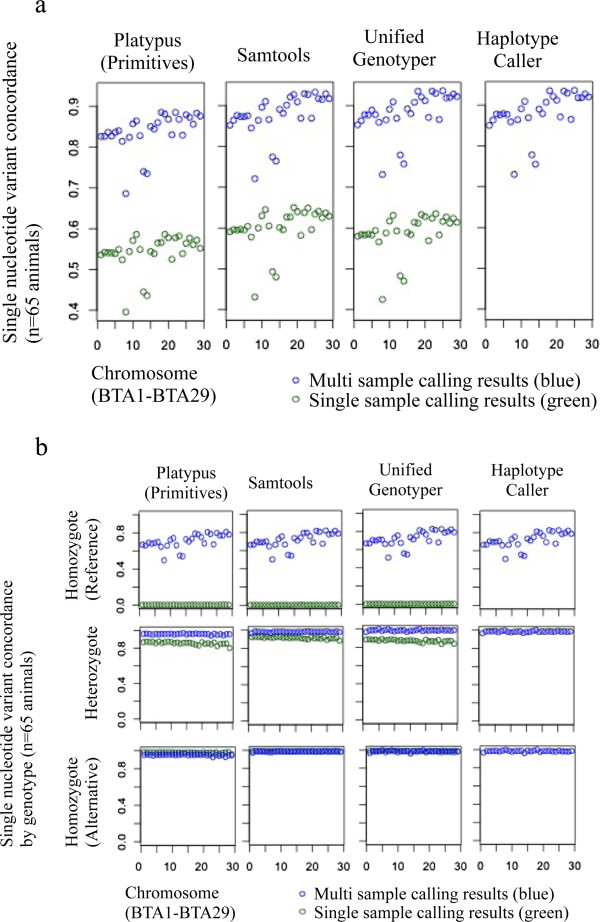
**Single nucleotide variant concordance (a) and single nucleotide variant concordance by array genotype (b) with variants identified using Platypus Primitives, Samtools, UnifiedGenotyper and Haplotype Caller (single vs. multi sample variant identification) and variants identified with the Illumina BovineHD BeadChip® as a gold standard.** Indel realignment and base quality score recalibration were conducted for both single and multi sample calling results.

**Figure 9:**
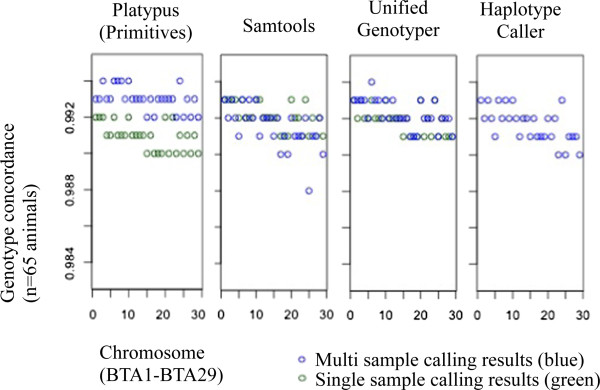
**Genotype concordance between genotypes identified using Platypus Primitives, Samtools, UnifiedGenotyper and HaplotypeCaller (single vs. multi sample variant identification) and genotypes identified with the Illumina BovineHD BeadChip® as a gold standard.** Indel realignment and base quality score recalibration were conducted for both single and multi sample calling results.

NRD results for SAM are slightly lower than those of Jansen et al. [30], which can be explained by the slightly higher coverage in our study. The NRD is a measure of false positives; its importance depends on the purpose of the study (less high quality variants vs. more variants which may be of lesser quality). Generally, ratios should be as close to zero as possible. Figure 7b shows that single sample variant identification resulted in marginally lower NRD values than those observed using multi sample methods. Though slight, the trend is apparent in all software tested. Liu et al. [26] found a higher number of false positive SNVs in multi sample results (SAM, UG and glfTools; [31]), whereby this observation was most pronounced in SAM. Cheng et al. [18] observed that the number of false positives decreased with increasing read depth, while UG showed the lowest false positive rate of all tested software.The largest difference between single and multi sample methods was observed in SNV concordance (Figure 8). Because homozygous reference loci show no difference to the reference genome, SNV concordance alone is somewhat misleading, as such loci are inherently not identified (they are not “variant”). This has a visible effect on the overall average concordance. SNV concordance by array genotype is therefore a better measure (Figure 8b). For homozygote reference loci, single sample methods provide no information whatsoever (this may be alleviated by the “emit all” option of the GATK, however this is computationally unfeasible for larger data sets). Multi sample methods identify some of the homozygous reference loci as such, however concordance reached only 83.8-88.2%, which indicated that a sample size of n = 65 is too small to have all the loci covered (at least one animal in the sample space must have a variant locus for the locus to be included). Once again, the “emit all” option of the GATK could be used, but the computational cost in a multi sample setting is even higher than that in single sample analysis. There is also visible discrepancy between single and multi sample results with respect to heterozygous loci, though this discrepancy is considerably smaller than for homozygote reference loci. SAM showed the highest SNV concordance for heterozygotes. For homozygote alternative loci, SNV concordance approached 100% for SAM, UG and HC, with slightly lower concordance observed in PL.Genotype concordance was very high in both single and multi sample results (Figure 9); depending on software, genotype concordance for single sample results was between 99.2-99.3% and between 99.1-99.3% for multi sample methods. Once a polymorphic locus is determined as such, all software applications perform very well in deciphering the correct genotype.

The use of multi sample variant detection to identify SNVs improved NRS but worsened NRD compared to single sample variant detection. SNV and genotype concordance improved when multi sample methods were applied. This effect was most pronounced in SNV concordance of homozygous reference genotypes and less pronounced in heterozygous genotypes, whereas both single and multi sample methods identified homozygote alternative genotypes equally well.

## Conclusions

The objective of this study was to investigate which methods and software work best for detection of high quality genetic variants using NGS data in cattle. We conclude that InDel realignment and base quality score recalibration have only slight effects on the number and quality of variants identified with the currently available resources for cattle and are costly with respect to computation time. The SNV concordance between variants identified using NGS data and array-based data was higher for multi sample methods than for single sample calling methods, although this was due mainly to the lack of homozygous reference genotypes in single sample results. The quality of SNVs identified (measured as the Ti/Tv ratio) using single sample methods was higher than that of multi sample calling for PL and UG and slightly lower for SAM, whereby a consensus approach using results of different software generally provides the highest variant quality. Computation time for single and multi sample methods was similar when calculated on a per-sample basis. These findings can serve as a reference for variant detection pipeline development in various organisms and help assess the value of preparatory steps in NGS pipelines for species with lower-quality reference genomes.

## Methods

### Sample selection

**Figure 10:**
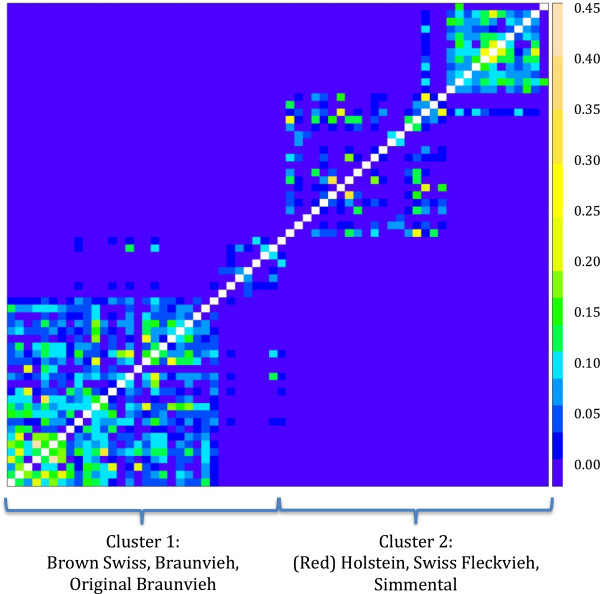
**Genomic relationship between the 65 sequenced animals.** Genomic relationship between the 65 sequenced animals was estimated using array genotypes (autosomal SNPs with known position) filtered separately for Cluster 1 (Brown Swiss, Braunvieh, Original Braunvieh; lower left corner of heat map) and Cluster 2 (Simmental, Swiss Fleckvieh, Holstein, Red Holstein; upper right corner of heat map). After filtering, the merged data set consisted of 38,317 common SNPs. The off-diagonals reflect the estimated pairwise identities by descent.

We selected 65 key ancestors of the main Swiss dairy populations with an iterative algorithm, which uses the numerator relationship matrix to rank animals according to percentage of genetic diversity they explain in a given population [32]. Specifically, *m* animals were selected with, where *p* is a vector that contains the percentage of gene pool diversity captured by *m* animals,  is a subset of the inverse numerator relationship matrix for *m* animals and *c* is a vector representing the average relationship of the *m* animals selected to the entire genotyped population. The subset of selected sires consisted of key Brown Swiss (n = 7), Braunvieh (n = 17) and Original Braunvieh (n = 8) Simmental (n = 12), Swiss Fleckvieh (n = 4) and (Red) Holstein (n = 17) ancestors that accounted for 74% of the genetic diversity of currently available genotyped populations of these breeds. All animals selected were male. Pairwise identity by descent was estimated for the merged dataset of the 65 sequenced sires by using the –genome function implemented in PLINK on array genotypes [33]. A heat map of the genomic relationships between key ancestors is given in Figure 10.

### DNA preparation, sequencing and alignment

Sequencing was done at the Helmholtz Center in Munich, Germany (German Research Center for Environmental Health Center) in collaboration with the Technical University of Munich. Genomic DNA was extracted from semen samples and sequenced using an Illumina HiSeq2000 (Illumina Inc., San Diego, CA, USA). Individual samples were sequenced on individual lanes of the flow cell. The bases of the resulting paired-end reads (101 bp) were identified with the Illumina BaseCaller; FASTQ files [34] were produced for downstream analysis of the sequence data.

Sequence alignment was done according to the sequence alignment guidelines for producing binary alignment mapping (BAM) files for the 1000 bull genomes project [35]. Briefly, the Burrows-Wheeler aligner (BWA version 0.6.1-r104 [36]) was used for read alignment to the University of Maryland Bovine reference assembly UMD3.1 build 137 [12]. Conversion from sequence alignment map format to sorted, indexed BAM files was done using SAMtools (version 0.1.18 [9]). PCR-duplicates were flagged using the MarkDuplicates option of the Picard software tools (version 1.61, [37]) and the MD5 message-digest algorithm values were examined to ensure correct data transfer from the sequencing lab to the computation center.

### Variant detection

Both single and multi sample methods for variant detection were applied. Single sample variant detection was performed with three different software applications: 1) SAM (MPileup / bcftools, version 0.1.18 [9]), 2) PL (version 0.5.2 [8]) and 3) the GATK UG (version 2.7-4-g6f46d11 [6, 7]) using default settings. In some cases, PL identified multi-SNV replacements (long alleles containing multiple bases) instead of SNVs. Because multiple SNVs may be embedded within a multi-SNV replacement, the number of SNVs recognised as such for PL was slightly lower than in other software. This problem was alleviated by applying the VariantsToAllelicPrimitives walker of the GATK, which splits multi-SNVs into their allelic primitive states.

For single sample variant identification, three levels of quality recalibration were compared for each animal and software application: a) no quality recalibration (RAW), b) local realignment around insertions and deletions using the GATK IndelRealigner walker (IR [7]) and c) local realignment around insertions and deletions using the GATK IndelRealigner walker followed by base quality score recalibration using the GATK BaseRecalibrator walker (IR + BQSR [7]). For IR and IR + BQSR, a set of 256,831 known InDels mapped uniquely to UMD3.1 and that passed the Ensembl quality control process was used to decrease computation time (Ensembl release 74, 2013). Reads surrounding InDels annotated by BWA but not included in the known InDel list were also used to identify further targets for realignment. Pipeline commands are available in Additional file 5. Multi sample variant detection was performed with the same three software applications above as well as with the GATK HC (version 2.7-4-g6f46d11 [6, 7]) using default settings. The HC is recommended by the GATK [14], but was not included in single sample variant identification due to excessive computation time. Preparatory local InDel realignment and base quality score recalibration were conducted for all 4 multi-sample analyses as described above (IR + BQSR). A schematic overview of methods and preparatory steps investigated in this study is given in Additional file 6: Figure S1.

### Computation specifications

Computation time required for preparatory steps and variant detection of a 5Mbp portion of BTA24 (single and multi sample methods) was tested on an Intel Xeon E5-2650 machine with two eight-core processors and a total of 256Gb RAM. No parallelism options were used for time calculations. For the whole genome analysis, single sample variant identification was done on a 24-node cluster at Iowa State University. Each computational node has two six-core processors, 64Gb of RAM, and 1 TB scratch space, for a total of 288 processors and 1536Gb RAM. Chromosomes were split into regions of similar size for parallelization; one chromosome was run per core and optimal threading options in GATK were implemented. Multi sample variant identification was done on the CyEnce cluster of Iowa State University, which includes 288 nodes with 128Gb RAM each.

### Concordance with high and medium density SNV arrays

The accuracy and completeness of SNVs identified in NGS data were assessed by comparing them to genotypes of the same animals generated with either the Illumina BovineHD BeadChip® (48 animals) or the Illumina BovineSNP50 v1 DNA Analysis BeadChip® (17 animals) (Illumina Inc., San Diego, CA, USA). The high-density array contained 774,660 SNVs mapped to the 29 autosomal chromosomes and the X chromosome (3,302 mitochondrial, Y-chromosomal and non-positioned SNVs were excluded). For the high-density array, 772,821 SNVs were assigned refSNP cluster ID numbers from dbSNP build 137 [24] using SNPchiMp [38]. After passing the Ensembl quality control process (includes plausibility checks on map positions, alleles of reference SNVs, alleles in dbSNP submissions, external failure classifications, etc., [39]), reference and alternative allele information for 673,396 remaining SNVs was included using the variant calling format files (VCF) available from the National Center for Biotechnology Information [40]. The medium density 50 K array originally contained 54,001 SNVs; after exclusion of unmapped SNVs, assignment of refSNP cluster IDs [24] and Ensembl quality control [39], reference and alternative allele information for 34,419 SNVs remained for analysis. The forward-forward coding from the Illumina final reports was used to compare all available array genotypes with the sequence-derived genotypes for single and multi sample variant detection.

Four measures of concordance and discrepancy between array data and NGS data were calculated as described in Table 1. The set of 673,396 SNVs on the Illumina BovineHD BeadChip® (or the set of 34,419 SNVs on the BovineSNP50 v1 DNA Analysis BeadChip®) was considered the total sample space. No differentiation was made between homozygous reference sites and those sites not identified due to low coverage (i.e. all non-polymorphic (non-variant) sites were considered homozygous reference). SNV concordance was calculated by adding the number of homozygote reference, heterozygote and homozygous alternative genotypes identified in the NGS-based data and dividing this sum by the number of SNVs in the array-based data (the total sample space). Genotype concordance was calculated as the number of correctly identified NGS-based genotypes divided by the number of homozygote reference, heterozygote and homozygous alternative genotypes identified in the NGS-based data. NRS and NRD were calculated as proposed by [7] and applied in [30]. NRS measures the proportion of variant loci identified in the NGS-based data also identified as variants in the array-based data. An NRS of one indicates perfect concordance of variant loci found in the NGS variant set and in the array. NRD represents the proportion of differing genotypes between the NGS variant set and the array. As the NRD represents discrepancy between the NGS variant set and the array, the NRD value should be as close to zero as possible. These measures were calculated chromosome-wise for both single and multi sample results.

### Availability of supporting data

All DNA references used were taken from the publically available bovine assembly UMD3.1 available for download from http://www.1000bullgenomes.com/. The identified variants were submitted to the Database of Single Nucleotide Polymorphisms (dbSNP) and are available from: http://www.ncbi.nlm.nih.gov/SNP/.

### Ethics statement

No animal experiments were performed.

## Electronic supplementary material

Additional file 1: **Alignment and coverage.** Total number of lanes, libraries/pool, reads, number of duplicates, number of mapped reads, net number of mapped reads, net number of bases, and net average coverage per animal. (PDF 54 KB)

Additional file 2: **Variant counts by animal.** a) Number of single nucleotide polymorphisms (SNPs) identified per animal using different software (Platypus, Samtools and UnifiedGenotyper) and various pre-variant identification processing steps. b) Number of insertions and deletions (INDELs) identified per animal using different software (Platypus, Samtools and UnifiedGenotyper) and various pre-variant identification processing steps. c) Number of multiallelic sites identified per animal using different software (Platypus, Samtools and UnifiedGenotyper) and various pre-variant identification processing steps. (PDF 77 KB)

Additional file 3: **Concordance with the Illumina BovineSNP50 v1 DNA Analysis BeadChip® (n = 17).** a) Non-reference sensitivity (NRS) for single nucleotide variants identified using Platypus (Primitives), Samtools, UnifiedGenotyper and Haplotype Caller (single and multi sample variant identification) using variants identified with the Illumina BovineSNP50 v1 DNA Analysis BeadChip® as a gold standard (BTA1-BTA29). b) Non-reference discrepancy (NRD) for single nucleotide variants identified using Platypus (Primitives), Samtools, UnifiedGenotyper and Haplotype Caller (single and multi sample variant identification) using variants identified with the Illumina BovineSNP50 v1 DNA Analysis BeadChip® as a gold standard (BTA1-BTA29). c) Single nucleotide variant concordance identified using Platypus Primitives), Samtools, UnifiedGenotyper and Haplotype Caller (single and multi sample variant identification) using variants identified with the Illumina BovineSNP50 v1 DNA Analysis BeadChip® as a gold standard (BTA1‒BTA29). d) Single nucleotide variant concordance by genotypes identified using Platypus (Primitives), Samtools, UnifiedGenotyper and Haplotype Caller (single and multi sample variant identification) using variants identified with the Illumina BovineSNP50 v1 DNA Analysis BeadChip® as a gold standard (BTA1‒BTA29). e) Concordance for homozygous reference genotypes identified using Platypus (Primitives), Samtools, UnifiedGenotyper and Haplotype Caller (single and multi sample variant identification) using variants identified with the Illumina BovineSNP50 v1 DNA Analysis BeadChip® as a gold standard (BTA1‒BTA29). f) Concordance for heterozygous genotypes identified using Platypus (Primitives), Samtools, UnifiedGenotyper and Haplotype Caller (single and multi sample variant identification) using variants identified with the Illumina BovineSNP50 v1 DNA Analysis BeadChip® as a gold standard (BTA1‒BTA29). g) Concordance for homozygous alternative genotypes identified using Platypus (Primitives), Samtools, UnifiedGenotyper and Haplotype Caller (single and multi sample variant identification) using variants identified with the Illumina BovineSNP50 v1 DNA Analysis BeadChip® as a gold standard (BTA1‒BTA29). (PDF 82 KB)

Additional file 4: **Concordance with the Illumina Concordance with the Illumina BovineHD BeadChip® (n = 48).** a) Non-reference sensitivity (NRS) for single nucleotide variants identified using Platypus (Primitives), Samtools, UnifiedGenotyper and Haplotype Caller (single and multi sample variant identification) using variants identified with the Illumina BovineHD BeadChip® as a gold standard (BTA1-BTA29). b) Non-reference discrepancy (NRD) for single nucleotide variants identified using Platypus (Primitives), Samtools, UnifiedGenotyper and Haplotype Caller (single and multi sample variant identification) using variants identified with the Illumina BovineHD BeadChip® as a gold standard (BTA1-BTA29). c) Single nucleotide variant concordance identified using Platypus Primitives), Samtools, UnifiedGenotyper and Haplotype Caller (single and multi sample variant identification) using variants identified with the Illumina BovineHD BeadChip® as a gold standard (BTA1-BTA29). d) Single nucleotide variant concordance by genotypes identified using Platypus (Primitives), Samtools, UnifiedGenotyper and Haplotype Caller (single and multi sample variant identification) using variants identified with the Illumina BovineHD BeadChip® as a gold standard (BTA1-BTA29). e) Concordance for homozygous reference genotypes identified using Platypus (Primitives), Samtools, UnifiedGenotyper and Haplotype Caller (single and multi sample variant identification) using variants identified with the Illumina BovineHD BeadChip® as a gold standard (BTA1-BTA29). f) Concordance for heterozygous genotypes identified using Platypus (Primitives), Samtools, UnifiedGenotyper and Haplotype Caller (single and multi sample variant identification) using variants identified with the Illumina BovineHD BeadChip® as a gold standard (BTA1-BTA29). g) Concordance for homozygous alternative genotypes identified using Platypus (Primitives), Samtools, UnifiedGenotyper and Haplotype Caller (single and multi sample variant identification) using variants identified with the Illumina BovineHD BeadChip® as a gold standard (BTA1-BTA29). (PDF 123 KB)

Additional file 5: **PipelineCode.** (PDF 52 KB)

Additional file 6: **A schematic overview of variant identification pipelines and methods examined in this study.** (PDF 241 KB)
