# Supplementary material for: Evaluation of variant identification methods for whole genome sequencing data in dairy cattle
Source: BMC Genomics. 2014 Nov 1;15(1):948. doi: 10.1186/1471-2164-15-948 (PMC4289218; doi:10.1186/1471-2164-15-948)
Supplement: Supplementary file 1 — Additional file 1: Alignment and coverage. Total number of lanes, libraries/pool, reads, number of duplicates, number of mapped reads, net number of mapped reads, net number of bases, and net average coverage per animal. (PDF 54 KB) [file 12864_2014_6640_MOESM1_ESM.pdf]

Additional File S1

Total number of lanes, libraries/pool, reads, number of duplicates, number of mapped reads, net number of mapped reads, net number of bases, and net average coverage per animal

Animal	Lanes	Libraries/ Pool	Total Reads	Duplicates	Duplicates (%)	Mapped reads	Mapped reads (%)	Net Mapped	Net Mapped (%)	Net Bases	Net Average coverage
1	1	1	295'580'486	12'414'460	4.20%	290'311'434	98.22%	278'118'276	94.09%	28.09GB	10.56
2	1	1	295'561'924	16'489'862	5.58%	290'048'062	98.13%	273'865'827	92.66%	27.66GB	10.4
3	1	1	322'860'286	23'317'514	7.22%	316'838'066	98.13%	293'955'487	91.05%	29.69GB	11.16
4	1	1	350'949'790	24'105'371	6.87%	334'398'261	95.28%	311'429'750	88.74%	31.45GB	11.82
5	1	1	367'136'492	30'855'041	8.40%	360'075'825	98.08%	329'814'180	89.83%	33.31GB	12.52
6	1	1	295'848'034	20'665'451	6.99%	288'020'725	97.35%	267'902'024	90.55%	27.06GB	10.17
7	1	1	321'547'492	25'700'126	7.99%	315'251'226	98.04%	290'054'338	90.21%	29.30GB	11.01
8	1	1	358'267'014	32'598'052	9.10%	351'423'704	98.09%	319'448'312	89.16%	32.26GB	12.13
9	1	1	323'459'806	29'993'966	9.27%	316'639'213	97.89%	287'277'711	88.81%	29.02GB	10.91
10	1	1	380'505'218	28'530'749	7.50%	362'576'982	95.29%	335'390'514	88.14%	33.87GB	12.73
11	1	1	374'123'018	26'047'238	6.96%	358'537'622	95.83%	333'575'472	89.16%	33.69GB	12.67
12	1	1	346'571'890	28'638'379	8.26%	332'462'454	95.93%	304'989'984	88.00%	30.80GB	11.58
13	1	1	357'882'212	23'793'828	6.65%	342'854'400	95.80%	320'059'697	89.43%	32.33GB	12.15
14	1	1	429'031'870	38'350'795	8.94%	418'026'581	97.43%	380'659'539	88.73%	38.45GB	14.45
15	1	1	334'530'342	26'053'258	7.79%	327'678'976	97.95%	302'159'303	90.32%	30.52GB	11.47
16	1	1	394'265'364	33'805'876	8.57%	380'454'030	96.50%	347'832'393	88.22%	35.13GB	13.21
17	1	1	402'835'694	28'530'659	7.08%	386'911'269	96.05%	359'508'450	89.24%	36.31GB	13.65
18	1	1	353'899'990	27'209'419	7.69%	342'656'909	96.82%	316'311'909	89.38%	31.95GB	12.01
19	1	1	371'803'348	30'174'578	8.12%	360'046'390	96.84%	330'825'976	88.98%	33.41GB	12.56
20	1	1	378'140'382	36'027'911	9.53%	365'938'834	96.77%	331'073'444	87.55%	33.44GB	12.57
21	1	1	380'555'278	32'353'670	8.50%	366'329'482	96.26%	335'185'247	88.08%	33.85GB	12.73
22	1	1	377'837'180	29'667'736	7.85%	364'738'746	96.53%	336'099'498	88.95%	33.95GB	12.76
23	1	1	440'501'220	34'917'888	7.93%	426'155'132	96.74%	392'374'437	89.07%	39.63GB	14.9
24	1	1	397'596'086	32'260'058	8.11%	344'309'208	86.60%	316'372'728	79.57%	31.95GB	12.01
25	1	1	332'608'130	27'254'392	8.19%	321'603'753	96.69%	295'251'076	88.77%	29.82GB	11.21
26	1	1	337'537'066	22'946'054	6.80%	325'865'621	96.54%	303'713'002	89.98%	30.68GB	11.53
27	1	1	326'241'602	25'391'462	7.78%	317'037'013	97.18%	292'361'946	89.62%	29.53GB	11.1
28	1	1	338'979'966	23'359'293	6.89%	328'950'606	97.04%	306'282'442	90.35%	30.93GB	11.63
29	1	1	395'443'786	33'056'661	8.36%	369'192'282	93.36%	338'330'085	85.56%	34.17GB	12.85
30	1	1	369'308'954	23'167'117	6.27%	358'423'850	97.05%	335'939'566	90.96%	33.93GB	12.76
31	2	1	412'164'190	21'873'004	5.31%	399'117'004	96.83%	377'936'397	91.70%	38.17GB	14.35
32	1	1	371'220'770	22'775'174	6.14%	355'651'737	95.81%	333'831'756	89.93%	33.72GB	12.68
33	1	1	390'876'014	25'485'590	6.52%	377'391'747	96.55%	352'785'348	90.26%	35.63GB	13.4
34	1	1	432'540'294	25'610'877	5.92%	415'228'503	96.00%	390'642'664	90.31%	39.45GB	14.83
35	1	1	338'164'548	18'847'516	5.57%	314'573'120	93.02%	297'040'466	87.84%	30.00GB	11.28
36	1	1	440'147'704	26'083'256	5.93%	422'997'681	96.10%	397'930'739	90.41%	40.19GB	15.11
37	1	1	411'227'552	25'222'260	6.13%	396'267'299	96.36%	371'962'612	90.45%	37.57GB	14.12
38	1	1	388'308'270	26'135'673	6.73%	381'395'438	98.22%	355'725'044	91.61%	35.93GB	13.51
39	1	1	367'952'396	21'373'543	5.81%	355'326'332	96.57%	334'686'209	90.96%	33.80GB	12.71
40	1	1	401'623'036	26'559'101	6.61%	386'859'835	96.32%	361'277'016	89.95%	36.49GB	13.72
41	1	1	337'396'372	20'368'337	6.04%	330'879'917	98.07%	310'904'973	92.15%	31.40GB	11.81
42	1	1	320'012'770	21'709'825	6.78%	310'638'092	97.07%	289'564'250	90.49%	29.25GB	10.99
43	1	1	332'501'826	23'008'232	6.92%	323'201'508	97.20%	300'836'833	90.48%	30.38GB	11.42
44	6	12	346'256'450	22'977'071	6.64%	335'827'000	96.99%	313'542'012	90.55%	31.67GB	11.91
45	1	1	341'124'056	22'703'753	6.66%	330'594'542	96.91%	308'591'588	90.46%	31.17GB	11.72
46	1	1	349'217'388	24'508'651	7.02%	338'102'604	96.82%	314'374'007	90.02%	31.75GB	11.94
47	1	1	348'846'776	24'049'498	6.89%	338'624'856	97.07%	315'280'057	90.38%	31.84GB	11.97
48	1	1	350'885'702	22'997'104	6.55%	340'266'635	96.97%	317'965'506	90.62%	32.11GB	12.07
49	1	1	330'470'486	22'205'093	6.72%	320'008'623	96.83%	298'506'487	90.33%	30.15GB	11.33
50	1	1	362'116'512	23'912'590	6.60%	350'578'369	96.81%	327'427'707	90.42%	33.07GB	12.43
51	1	1	385'380'604	30'726'662	7.97%	373'071'536	96.81%	343'326'285	89.09%	34.68GB	13.04
52	1	1	348'083'344	24'136'745	6.93%	337'474'958	96.95%	314'073'818	90.23%	31.72GB	11.93
53	1	1	329'744'000	28'886'851	8.76%	323'302'934	98.05%	294'980'345	89.46%	29.79GB	11.2
54	1	1	356'372'446	23'981'810	6.73%	345'679'716	97.00%	322'417'465	90.47%	32.56GB	12.24
55	1	1	386'075'902	26'171'448	6.78%	373'046'343	96.63%	347'758'147	90.08%	35.12GB	13.2
56	1	1	343'160'138	21'971'011	6.40%	332'791'639	96.98%	311'484'477	90.77%	31.46GB	11.83
57	1	1	326'230'154	23'248'005	7.13%	319'948'215	98.07%	297'147'877	91.09%	30.01GB	11.28
58	1	1	346'927'160	25'998'256	7.49%	340'488'892	98.14%	314'973'111	90.79%	31.81GB	11.96
59	1	1	342'210'888	24'033'181	7.02%	335'133'047	97.93%	311'596'937	91.05%	31.47GB	11.83
60	1	1	294'467'464	18'452'301	6.27%	287'968'665	97.79%	269'923'600	91.66%	27.26GB	10.25
61	1	1	349'138'122	36'991'017	10.59%	342'793'225	98.18%	306'474'447	87.78%	30.95GB	11.64
62	1	1	297'626'546	23'881'799	8.02%	291'983'922	98.10%	268'554'892	90.23%	27.12GB	10.2
63	1	1	305'946'644	19'378'251	6.33%	299'568'937	97.92%	280'594'641	91.71%	28.34GB	10.65
64	1	1	350'562'616	23'351'515	6.66%	344'021'368	98.13%	321'105'576	91.60%	32.43GB	12.19
65	1	1	518'006'588	47'733'942	9.21%	505'644'784	97.61%	459'049'974	88.62%	46.36GB	17.43
**Average**	**1**	**1**	**360'067'625**	**26'077'320**	**7.22%**	**348'310'857**	**96.79%**	**323'083'629**	**89.80%**	**32.63GB**	**12.27**
