# Supplementary material for: Evaluation of variant identification methods for whole genome sequencing data in dairy cattle
Source: BMC Genomics. 2014 Nov 1;15(1):948. doi: 10.1186/1471-2164-15-948 (PMC4289218; doi:10.1186/1471-2164-15-948)
Supplement: Supplementary file 2 — Additional file 2: Variant counts by animal. a) Number of single nucleotide polymorphisms (SNPs) identified per animal using different software (Platypus, Samtools and UnifiedGenotyper) and various pre-variant identification processing steps. b) Number of insertions and deletions (INDELs) identified per animal using different software (Platypus, Samtools and UnifiedGenotyper) and various pre-variant identification processing steps. c) Number of multiallelic sites identified per animal using different software (Platypus, Samtools and UnifiedGenotyper) and various pre-variant identification processing steps. (PDF 77 KB) [file 12864_2014_6640_MOESM2_ESM.pdf]

Additional File S2

Variant counts by individual

a) Number of **single nucleotide polymorphisms (SNPs)** identified per animal using different software (Platypus, Samtools and UnifiedGenotyper) and various pre-variant identification processing steps

	Platypus			Platypus Primitives			Samtools			UnifiedGenotyper		
**Animal**	**Raw**	**IR**	**IR+BQSR**	**Raw**	**IR**	**IR+BQSR**	**Raw**	**IR**	**IR+BQSR**	**Raw**	**IR**	**IR+BQSR**
1	5,016,667	5,015,057	4,890,140	5,977,075	5,972,518	5,853,927	5,880,556	5,880,299	6,058,310	6,406,813	6,362,098	6,159,109
2	4,855,811	4,854,412	4,715,137	5,776,387	5,772,356	5,638,098	5,699,611	5,699,307	5,875,913	6,203,657	6,162,578	5,940,660
3	4,953,675	4,951,831	4,826,769	5,882,214	5,877,532	5,762,130	5,830,774	5,830,565	5,998,515	6,334,822	6,289,275	6,095,800
4	4,946,810	4,945,167	4,786,721	5,871,706	5,867,229	5,717,910	5,927,460	5,927,074	6,097,186	6,397,684	6,357,123	6,150,747
5	5,142,233	5,140,656	5,024,902	6,127,895	6,123,114	6,010,297	5,947,651	5,947,445	6,145,229	6,557,924	6,505,941	6,342,897
6	4,889,049	4,887,684	4,682,423	5,792,958	5,789,269	5,572,301	5,714,658	5,714,489	5,896,134	6,232,569	6,196,767	5,891,715
7	5,005,092	5,003,690	4,858,342	5,952,950	5,949,049	5,806,219	5,821,489	5,821,403	6,013,172	6,384,561	6,340,494	6,115,033
8	4,989,136	4,987,423	4,869,471	5,946,710	5,941,829	5,824,999	5,785,866	5,785,667	5,973,730	6,367,556	6,316,416	6,150,483
9	5,035,690	5,034,153	4,882,561	6,003,365	5,998,765	5,850,755	5,863,912	5,863,706	6,059,081	6,413,722	6,371,411	6,151,167
10	4,910,882	4,909,020	4,778,194	5,830,816	5,825,973	5,703,559	5,842,687	5,842,378	6,007,583	6,335,539	6,289,215	6,130,678
11	5,092,633	5,090,966	4,959,532	6,044,118	6,039,565	5,916,341	6,024,264	6,023,923	6,193,037	6,536,909	6,492,415	6,330,004
12	4,977,247	4,975,731	4,822,523	5,907,358	5,903,192	5,755,378	5,898,162	5,897,842	6,070,272	6,391,952	6,351,886	6,140,046
13	5,032,739	5,031,133	4,881,221	5,972,700	5,968,284	5,824,275	5,967,614	5,967,360	6,133,537	6,458,235	6,415,877	6,221,987
14	5,264,256	5,262,468	5,148,058	6,276,221	6,271,125	6,158,668	6,102,472	6,102,283	6,298,958	6,715,411	6,661,710	6,540,103
15	5,000,171	4,998,655	4,846,457	5,936,352	5,932,302	5,781,352	5,847,476	5,847,350	6,034,579	6,382,614	6,337,825	6,125,929
16	5,193,559	5,191,839	5,053,263	6,197,114	6,192,267	6,049,152	6,021,560	6,021,193	6,217,837	6,626,870	6,576,697	6,410,660
17	5,295,588	5,293,740	5,165,700	6,319,949	6,314,788	6,183,915	6,133,679	6,133,326	6,333,760	6,761,773	6,708,309	6,556,434
18	5,116,690	5,114,969	4,971,914	6,097,520	6,092,795	5,949,780	5,955,914	5,955,735	6,150,776	6,539,322	6,492,581	6,298,592
19	5,000,389	4,998,651	4,850,078	5,967,428	5,962,478	5,803,682	5,814,819	5,814,539	6,011,493	6,406,782	6,356,060	6,171,308
20	5,035,860	5,034,204	4,903,261	6,012,581	6,007,662	5,876,149	5,849,493	5,849,209	6,044,559	6,438,052	6,388,036	6,223,609
21	5,057,080	5,055,359	4,928,596	6,035,223	6,030,135	5,904,189	5,871,143	5,870,933	6,066,396	6,465,969	6,414,368	6,255,479
22	5,138,605	5,136,900	5,006,072	6,135,076	6,130,150	5,999,701	5,967,232	5,966,978	6,166,868	6,567,430	6,516,612	6,349,978
23	5,067,785	5,065,785	4,978,193	6,062,920	6,057,303	5,971,947	5,884,079	5,883,726	6,075,668	6,503,427	6,440,996	6,342,573
24	4,832,018	4,830,515	4,702,883	5,747,237	5,742,984	5,617,635	5,694,372	5,694,000	5,864,109	6,201,207	6,157,091	5,985,655
25	4,926,807	4,925,419	4,770,606	5,854,533	5,850,392	5,696,416	5,812,419	5,812,216	5,995,778	6,322,216	6,278,462	6,047,170
26	4,964,459	4,962,882	4,813,130	5,899,316	5,895,116	5,744,234	5,849,117	5,848,851	6,026,247	6,361,323	6,317,136	6,098,610
27	4,799,418	4,797,944	4,650,758	5,710,119	5,706,246	5,557,392	5,664,866	5,664,561	5,845,490	6,173,494	6,128,972	5,903,219
28	4,864,595	4,863,036	4,721,695	5,784,970	5,780,589	5,637,191	5,723,681	5,723,418	5,901,428	6,246,782	6,198,545	5,990,176
29	4,960,893	4,959,214	4,812,249	5,919,275	5,914,207	5,756,433	5,749,840	5,749,706	5,952,157	6,344,549	6,290,560	6,081,960
30	5,098,208	5,096,419	4,969,401	6,070,554	6,065,765	5,940,748	5,983,543	5,983,119	6,164,077	6,531,710	6,482,078	6,316,356
31	5,119,596	5,117,671	5,019,171	6,100,678	6,095,355	5,999,893	5,992,515	5,992,168	6,169,260	6,572,921	6,514,031	6,389,013
32	5,064,024	5,062,359	4,931,768	6,032,553	6,028,108	5,898,108	5,941,817	5,941,539	6,124,161	6,493,626	6,445,096	6,277,694
33	4,916,334	4,914,506	4,899,389	5,868,508	5,863,440	5,870,834	5,752,211	5,751,774	5,927,514	6,306,964	6,251,855	6,129,011
34	5,021,958	5,019,963	4,937,582	6,000,703	5,995,439	5,918,150	5,877,392	5,877,000	6,051,434	6,467,763	6,406,073	6,302,421
35	4,858,956	4,857,376	4,714,464	5,776,318	5,771,877	5,631,852	5,732,123	5,731,907	5,912,647	6,238,544	6,193,058	5,968,131
36	5,172,226	5,170,258	5,084,943	6,164,988	6,159,506	6,080,233	6,054,344	6,054,029	6,227,943	6,646,227	6,585,547	6,477,198
37	5,159,473	5,157,697	5,064,269	6,155,882	6,150,950	6,064,823	6,036,198	6,035,874	6,210,160	6,613,157	6,558,147	6,444,374
38	5,089,588	5,087,732	4,985,305	6,063,294	6,058,458	5,959,938	5,919,361	5,919,003	6,093,631	6,489,909	6,437,410	6,312,554
39	4,998,794	4,996,836	4,878,279	5,948,065	5,942,899	5,827,939	5,883,415	5,883,177	6,060,945	6,418,532	6,364,282	6,192,383
40	5,007,756	5,005,929	4,914,663	5,972,089	5,967,084	5,884,908	5,870,854	5,870,464	6,038,335	6,429,975	6,377,541	6,265,630
41	5,118,254	5,116,932	4,979,293	6,082,536	6,078,683	5,945,388	5,967,049	5,966,863	6,162,048	6,534,220	6,488,361	6,267,111
42	5,088,501	5,087,053	4,927,094	6,046,923	6,042,596	5,888,614	5,943,683	5,943,383	6,133,531	6,485,976	6,447,224	6,205,789
43	4,999,076	4,997,526	4,854,781	5,954,354	5,950,088	5,810,743	5,823,480	5,823,266	6,016,176	6,383,210	6,339,740	6,136,418
44	5,105,592	5,104,131	4,947,805	6,075,028	6,070,912	5,914,874	5,939,826	5,939,645	6,134,860	6,501,909	6,458,406	6,251,826
45	5,004,405	5,002,850	4,845,846	5,953,846	5,949,599	5,791,639	5,832,239	5,832,034	6,027,286	6,382,295	6,337,522	6,132,482
46	5,017,878	5,016,387	4,857,543	5,975,370	5,971,024	5,809,096	5,848,642	5,848,454	6,040,234	6,403,234	6,358,715	6,154,461
47	5,006,248	5,004,649	4,863,100	5,956,269	5,951,844	5,810,909	5,835,747	5,835,564	6,025,576	6,395,941	6,347,850	6,158,815
48	5,043,255	5,041,650	4,909,135	6,001,931	5,997,341	5,866,488	5,872,042	5,871,860	6,066,331	6,444,440	6,394,877	6,216,576
49	4,991,674	4,990,225	4,840,012	5,927,916	5,923,833	5,776,938	5,834,808	5,834,609	6,021,037	6,367,573	6,325,381	6,111,548
50	5,037,646	5,036,122	4,900,859	6,001,876	5,997,461	5,863,620	5,856,116	5,855,940	6,047,818	6,425,561	6,377,140	6,210,167
51	4,963,327	4,961,730	4,848,807	5,918,382	5,913,645	5,805,062	5,770,803	5,770,578	5,953,262	6,339,736	6,287,700	6,150,155
52	5,033,470	5,031,935	4,892,522	5,980,198	5,976,041	5,840,513	5,910,905	5,910,557	6,084,480	6,438,229	6,394,422	6,206,227
53	4,910,490	4,909,046	4,776,739	5,836,912	5,832,925	5,703,799	5,707,825	5,707,692	5,887,876	6,250,918	6,208,801	6,009,977
54	4,951,489	4,949,769	4,821,894	5,891,712	5,887,106	5,762,619	5,802,317	5,802,002	5,974,259	6,332,456	6,285,509	6,119,237
55	5,038,957	5,037,220	4,922,979	6,001,053	5,996,167	5,886,989	5,903,406	5,903,050	6,079,785	6,472,303	6,420,459	6,277,525
56	4,853,696	4,851,936	4,729,482	5,769,860	5,765,575	5,647,279	5,701,207	5,700,886	5,870,570	6,219,838	6,173,448	5,997,495
57	4,919,842	4,918,285	4,780,465	5,851,494	5,847,376	5,713,202	5,753,647	5,753,371	5,931,797	6,277,317	6,235,715	6,036,121
58	4,996,549	4,994,898	4,872,256	5,953,516	5,949,156	5,831,008	5,831,861	5,831,554	6,008,542	6,374,437	6,328,875	6,162,145
59	4,929,231	4,927,448	4,810,540	5,884,531	5,879,378	5,765,595	5,761,187	5,760,855	5,944,537	6,313,606	6,263,713	6,103,430
60	4,733,719	4,732,337	4,573,092	5,620,333	5,616,563	5,459,659	5,605,166	5,604,932	5,782,333	6,093,594	6,055,092	5,787,116
61	4,987,980	4,986,212	4,860,192	5,939,206	5,934,416	5,813,541	5,838,422	5,838,116	6,019,353	6,382,733	6,334,421	6,158,834
62	4,707,215	4,705,723	4,573,144	5,588,937	5,584,857	5,459,585	5,547,885	5,547,672	5,717,320	6,026,428	5,986,605	5,755,305
63	4,903,145	4,901,802	4,751,828	5,832,866	5,829,206	5,679,751	5,725,551	5,725,368	5,914,644	6,267,366	6,227,138	5,976,155
64	5,000,173	4,998,515	4,882,205	5,953,547	5,948,970	5,838,286	5,847,718	5,847,526	6,022,861	6,401,018	6,354,565	6,189,695
65	5,093,421	5,091,627	5,032,990	6,095,733	6,091,556	6,033,759	5,915,436	5,833,568	6,005,723	6,463,311	6,426,875	6,365,177
**Average**	**5,004,738**	**5,003,096**	**4,873,149**	**5,958,308**	**5,953,760**	**5,826,468**	**5,854,886**	**5,853,367**	**6,036,310**	**6,404,094**	**6,356,756**	**6,175,636**

Raw= no InDel realignment or base quality score recalibration

IR= InDel realignment

IR+BQSR= InDel realignment followed by base quality score recalibration

b) Number of **insertions and deletions (INDELS)** identified per animal using different software (Platypus, Samtools and UnifiedGenotyper) and various pre-variant identification processing steps

	Platypus			Platypus Primitives			Samtools			UnifiedGenotyper		
**Animal**	**Raw**	**IR**	**IR+BQSR**	**Raw**	**IR**	**IR+BQSR**	**Raw**	**IR**	**IR+BQSR**	**Raw**	**IR**	**IR+BQSR**
1	767,721	809,167	821,496	751,269	791,089	803,022	606,715	614,842	619,265	457,745	526,249	526,853
2	738,808	780,478	791,090	720,361	760,375	770,590	584,082	593,061	597,601	421,138	486,952	487,565
3	752,755	794,714	809,874	734,431	774,810	789,428	603,012	611,820	615,793	458,681	529,639	530,224
4	719,046	762,814	776,578	702,923	745,262	758,621	590,833	600,597	604,941	423,793	493,917	494,483
5	834,663	870,148	881,191	812,857	846,647	857,220	643,710	649,800	654,809	552,702	623,706	624,696
6	782,776	820,635	816,972	763,926	800,350	796,906	591,535	598,720	605,080	429,662	494,341	494,938
7	804,926	842,184	851,311	784,610	820,290	829,086	617,026	623,549	628,830	481,627	550,417	551,243
8	811,205	846,120	855,303	790,365	823,761	832,624	630,114	636,281	640,975	532,018	600,536	601,335
9	811,216	847,971	857,938	791,661	826,826	836,442	620,919	627,494	632,852	475,978	543,093	543,914
10	724,429	767,263	780,679	707,318	748,615	761,580	600,481	609,771	613,662	465,878	538,410	538,956
11	760,730	803,119	816,712	742,366	783,217	796,330	615,354	624,241	628,281	477,744	550,785	551,271
12	742,308	785,287	797,413	724,130	765,560	777,307	594,844	603,902	608,111	437,071	505,562	506,046
13	747,793	790,683	802,974	730,341	771,735	783,654	604,703	613,991	618,328	454,552	526,052	526,550
14	831,714	869,109	880,143	810,485	846,190	856,823	653,812	659,450	664,116	569,649	642,135	642,848
15	780,140	819,553	828,446	760,786	798,673	807,266	609,840	616,924	621,877	463,545	531,840	532,459
16	815,609	853,271	859,369	796,360	832,541	838,470	637,285	643,984	649,221	533,956	605,429	606,195
17	835,346	873,261	881,142	814,395	850,707	858,339	651,585	658,305	663,392	560,284	634,061	634,878
18	801,840	840,758	849,849	782,625	819,968	828,760	624,973	632,177	637,188	496,977	567,450	568,273
19	790,711	828,636	831,426	770,650	806,964	809,682	620,475	627,797	633,543	511,421	581,172	581,927
20	794,522	832,659	842,301	774,195	810,777	820,068	623,771	630,769	635,801	511,165	580,356	581,202
21	798,874	836,703	845,811	777,876	814,100	822,876	627,309	634,279	639,241	521,233	591,027	591,844
22	807,344	845,218	854,512	787,210	823,499	832,479	632,107	639,313	644,327	521,790	592,740	593,500
23	823,314	857,767	869,369	801,193	833,976	845,099	652,288	658,349	662,489	599,892	668,090	668,866
24	743,247	782,764	794,331	724,674	762,644	773,783	595,450	603,980	608,412	466,202	532,861	533,443
25	756,188	796,478	806,637	738,063	776,885	786,714	599,615	607,844	612,828	454,471	521,763	522,389
26	762,982	803,262	813,339	745,270	784,082	793,853	604,997	613,184	617,903	468,738	536,793	537,344
27	740,155	779,740	789,011	722,034	760,095	769,060	589,191	597,627	602,269	451,169	517,248	517,880
28	756,569	795,591	805,440	737,959	775,529	785,041	602,855	610,966	615,564	480,232	547,333	548,047
29	821,393	853,238	850,942	800,436	830,806	828,801	638,533	644,311	649,979	565,629	629,432	630,277
30	786,097	825,949	837,066	767,210	805,571	816,312	626,864	635,370	639,910	513,989	585,160	585,857
31	796,781	835,435	847,838	776,492	813,573	825,517	642,267	649,885	653,967	571,485	642,805	643,563
32	778,736	817,907	829,456	759,494	797,151	808,262	620,521	628,554	633,133	506,110	576,322	576,924
33	769,499	806,874	898,245	749,640	785,419	872,959	622,276	629,803	633,931	534,360	602,602	603,265
34	790,519	828,264	841,797	769,481	805,644	818,645	640,685	648,110	651,851	580,364	650,410	651,153
35	751,834	792,018	803,239	733,711	772,428	783,256	597,276	605,680	610,161	457,270	524,689	525,325
36	807,916	846,187	859,875	786,792	823,314	836,452	650,271	657,829	661,993	589,548	661,856	662,513
37	801,160	839,912	854,281	781,441	818,630	832,426	639,502	646,891	650,906	556,223	628,611	629,380
38	791,831	829,928	842,053	771,929	808,387	820,070	626,951	634,159	638,373	540,285	610,480	611,198
39	781,867	821,394	833,833	762,661	800,618	812,627	627,549	634,852	639,064	524,694	595,406	595,903
40	776,911	816,085	830,634	757,160	794,699	808,670	621,041	628,659	632,583	529,663	600,845	601,566
41	832,490	868,145	878,559	811,410	845,462	855,459	634,583	641,111	646,142	523,497	592,397	593,267
42	799,815	839,989	851,442	780,004	818,550	829,593	609,084	616,077	621,167	442,646	511,101	511,918
43	794,880	833,045	843,541	774,432	810,961	821,094	613,503	620,466	625,471	472,582	540,866	541,633
44	808,850	846,982	855,265	789,063	825,672	833,693	619,206	625,887	631,303	483,714	552,898	553,728
45	792,204	830,717	837,900	772,507	809,442	816,384	611,158	617,784	623,033	472,988	540,808	541,533
46	793,053	831,295	836,889	774,008	810,776	816,192	611,288	617,670	623,036	476,668	544,751	545,462
47	795,492	833,324	842,165	774,626	810,804	819,355	619,740	626,279	631,146	494,608	563,235	563,913
48	805,463	842,707	852,752	784,474	820,097	829,741	627,810	634,418	639,087	510,841	579,927	580,665
49	785,830	825,237	835,068	766,328	804,195	813,685	606,498	613,036	617,717	454,554	522,763	523,440
50	801,623	839,370	848,859	781,316	817,404	826,507	619,984	626,227	631,109	502,940	572,561	573,291
51	797,227	833,941	845,381	777,268	812,392	823,413	623,027	629,090	633,579	526,451	595,709	596,567
52	770,406	811,253	822,638	751,332	790,592	801,568	607,195	615,710	620,235	465,367	535,954	536,548
53	788,370	825,727	834,996	768,587	804,481	813,438	603,889	610,492	615,208	471,852	539,174	539,975
54	759,296	799,364	810,376	740,805	779,381	789,990	603,147	611,277	615,691	475,580	545,533	546,240
55	774,558	814,357	826,533	754,626	792,837	804,548	618,018	625,864	630,199	512,739	584,677	585,358
56	745,084	784,864	796,651	726,541	764,777	776,125	593,616	601,845	605,971	465,547	533,489	534,109
57	757,117	797,378	808,144	738,119	776,851	787,257	593,894	601,998	606,592	446,875	513,652	514,333
58	769,448	809,126	820,525	750,864	789,021	800,021	606,803	614,649	619,163	478,361	547,190	547,884
59	764,645	803,680	814,797	745,693	783,186	793,916	611,083	619,141	623,586	494,912	562,515	563,215
60	727,618	769,266	778,749	709,209	749,219	758,368	571,595	580,112	584,848	399,924	463,995	464,547
61	767,400	807,807	820,489	747,528	786,285	798,514	612,639	620,837	625,159	483,170	552,914	553,575
62	718,739	760,451	773,350	701,141	741,270	753,712	572,800	581,526	585,623	408,623	474,397	474,996
63	795,182	831,402	839,325	775,098	809,819	817,479	608,376	615,252	620,334	468,056	532,639	533,433
64	772,123	812,205	825,542	753,222	791,761	804,583	610,074	617,688	622,017	491,035	561,259	562,021
65	845,224	872,651	884,959	822,189	848,139	859,926	662,669	658,224	662,497	680,713	729,438	729,014
**Average**	**782,363**	**821,120**	**832,689**	**762,880**	**800,074**	**811,226**	**616,220**	**623,535**	**628,192**	**496,203**	**564,960**	**565,642**

Raw= no InDel realignment or base quality score recalibration

IR= InDel realignment

IR+BQSR= InDel realignment followed by base quality score recalibration

c) Number of **multiallelic** sites identified per animal using different software (Platypus, Samtools and UnifiedGenotyper) and various pre-variant identification processing steps

	Platypus			Platypus Primitives			Samtools			UnifiedGenotyper		
**Animal**	**Raw**	**IR**	**IR+BQSR**	**Raw**	**IR**	**IR+BQSR**	**Raw**	**IR**	**IR+BQSR**	**Raw**	**IR**	**IR+BQSR**
1	40,879	40,838	35,849	40,704	40,664	35,733	6,844	6,848	9,004	2,521	2,491	1,960
2	36,322	36,274	31,171	35,892	35,835	30,848	7,693	7,688	9,605	2,240	2,202	1,674
3	39,667	39,636	34,878	39,275	39,245	34,587	7,073	7,060	9,675	2,534	2,484	1,963
4	38,038	38,015	32,183	37,663	37,634	31,915	10,391	10,378	14,247	2,469	2,416	1,868
5	45,470	45,459	40,792	44,964	44,955	40,368	6,353	6,346	8,714	3,019	2,961	2,472
6	39,191	39,184	31,835	38,718	38,709	31,494	14,439	14,441	11,775	2,446	2,412	1,731
7	41,760	41,705	36,030	41,370	41,313	35,751	6,789	6,783	8,442	2,655	2,618	2,025
8	40,469	40,418	35,912	39,989	39,936	35,516	6,762	6,751	8,687	2,604	2,548	2,109
9	40,680	40,618	34,833	40,207	40,146	34,491	7,458	7,461	9,211	2,655	2,607	1,944
10	36,569	36,543	31,714	36,196	36,167	31,429	9,790	9,773	13,944	2,473	2,435	1,977
11	41,711	41,693	36,514	41,274	41,254	36,169	9,683	9,685	13,858	2,738	2,696	2,170
12	39,391	39,374	33,661	38,981	38,963	33,363	9,422	9,419	12,644	2,517	2,480	1,936
13	40,341	40,307	34,675	39,956	39,924	34,383	9,456	9,449	13,139	2,655	2,607	2,058
14	46,907	46,848	42,239	46,395	46,337	41,804	11,565	11,557	13,056	3,148	3,072	2,665
15	39,463	39,461	33,967	38,959	38,956	33,582	13,664	13,660	12,008	2,545	2,488	1,866
16	46,164	46,124	40,254	45,860	45,820	40,034	10,704	10,695	12,571	2,961	2,908	2,381
17	48,149	48,116	42,608	47,714	47,682	42,287	9,042	9,043	11,975	3,301	3,244	2,753
18	43,923	43,882	37,775	43,476	43,437	37,444	8,547	8,548	10,636	2,831	2,782	2,206
19	40,755	40,705	34,767	40,366	40,316	34,481	11,346	11,341	13,374	2,752	2,671	2,142
20	40,403	40,345	35,171	40,010	39,954	34,886	8,610	8,612	11,148	2,744	2,661	2,170
21	41,445	41,414	36,250	40,936	40,908	35,863	8,591	8,586	11,229	2,705	2,651	2,152
22	43,356	43,308	37,916	42,896	42,848	37,565	8,536	8,533	10,947	2,819	2,758	2,239
23	42,948	42,865	38,921	42,381	42,299	38,479	10,941	10,950	14,756	2,942	2,857	2,530
24	35,209	35,183	30,324	34,865	34,840	30,073	9,842	9,838	13,450	2,314	2,264	1,780
25	38,052	38,032	32,216	37,656	37,636	31,936	9,548	9,544	12,309	2,445	2,384	1,808
26	39,767	39,753	33,875	39,483	39,469	33,686	10,219	10,216	11,643	2,559	2,516	1,959
27	35,055	35,037	29,681	34,689	34,669	29,412	9,542	9,543	12,107	2,187	2,155	1,643
28	36,529	36,513	31,331	36,111	36,096	31,014	9,326	9,317	12,253	2,398	2,337	1,864
29	39,210	39,181	33,715	38,841	38,819	33,454	13,312	13,309	13,213	2,563	2,488	2,009
30	42,115	42,079	36,805	41,691	41,656	36,472	9,441	9,437	13,166	2,762	2,703	2,176
31	42,912	42,859	38,738	42,427	42,377	38,339	9,539	9,538	15,130	3,043	2,962	2,591
32	42,001	41,972	36,741	41,607	41,577	36,424	9,700	9,689	13,402	2,736	2,684	2,185
33	37,163	37,122	35,613	36,693	36,649	35,183	8,811	8,801	13,521	2,536	2,474	2,100
34	40,158	40,149	36,554	39,666	39,657	36,155	9,176	9,177	15,577	2,822	2,750	2,409
35	36,499	36,476	31,218	36,109	36,086	30,957	8,491	8,480	11,340	2,387	2,334	1,808
36	45,395	45,367	41,464	44,831	44,805	40,995	9,598	9,594	15,411	3,152	3,074	2,756
37	44,153	44,134	40,048	43,617	43,605	39,584	8,496	8,497	13,515	3,018	2,960	2,608
38	43,460	43,419	39,172	43,048	43,005	38,824	8,377	8,378	12,280	2,865	2,791	2,405
39	39,322	39,294	34,622	38,853	38,826	34,253	9,245	9,233	13,308	2,584	2,514	2,070
40	40,821	40,801	36,941	40,350	40,328	36,566	8,149	8,143	12,811	2,819	2,757	2,396
41	44,011	43,986	38,623	43,538	43,515	38,264	6,893	6,892	8,973	2,845	2,789	2,251
42	42,444	42,448	36,315	41,899	41,900	35,880	7,104	7,101	8,561	2,648	2,609	1,968
43	40,018	39,991	34,558	39,586	39,559	34,227	7,686	7,687	9,386	2,602	2,552	1,938
44	43,403	43,362	37,187	42,938	42,893	36,852	9,101	9,098	10,841	2,722	2,673	2,132
45	40,597	40,561	34,749	40,148	40,112	34,411	12,998	12,996	12,765	2,602	2,561	1,995
46	40,880	40,829	35,127	40,454	40,406	34,794	13,375	13,367	12,938	2,595	2,559	1,983
47	39,839	39,824	34,690	39,310	39,298	34,258	11,330	11,337	11,918	2,597	2,544	1,966
48	41,146	41,110	36,046	40,698	40,663	35,692	9,680	9,679	10,781	2,709	2,661	2,164
49	39,773	39,751	34,350	39,409	39,387	34,089	11,174	11,169	10,426	2,580	2,539	1,970
50	41,343	41,284	36,156	40,846	40,784	35,741	10,977	10,972	11,890	2,666	2,614	2,134
51	39,558	39,521	35,230	39,051	39,009	34,826	11,083	11,081	11,430	2,710	2,640	2,186
52	40,435	40,420	35,216	39,962	39,946	34,823	8,956	8,957	11,671	2,640	2,593	2,046
53	39,824	39,790	34,675	39,347	39,315	34,303	6,485	6,481	8,104	2,556	2,500	1,976
54	38,684	38,656	33,922	38,231	38,202	33,563	9,199	9,188	12,230	2,546	2,495	1,997
55	42,315	42,294	37,569	41,751	41,731	37,116	9,377	9,374	13,160	2,741	2,697	2,258
56	36,572	36,556	32,052	36,077	36,064	31,658	8,271	8,265	11,320	2,374	2,327	1,896
57	38,112	38,070	32,833	37,681	37,638	32,509	7,993	7,986	10,181	2,406	2,353	1,829
58	40,398	40,358	35,566	39,941	39,903	35,202	7,591	7,584	9,952	2,637	2,587	2,069
59	37,505	37,481	32,834	37,111	37,088	32,522	8,073	8,068	11,080	2,464	2,406	1,957
60	33,770	33,755	28,257	33,356	33,339	27,969	7,312	7,315	8,873	2,126	2,088	1,492
61	39,642	39,592	34,687	39,126	39,075	34,288	8,416	8,411	11,386	2,611	2,546	2,034
62	34,061	34,029	29,214	33,643	33,614	28,917	6,728	6,723	8,668	2,100	2,050	1,547
63	37,696	37,655	32,123	37,282	37,244	31,821	7,337	7,332	8,538	2,455	2,414	1,802
64	41,848	41,817	37,309	41,389	41,356	36,928	7,395	7,399	10,226	2,785	2,741	2,297
65	44,335	44,284	41,171	43,799	43,746	40,721	14,095	13,876	19,373	3,186	3,127	2,904
**Average**	**40,462**	**40,430**	**35,376**	**40,020**	**39,988**	**35,033**	**9,279**	**9,272**	**11,750**	**2,652**	**2,598**	**2,098**

Raw= no InDel realignment or base quality score recalibration

IR= InDel realignment

IR+BQSR= InDel realignment followed by base quality score recalibration
