# Supplementary material for: Evaluation of variant identification methods for whole genome sequencing data in dairy cattle
Source: BMC Genomics. 2014 Nov 1;15(1):948. doi: 10.1186/1471-2164-15-948 (PMC4289218; doi:10.1186/1471-2164-15-948)
Supplement: Supplementary file 3 — Additional file 3: Concordance with the Illumina BovineSNP50 v1 DNA Analysis BeadChip® (n = 17). a) Non-reference sensitivity (NRS) for single nucleotide variants identified using Platypus (Primitives), Samtools, UnifiedGenotyper and Haplotype Caller (single and multi sample variant identification) using variants identified with the Illumina BovineSNP50 v1 DNA Analysis BeadChip® as a gold standard (BTA1-BTA29). b) Non-reference discrepancy (NRD) for single nucleotide variants identified using Platypus (Primitives), Samtools, UnifiedGenotyper and Haplotype Caller (single and multi sample variant identification) using variants identified with the Illumina BovineSNP50 v1 DNA Analysis BeadChip® as a gold standard (BTA1-BTA29). c) Single nucleotide variant concordance identified using Platypus Primitives), Samtools, UnifiedGenotyper and Haplotype Caller (single and multi sample variant identification) using variants identified with the Illumina BovineSNP50 v1 DNA Analysis BeadChip® as a gold standard (BTA1‒BTA29). d) Single nucleotide variant concordance by genotypes identified using Platypus (Primitives), Samtools, UnifiedGenotyper and Haplotype Caller (single and multi sample variant identification) using variants identified with the Illumina BovineSNP50 v1 DNA Analysis BeadChip® as a gold standard (BTA1‒BTA29). e) Concordance for homozygous reference genotypes identified using Platypus (Primitives), Samtools, UnifiedGenotyper and Haplotype Caller (single and multi sample variant identification) using variants identified with the Illumina BovineSNP50 v1 DNA Analysis BeadChip® as a gold standard (BTA1‒BTA29). f) Concordance for heterozygous genotypes identified using Platypus (Primitives), Samtools, UnifiedGenotyper and Haplotype Caller (single and multi sample variant identification) using variants identified with the Illumina BovineSNP50 v1 DNA Analysis BeadChip® as a gold standard (BTA1‒BTA29). g) Concordance for homozygous alternative genotypes identified using Plat [file 12864_2014_6640_MOESM3_ESM.pdf]

Additional File S3:

Concordance with the Illumina BovineSNP50 v1 DNA Analysis BeadChip® (n=17)

a) Non-reference sensitivity (NRS) for single nucleotide variants identified using Platypus, Samtools, UnifiedGenotyper and Haplotype Caller (single and multi sample variant identification) using variants identified with the Illumina BovineSNP50 v1 DNA Analysis BeadChip® as a gold standard (BTA1-BTA29)

	Single sampe variant identification												Multi sample variant identification				
	Platypus			Platypus (Primitives)			Samtools			UnifiedGenotyper			Haplotype Caller	Platypus	Platypus (Primitives)	Samtools	Unified Genotyper
Animal	***IR+BQSR***	***IR***	***RAW***	**IR+BQSR**	**IR**	**RAW**	**IR+BQSR**	**IR**	**RAW**	**IR+BQSR**	**IR**	**RAW**	**IR+BQSR**	**IR+BQSR**	**IR+BQSR**	**IR+BQSR**	**IR+BQSR**
50K_1	0.779	0.800	0.800	0.806	0.827	0.827	0.846	0.856	0.855	0.813	0.848	0.848	0.914	0.891	0.899	0.906	0.915
50K_2	0.806	0.826	0.826	0.831	0.851	0.851	0.867	0.877	0.877	0.842	0.870	0.870	0.916	0.896	0.904	0.910	0.916
50K_3	0.820	0.835	0.835	0.847	0.863	0.863	0.895	0.903	0.903	0.868	0.891	0.891	0.920	0.894	0.902	0.916	0.922
50K_4	0.798	0.817	0.817	0.826	0.844	0.845	0.877	0.884	0.884	0.843	0.875	0.875	0.921	0.893	0.901	0.914	0.922
50K_5	0.797	0.822	0.822	0.823	0.848	0.848	0.883	0.893	0.893	0.849	0.875	0.875	0.922	0.894	0.902	0.917	0.923
50K_6	0.838	0.860	0.860	0.867	0.888	0.888	0.897	0.907	0.907	0.874	0.901	0.901	0.922	0.900	0.908	0.919	0.923
50K_7	0.820	0.840	0.840	0.846	0.866	0.866	0.878	0.888	0.888	0.857	0.883	0.883	0.920	0.896	0.904	0.913	0.921
50K_8	0.834	0.851	0.851	0.863	0.880	0.880	0.895	0.905	0.905	0.875	0.899	0.900	0.922	0.897	0.905	0.918	0.924
50K_9	0.839	0.849	0.849	0.865	0.875	0.875	0.889	0.893	0.893	0.875	0.888	0.888	0.906	0.879	0.887	0.900	0.906
50K_10	0.790	0.814	0.814	0.816	0.840	0.840	0.867	0.874	0.874	0.834	0.867	0.867	0.917	0.894	0.902	0.910	0.918
50K_11	0.800	0.823	0.823	0.828	0.851	0.851	0.873	0.879	0.879	0.837	0.874	0.874	0.916	0.894	0.901	0.910	0.917
50K_12	0.823	0.837	0.837	0.851	0.865	0.865	0.891	0.896	0.897	0.867	0.888	0.888	0.919	0.895	0.903	0.916	0.920
50K_13	0.814	0.833	0.833	0.840	0.859	0.859	0.883	0.892	0.892	0.859	0.883	0.883	0.919	0.894	0.902	0.911	0.919
50K_14	0.808	0.828	0.828	0.835	0.855	0.855	0.876	0.884	0.884	0.845	0.877	0.877	0.922	0.900	0.909	0.915	0.923
50K_15	0.834	0.850	0.850	0.861	0.876	0.876	0.894	0.900	0.900	0.869	0.892	0.893	0.921	0.899	0.907	0.916	0.923
50K_16	0.765	0.789	0.789	0.792	0.816	0.816	0.846	0.855	0.855	0.802	0.846	0.846	0.902	0.878	0.886	0.897	0.903
50K_17	0.797	0.814	0.813	0.823	0.840	0.840	0.861	0.868	0.868	0.838	0.864	0.864	0.918	0.898	0.907	0.912	0.920
**Average**	**0.810**	**0.829**	**0.829**	**0.836**	**0.856**	**0.856**	**0.877**	**0.886**	**0.886**	**0.850**	**0.878**	**0.878**	**0.918**	**0.894**	**0.902**	**0.912**	**0.919**

b) Non-reference discrepancy (NRD) for single nucleotide variants identified using Platypus, Samtools, UnifiedGenotyper and Haplotype Caller (single and multi sample variant identification) using variants identified with the Illumina BovineSNP50 v1 DNA Analysis BeadChip® as a gold standard (BTA1-BTA29)

	Single sampe variant identification												Multi sample variant identification				
	Platypus			Platypus (Primitives)			Samtools			UnifiedGenotyper			Haplotype Caller	Platypus	Platypus (Primitives)	Samtools	Unified Genotyper
Animal	***IR+BQSR***	***IR***	***RAW***	**IR+BQSR**	**IR**	**RAW**	**IR+BQSR**	**IR**	**RAW**	**IR+BQSR**	**IR**	**RAW**	**IR+BQSR**	**IR+BQSR**	**IR+BQSR**	**IR+BQSR**	**IR+BQSR**
50K_1	0.046	0.045	0.045	0.049	0.048	0.047	0.047	0.046	0.046	0.049	0.048	0.048	0.096	0.090	0.090	0.104	0.095
50K_2	0.047	0.046	0.046	0.048	0.048	0.048	0.047	0.047	0.047	0.048	0.048	0.048	0.092	0.087	0.087	0.098	0.092
50K_3	0.042	0.041	0.041	0.043	0.042	0.042	0.039	0.038	0.038	0.039	0.038	0.039	0.084	0.083	0.083	0.084	0.083
50K_4	0.043	0.043	0.043	0.045	0.045	0.045	0.041	0.040	0.040	0.042	0.042	0.043	0.085	0.083	0.083	0.089	0.084
50K_5	0.045	0.044	0.044	0.047	0.046	0.046	0.042	0.041	0.041	0.043	0.043	0.043	0.082	0.092	0.091	0.084	0.082
50K_6	0.035	0.034	0.034	0.037	0.035	0.035	0.035	0.034	0.034	0.036	0.035	0.035	0.078	0.077	0.077	0.080	0.077
50K_7	0.043	0.041	0.041	0.045	0.043	0.043	0.043	0.042	0.042	0.044	0.043	0.043	0.085	0.085	0.085	0.090	0.085
50K_8	0.040	0.039	0.039	0.041	0.040	0.040	0.040	0.038	0.038	0.041	0.040	0.040	0.081	0.080	0.080	0.081	0.080
50K_9	0.051	0.050	0.050	0.052	0.051	0.051	0.049	0.049	0.049	0.049	0.049	0.050	0.099	0.100	0.101	0.102	0.099
50K_10	0.045	0.044	0.044	0.047	0.045	0.045	0.045	0.044	0.044	0.046	0.045	0.046	0.088	0.085	0.086	0.093	0.087
50K_11	0.047	0.046	0.046	0.048	0.046	0.046	0.047	0.047	0.047	0.048	0.048	0.048	0.090	0.087	0.087	0.092	0.088
50K_12	0.049	0.049	0.049	0.051	0.050	0.050	0.048	0.048	0.048	0.049	0.048	0.049	0.090	0.089	0.090	0.091	0.088
50K_13	0.049	0.048	0.048	0.051	0.050	0.050	0.048	0.048	0.048	0.050	0.049	0.049	0.093	0.092	0.091	0.094	0.093
50K_14	0.046	0.045	0.045	0.047	0.046	0.046	0.045	0.044	0.044	0.046	0.044	0.045	0.081	0.078	0.078	0.090	0.081
50K_15	0.040	0.041	0.041	0.042	0.042	0.042	0.039	0.039	0.039	0.040	0.040	0.040	0.085	0.084	0.084	0.086	0.084
50K_16	0.055	0.054	0.054	0.057	0.056	0.056	0.054	0.052	0.052	0.057	0.054	0.054	0.100	0.099	0.099	0.105	0.099
50K_17	0.046	0.046	0.046	0.048	0.048	0.048	0.045	0.044	0.044	0.047	0.048	0.048	0.088	0.082	0.082	0.098	0.087
**Average**	**0.045**	**0.044**	**0.044**	**0.047**	**0.046**	**0.046**	**0.044**	**0.044**	**0.044**	**0.046**	**0.045**	**0.045**	**0.088**	**0.087**	**0.087**	**0.092**	**0.087**

c) Single nucleotide variant concordance identified using Platypus, Samtools, UnifiedGenotyper and Haplotype Caller (single vs. multi sample variant identification) using variants identified with the Illumina BovineSNP50 v1 DNA Analysis BeadChip® as a gold standard. (BTA1-BTA29)

	Single sampe variant identification												Multi sample variant identification				
	Platypus			Platypus (Primitives)			Samtools			UnifiedGenotyper			Haplotype Caller	Platypus	Platypus (Primitives)	Samtools	Unified Genotyper
Animal	***IR+BQSR***	***IR***	***RAW***	**IR+BQSR**	**IR**	**RAW**	**IR+BQSR**	**IR**	**RAW**	**IR+BQSR**	**IR**	**RAW**	**IR+BQSR**	**IR+BQSR**	**IR+BQSR**	**IR+BQSR**	**IR+BQSR**
50K_1	0.388	0.398	0.398	0.402	0.412	0.412	0.422	0.426	0.426	0.405	0.422	0.422	0.834	0.813	0.821	0.834	0.836
50K_2	0.393	0.402	0.403	0.405	0.415	0.415	0.422	0.427	0.427	0.411	0.424	0.424	0.835	0.815	0.823	0.834	0.838
50K_3	0.396	0.403	0.403	0.409	0.416	0.416	0.432	0.436	0.436	0.419	0.430	0.430	0.837	0.819	0.826	0.834	0.839
50K_4	0.400	0.409	0.409	0.414	0.423	0.423	0.440	0.443	0.443	0.423	0.438	0.438	0.836	0.817	0.825	0.834	0.839
50K_5	0.398	0.411	0.411	0.411	0.424	0.424	0.441	0.446	0.446	0.425	0.437	0.437	0.837	0.818	0.826	0.834	0.839
50K_6	0.414	0.424	0.424	0.428	0.438	0.438	0.443	0.447	0.447	0.432	0.445	0.445	0.837	0.819	0.826	0.834	0.839
50K_7	0.409	0.419	0.419	0.423	0.432	0.432	0.438	0.443	0.443	0.428	0.441	0.441	0.836	0.817	0.825	0.834	0.838
50K_8	0.414	0.422	0.422	0.428	0.437	0.437	0.444	0.449	0.449	0.435	0.446	0.446	0.837	0.819	0.826	0.834	0.839
50K_9	0.403	0.408	0.408	0.416	0.421	0.421	0.428	0.430	0.430	0.421	0.427	0.427	0.835	0.817	0.825	0.833	0.838
50K_10	0.391	0.403	0.403	0.404	0.416	0.416	0.429	0.432	0.432	0.414	0.429	0.429	0.836	0.817	0.825	0.834	0.838
50K_11	0.392	0.403	0.403	0.406	0.417	0.417	0.427	0.430	0.430	0.410	0.428	0.428	0.836	0.817	0.825	0.834	0.838
50K_12	0.409	0.416	0.416	0.423	0.430	0.430	0.442	0.445	0.445	0.431	0.441	0.441	0.836	0.818	0.825	0.834	0.838
50K_13	0.397	0.405	0.405	0.410	0.419	0.419	0.430	0.434	0.434	0.419	0.430	0.430	0.836	0.818	0.826	0.834	0.839
50K_14	0.414	0.424	0.424	0.427	0.438	0.438	0.448	0.452	0.452	0.433	0.449	0.449	0.836	0.816	0.824	0.834	0.838
50K_15	0.434	0.442	0.442	0.449	0.456	0.456	0.465	0.468	0.468	0.452	0.464	0.465	0.837	0.819	0.827	0.834	0.839
50K_16	0.388	0.400	0.400	0.402	0.413	0.413	0.429	0.433	0.433	0.407	0.429	0.429	0.832	0.814	0.821	0.831	0.834
50K_17	0.410	0.418	0.418	0.423	0.432	0.432	0.443	0.446	0.446	0.431	0.444	0.444	0.835	0.814	0.822	0.834	0.837
**Average**	**0.403**	**0.412**	**0.412**	**0.416**	**0.426**	**0.426**	**0.437**	**0.441**	**0.441**	**0.423**	**0.437**	**0.437**	**0.836**	**0.817**	**0.825**	**0.834**	**0.838**

d) Single nucleotide variant concordance by genotypes identified using Platypus, Samtools, UnifiedGenotyper and Haplotype Caller (single vs. multi sample variant identification) using variants identified with the Illumina BovineSNP50 v1 DNA Analysis BeadChip® as a gold standard (BTA1-BTA29)

	**Single sampe variant identification**													**Multi sample variant identification**			
	Platypus			**Platypus (Primitives)**			**Samtools**			**UnifiedGenotyper**			**Haplotype Caller**	**Platypus**	**Platypus (Primitives)**	**Samtools**	**Unified Genotyper**
Animal	***IR+BQSR***	***IR***	***RAW***	**IR+BQSR**	**IR**	**RAW**	**IR+BQSR**	**IR**	**RAW**	**IR+BQSR**	**IR**	**RAW**	**IR+BQSR**	**IR+BQSR**	**IR+BQSR**	**IR+BQSR**	**IR+BQSR**
50K_1	0.954	0.955	0.955	0.951	0.952	0.953	0.953	0.954	0.954	0.951	0.952	0.952	0.945	0.949	0.949	0.940	0.946
50K_2	0.953	0.954	0.954	0.952	0.952	0.952	0.953	0.953	0.953	0.952	0.952	0.952	0.948	0.952	0.952	0.945	0.949
50K_3	0.958	0.959	0.959	0.957	0.958	0.958	0.961	0.962	0.962	0.961	0.962	0.961	0.954	0.955	0.955	0.953	0.954
50K_4	0.957	0.957	0.957	0.955	0.955	0.955	0.959	0.960	0.960	0.958	0.958	0.957	0.951	0.953	0.953	0.948	0.952
50K_5	0.955	0.956	0.956	0.953	0.954	0.954	0.958	0.959	0.959	0.957	0.957	0.957	0.953	0.952	0.952	0.952	0.953
50K_6	0.965	0.966	0.966	0.963	0.965	0.965	0.965	0.966	0.966	0.964	0.965	0.965	0.955	0.957	0.957	0.955	0.957
50K_7	0.957	0.959	0.959	0.955	0.957	0.957	0.957	0.958	0.958	0.956	0.957	0.957	0.951	0.952	0.952	0.948	0.951
50K_8	0.960	0.961	0.961	0.959	0.960	0.960	0.960	0.962	0.962	0.959	0.960	0.960	0.954	0.955	0.955	0.954	0.955
50K_9	0.949	0.950	0.950	0.948	0.949	0.949	0.951	0.951	0.951	0.951	0.951	0.950	0.945	0.946	0.945	0.944	0.946
50K_10	0.955	0.956	0.956	0.953	0.955	0.955	0.955	0.956	0.956	0.954	0.955	0.954	0.950	0.952	0.952	0.947	0.951
50K_11	0.953	0.954	0.954	0.952	0.954	0.954	0.953	0.953	0.953	0.952	0.952	0.952	0.950	0.952	0.952	0.948	0.951
50K_12	0.951	0.951	0.951	0.949	0.950	0.950	0.952	0.952	0.952	0.951	0.952	0.951	0.949	0.950	0.950	0.948	0.950
50K_13	0.951	0.952	0.952	0.949	0.950	0.950	0.952	0.952	0.952	0.950	0.951	0.951	0.948	0.949	0.949	0.947	0.948
50K_14	0.954	0.955	0.955	0.953	0.954	0.954	0.955	0.956	0.956	0.954	0.956	0.955	0.952	0.954	0.954	0.947	0.953
50K_15	0.960	0.959	0.959	0.958	0.958	0.958	0.961	0.961	0.961	0.960	0.960	0.960	0.949	0.950	0.950	0.949	0.950
50K_16	0.945	0.946	0.946	0.943	0.944	0.944	0.946	0.948	0.948	0.943	0.946	0.946	0.942	0.944	0.944	0.939	0.943
50K_17	0.954	0.954	0.954	0.952	0.952	0.952	0.955	0.956	0.956	0.953	0.952	0.952	0.948	0.952	0.952	0.942	0.949
**Average**	**0.955**	**0.956**	**0.956**	**0.953**	**0.954**	**0.954**	**0.956**	**0.956**	**0.956**	**0.954**	**0.955**	**0.955**	**0.950**	**0.951**	**0.951**	**0.947**	**0.950**

e) Concordance for homozygous reference genotypes identified using Platypus, Samtools, UnifiedGenotyper and Haplotype Caller
(single and multi sample variant identification) using variants identified with the Illumina BovineSNP50 v1 DNA Analysis BeadChip® as a gold standard (BTA1-BTA29)

	**Single sampe variant identification**													**Multi sample variant identification**			
	Platypus			**Platypus (Primitives)**			**Samtools**			**UnifiedGenotyper**			**Haplotype Caller**	**Platypus**	**Platypus (Primitives)**	**Samtools**	**Unified Genotyper**
Animal	***IR+BQSR***	***IR***	***RAW***	**IR+BQSR**	**IR**	**RAW**	**IR+BQSR**	**IR**	**RAW**	**IR+BQSR**	**IR**	**RAW**	**IR+BQSR**	**IR+BQSR**	**IR+BQSR**	**IR+BQSR**	**IR+BQSR**
50K_1	0.023	0.023	0.023	0.025	0.025	0.025	0.025	0.025	0.025	0.025	0.025	0.025	0.710	0.697	0.704	0.713	0.713
50K_2	0.024	0.024	0.024	0.025	0.025	0.025	0.025	0.025	0.025	0.026	0.026	0.026	0.719	0.705	0.712	0.718	0.723
50K_3	0.022	0.023	0.023	0.024	0.024	0.024	0.024	0.024	0.024	0.023	0.024	0.024	0.720	0.711	0.718	0.717	0.723
50K_4	0.022	0.022	0.022	0.024	0.024	0.024	0.024	0.024	0.024	0.024	0.024	0.024	0.710	0.703	0.709	0.709	0.714
50K_5	0.025	0.025	0.025	0.026	0.026	0.026	0.026	0.026	0.026	0.026	0.026	0.026	0.714	0.705	0.712	0.711	0.717
50K_6	0.022	0.022	0.022	0.024	0.024	0.024	0.024	0.024	0.024	0.024	0.024	0.024	0.715	0.705	0.712	0.712	0.720
50K_7	0.025	0.025	0.025	0.027	0.027	0.027	0.027	0.027	0.027	0.027	0.027	0.027	0.712	0.702	0.708	0.713	0.716
50K_8	0.025	0.025	0.025	0.026	0.026	0.026	0.026	0.026	0.026	0.026	0.026	0.026	0.714	0.706	0.713	0.713	0.718
50K_9	0.027	0.027	0.027	0.028	0.029	0.029	0.028	0.028	0.028	0.028	0.028	0.028	0.725	0.717	0.724	0.725	0.729
50K_10	0.023	0.023	0.023	0.025	0.025	0.025	0.025	0.025	0.025	0.025	0.026	0.026	0.715	0.704	0.711	0.714	0.719
50K_11	0.025	0.026	0.026	0.026	0.027	0.027	0.027	0.027	0.027	0.026	0.027	0.027	0.716	0.705	0.713	0.716	0.720
50K_12	0.026	0.026	0.026	0.027	0.027	0.028	0.027	0.027	0.027	0.028	0.028	0.028	0.716	0.705	0.712	0.713	0.719
50K_13	0.026	0.026	0.026	0.028	0.028	0.028	0.028	0.028	0.028	0.028	0.028	0.028	0.720	0.709	0.717	0.719	0.723
50K_14	0.026	0.026	0.026	0.027	0.027	0.027	0.027	0.027	0.027	0.027	0.027	0.027	0.706	0.695	0.700	0.706	0.710
50K_15	0.026	0.026	0.026	0.028	0.028	0.028	0.028	0.028	0.028	0.027	0.027	0.028	0.702	0.691	0.698	0.699	0.705
50K_16	0.026	0.026	0.026	0.027	0.027	0.027	0.027	0.027	0.027	0.027	0.027	0.027	0.709	0.700	0.707	0.708	0.712
50K_17	0.025	0.025	0.025	0.027	0.027	0.027	0.027	0.026	0.026	0.026	0.027	0.027	0.704	0.690	0.697	0.703	0.708
**Average**	**0.025**	**0.025**	**0.025**	**0.026**	**0.026**	**0.026**	**0.026**	**0.026**	**0.026**	**0.026**	**0.026**	**0.026**	**0.713**	**0.703**	**0.710**	**0.712**	**0.717**

f) Concordance for heterozygous genotypes identified using Platypus, Samtools, UnifiedGenotyper and Haplotype Caller
(single and multi sample variant identification) using variants identified with the Illumina BovineSNP50 v1 DNA Analysis BeadChip® as a gold standard (BTA1-BTA29)

	**Single sampe variant identification**													**Multi sample variant identification**			
	Platypus			**Platypus (Primitives)**			**Samtools**			**UnifiedGenotyper**			**Haplotype Caller**	**Platypus**	**Platypus (Primitives)**	**Samtools**	**Unified Genotyper**
Animal	***IR+BQSR***	***IR***	***RAW***	**IR+BQSR**	**IR**	**RAW**	**IR+BQSR**	**IR**	**RAW**	**IR+BQSR**	**IR**	**RAW**	**IR+BQSR**	**IR+BQSR**	**IR+BQSR**	**IR+BQSR**	**IR+BQSR**
50K_1	0.810	0.811	0.811	0.839	0.839	0.839	0.851	0.850	0.850	0.851	0.854	0.854	0.981	0.957	0.965	0.971	0.983
50K_2	0.815	0.817	0.817	0.844	0.846	0.846	0.861	0.860	0.860	0.860	0.864	0.864	0.980	0.960	0.967	0.973	0.981
50K_3	0.838	0.838	0.838	0.869	0.868	0.868	0.879	0.877	0.878	0.878	0.878	0.878	0.981	0.958	0.967	0.974	0.983
50K_4	0.821	0.821	0.821	0.851	0.850	0.850	0.865	0.863	0.862	0.864	0.866	0.867	0.982	0.953	0.962	0.974	0.983
50K_5	0.833	0.833	0.833	0.860	0.859	0.859	0.871	0.870	0.871	0.869	0.870	0.870	0.980	0.956	0.966	0.976	0.981
50K_6	0.833	0.834	0.834	0.864	0.864	0.864	0.872	0.870	0.870	0.870	0.872	0.872	0.980	0.959	0.968	0.975	0.981
50K_7	0.832	0.834	0.834	0.861	0.862	0.862	0.871	0.870	0.870	0.869	0.872	0.872	0.980	0.956	0.965	0.972	0.983
50K_8	0.834	0.836	0.836	0.868	0.869	0.869	0.876	0.875	0.875	0.878	0.879	0.879	0.981	0.956	0.966	0.972	0.981
50K_9	0.839	0.841	0.841	0.869	0.871	0.871	0.879	0.877	0.877	0.876	0.879	0.879	0.967	0.947	0.955	0.963	0.968
50K_10	0.819	0.820	0.820	0.846	0.847	0.847	0.863	0.860	0.860	0.865	0.867	0.867	0.978	0.956	0.966	0.974	0.979
50K_11	0.824	0.826	0.826	0.855	0.857	0.857	0.869	0.866	0.866	0.867	0.870	0.870	0.981	0.956	0.964	0.975	0.983
50K_12	0.832	0.832	0.833	0.859	0.860	0.860	0.874	0.874	0.874	0.870	0.873	0.874	0.979	0.954	0.964	0.973	0.980
50K_13	0.830	0.830	0.830	0.859	0.859	0.859	0.871	0.871	0.871	0.873	0.875	0.875	0.981	0.956	0.966	0.974	0.982
50K_14	0.820	0.821	0.821	0.850	0.851	0.851	0.863	0.861	0.861	0.862	0.866	0.866	0.978	0.956	0.967	0.971	0.979
50K_15	0.841	0.841	0.841	0.870	0.870	0.870	0.879	0.877	0.877	0.878	0.880	0.881	0.979	0.957	0.966	0.975	0.981
50K_16	0.825	0.827	0.827	0.852	0.854	0.854	0.864	0.863	0.863	0.866	0.868	0.868	0.968	0.945	0.955	0.967	0.969
50K_17	0.804	0.807	0.807	0.835	0.837	0.837	0.852	0.852	0.852	0.855	0.857	0.857	0.978	0.955	0.963	0.977	0.979
**Average**	**0.827**	**0.827**	**0.828**	**0.856**	**0.857**	**0.857**	**0.868**	**0.867**	**0.867**	**0.868**	**0.870**	**0.870**	**0.979**	**0.955**	**0.964**	**0.973**	**0.980**

g) Concordance for homozygous alternative genotypes identified using Platypus, Samtools, UnifiedGenotyper and Haplotype Caller
(single and multi sample variant identification) using variants identified with the Illumina BovineSNP50 v1 DNA Analysis BeadChip® as a gold standard (BTA1-BTA29)

	**Single sampe variant identification**													**Multi sample variant identification**			
	Platypus			**Platypus (Primitives)**			**Samtools**			**UnifiedGenotyper**			**Haplotype Caller**	**Platypus**	**Platypus (Primitives)**	**Samtools**	**Unified Genotyper**
Animal	***IR+BQSR***	***IR***	***RAW***	**IR+BQSR**	**IR**	**RAW**	**IR+BQSR**	**IR**	**RAW**	**IR+BQSR**	**IR**	**RAW**	**IR+BQSR**	**IR+BQSR**	**IR+BQSR**	**IR+BQSR**	**IR+BQSR**
50K_1	0.810	0.811	0.811	0.851	0.850	0.850	0.851	0.854	0.854	0.981	0.957	0.971	0.956	0.922	0.931	0.957	0.957
50K_2	0.815	0.817	0.817	0.861	0.860	0.860	0.860	0.864	0.864	0.980	0.960	0.973	0.955	0.926	0.935	0.957	0.956
50K_3	0.838	0.838	0.838	0.879	0.877	0.878	0.878	0.878	0.878	0.981	0.958	0.974	0.959	0.927	0.935	0.960	0.959
50K_4	0.821	0.821	0.821	0.865	0.863	0.862	0.864	0.866	0.867	0.982	0.953	0.974	0.957	0.925	0.934	0.958	0.958
50K_5	0.833	0.833	0.833	0.871	0.870	0.871	0.869	0.870	0.870	0.980	0.956	0.976	0.955	0.931	0.939	0.956	0.957
50K_6	0.833	0.834	0.834	0.872	0.870	0.870	0.870	0.872	0.872	0.980	0.959	0.975	0.957	0.930	0.938	0.958	0.957
50K_7	0.832	0.834	0.834	0.871	0.870	0.870	0.869	0.872	0.872	0.980	0.956	0.972	0.958	0.930	0.938	0.957	0.958
50K_8	0.834	0.836	0.836	0.876	0.875	0.875	0.878	0.879	0.879	0.981	0.956	0.972	0.956	0.927	0.934	0.958	0.958
50K_9	0.839	0.841	0.841	0.879	0.877	0.877	0.876	0.879	0.879	0.967	0.947	0.963	0.960	0.926	0.935	0.957	0.960
50K_10	0.819	0.820	0.820	0.863	0.860	0.860	0.865	0.867	0.867	0.978	0.956	0.974	0.956	0.929	0.936	0.954	0.958
50K_11	0.824	0.826	0.826	0.869	0.866	0.866	0.867	0.870	0.870	0.981	0.956	0.975	0.956	0.929	0.937	0.955	0.957
50K_12	0.832	0.832	0.833	0.874	0.874	0.874	0.870	0.873	0.874	0.979	0.954	0.973	0.956	0.931	0.939	0.960	0.958
50K_13	0.830	0.830	0.830	0.871	0.871	0.871	0.873	0.875	0.875	0.981	0.956	0.974	0.958	0.932	0.938	0.956	0.959
50K_14	0.820	0.821	0.821	0.863	0.861	0.861	0.862	0.866	0.866	0.978	0.956	0.971	0.958	0.926	0.935	0.959	0.959
50K_15	0.841	0.841	0.841	0.879	0.877	0.877	0.878	0.880	0.881	0.979	0.957	0.975	0.958	0.932	0.940	0.957	0.959
50K_16	0.825	0.827	0.827	0.864	0.863	0.863	0.866	0.868	0.868	0.968	0.945	0.967	0.955	0.925	0.932	0.952	0.956
50K_17	0.804	0.807	0.807	0.852	0.852	0.852	0.855	0.857	0.857	0.978	0.955	0.977	0.958	0.926	0.936	0.954	0.959
**Average**	**0.827**	**0.827**	**0.828**	**0.868**	**0.867**	**0.867**	**0.868**	**0.870**	**0.870**	**0.979**	**0.955**	**0.973**	**0.957**	**0.928**	**0.936**	**0.957**	**0.958**

Raw= no InDel realignment or base quality score recalibration
IR= InDel realignment
IR+BQSR= InDel realignment followed by base quality score recalibration
