# Supplementary material for: Evaluation of variant identification methods for whole genome sequencing data in dairy cattle
Source: BMC Genomics. 2014 Nov 1;15(1):948. doi: 10.1186/1471-2164-15-948 (PMC4289218; doi:10.1186/1471-2164-15-948)
Supplement: Supplementary file 4 — Additional file 4: Concordance with the Illumina Concordance with the Illumina BovineHD BeadChip® (n = 48). a) Non-reference sensitivity (NRS) for single nucleotide variants identified using Platypus (Primitives), Samtools, UnifiedGenotyper and Haplotype Caller (single and multi sample variant identification) using variants identified with the Illumina BovineHD BeadChip® as a gold standard (BTA1-BTA29). b) Non-reference discrepancy (NRD) for single nucleotide variants identified using Platypus (Primitives), Samtools, UnifiedGenotyper and Haplotype Caller (single and multi sample variant identification) using variants identified with the Illumina BovineHD BeadChip® as a gold standard (BTA1-BTA29). c) Single nucleotide variant concordance identified using Platypus Primitives), Samtools, UnifiedGenotyper and Haplotype Caller (single and multi sample variant identification) using variants identified with the Illumina BovineHD BeadChip® as a gold standard (BTA1-BTA29). d) Single nucleotide variant concordance by genotypes identified using Platypus (Primitives), Samtools, UnifiedGenotyper and Haplotype Caller (single and multi sample variant identification) using variants identified with the Illumina BovineHD BeadChip® as a gold standard (BTA1-BTA29). e) Concordance for homozygous reference genotypes identified using Platypus (Primitives), Samtools, UnifiedGenotyper and Haplotype Caller (single and multi sample variant identification) using variants identified with the Illumina BovineHD BeadChip® as a gold standard (BTA1-BTA29). f) Concordance for heterozygous genotypes identified using Platypus (Primitives), Samtools, UnifiedGenotyper and Haplotype Caller (single and multi sample variant identification) using variants identified with the Illumina BovineHD BeadChip® as a gold standard (BTA1-BTA29). g) Concordance for homozygous alternative genotypes identified using Platypus (Primitives), Samtools, UnifiedGenotyper and Haplotype Caller (single and multi sample variant ide [file 12864_2014_6640_MOESM4_ESM.pdf]

Additional File S4:

Concordance with the Illumina BovineHD BeadChip® (n=48)

a) Non-reference sensitivity (NRS) for single nucleotide variants identified using Platypus, Samtools, UnifiedGenotyper and Haplotype Caller (single and multi sample variant identification) using variants identified with the Illumina BovineHD BeadChip® as a gold standard (BTA1-BTA29)

	**Single sampe variant identification**													**Multi sample variant identification**			
	Platypus			**Platypus (Primitives)**			**Samtools**			**UnifiedGenotyper**			**Haplotype Caller**	**Platypus**	**Platypus (Primitives)**	**Samtools**	**Unified Genotyper**
Animal	***IR+BQSR***	***IR***	***RAW***	**IR+BQSR**	**IR**	**RAW**	**IR+BQSR**	**IR**	**RAW**	**IR+BQSR**	**IR**	**RAW**	**IR+BQSR**	**IR+BQSR**	**IR+BQSR**	**IR+BQSR**	**IR+BQSR**
HD_1	*0.909*	*0.914*	*0.914*	0.919	0.978	0.978	0.972	0.973	0.983	0.979	0.985	0.985	0.981	0.934	0.949	0.974	0.981
HD_2	*0.852*	*0.866*	*0.866*	0.911	0.925	0.925	0.945	0.948	0.948	0.920	0.945	0.945	0.976	0.930	0.950	0.974	0.976
HD_3	*0.853*	*0.869*	*0.869*	0.912	0.928	0.928	0.947	0.952	0.952	0.921	0.948	0.948	0.977	0.930	0.950	0.975	0.977
HD_4	*0.844*	*0.859*	*0.859*	0.902	0.917	0.917	0.939	0.943	0.943	0.913	0.938	0.938	0.975	0.929	0.949	0.974	0.975
HD_5	*0.840*	*0.859*	*0.859*	0.898	0.916	0.916	0.947	0.953	0.953	0.921	0.945	0.945	0.977	0.930	0.950	0.975	0.977
HD_6	*0.863*	*0.876*	*0.876*	0.922	0.936	0.936	0.948	0.952	0.952	0.932	0.952	0.952	0.977	0.931	0.950	0.975	0.977
HD_7	*0.817*	*0.842*	*0.842*	0.870	0.897	0.897	0.916	0.925	0.925	0.883	0.922	0.922	0.969	0.925	0.945	0.968	0.969
HD_8	*0.860*	*0.877*	*0.877*	0.919	0.937	0.937	0.951	0.956	0.956	0.928	0.953	0.953	0.977	0.930	0.950	0.975	0.977
HD_9	*0.860*	*0.875*	*0.875*	0.919	0.935	0.935	0.957	0.961	0.961	0.937	0.955	0.955	0.978	0.931	0.951	0.976	0.978
HD_10	*0.885*	*0.898*	*0.898*	0.947	0.959	0.959	0.969	0.972	0.972	0.959	0.971	0.972	0.980	0.932	0.952	0.978	0.980
HD_11	*0.856*	*0.874*	*0.874*	0.915	0.934	0.934	0.951	0.956	0.956	0.927	0.952	0.952	0.977	0.931	0.950	0.976	0.977
HD_12	*0.880*	*0.894*	*0.894*	0.941	0.955	0.955	0.965	0.969	0.969	0.953	0.968	0.968	0.978	0.932	0.952	0.978	0.979
HD_13	*0.868*	*0.886*	*0.886*	0.928	0.946	0.946	0.957	0.962	0.962	0.940	0.961	0.961	0.978	0.932	0.951	0.977	0.978
HD_14	*0.876*	*0.890*	*0.890*	0.936	0.951	0.951	0.962	0.966	0.966	0.947	0.965	0.965	0.979	0.932	0.951	0.978	0.979
HD_15	*0.877*	*0.892*	*0.892*	0.938	0.953	0.953	0.963	0.967	0.967	0.949	0.966	0.966	0.980	0.932	0.952	0.978	0.980
HD_16	*0.860*	*0.876*	*0.876*	0.919	0.935	0.935	0.954	0.959	0.959	0.934	0.954	0.954	0.978	0.931	0.951	0.977	0.979
HD_17	*0.843*	*0.863*	*0.863*	0.901	0.921	0.921	0.944	0.950	0.950	0.916	0.944	0.944	0.976	0.930	0.949	0.975	0.976
HD_18	*0.843*	*0.861*	*0.861*	0.900	0.919	0.919	0.942	0.947	0.947	0.915	0.943	0.943	0.977	0.929	0.949	0.975	0.977
HD_19	*0.845*	*0.863*	*0.863*	0.903	0.922	0.922	0.943	0.948	0.948	0.917	0.944	0.944	0.976	0.930	0.949	0.975	0.976
HD_20	*0.866*	*0.880*	*0.880*	0.925	0.940	0.940	0.958	0.963	0.963	0.940	0.959	0.959	0.978	0.931	0.951	0.977	0.979
HD_21	*0.888*	*0.893*	*0.893*	0.949	0.954	0.954	0.967	0.970	0.971	0.955	0.968	0.968	0.980	0.933	0.952	0.979	0.980
HD_22	*0.886*	*0.895*	*0.895*	0.947	0.956	0.956	0.970	0.972	0.972	0.960	0.971	0.971	0.980	0.933	0.952	0.979	0.980
HD_23	*0.840*	*0.859*	*0.859*	0.897	0.916	0.916	0.940	0.945	0.945	0.911	0.940	0.940	0.975	0.930	0.949	0.974	0.976
HD_24	*0.883*	*0.892*	*0.892*	0.942	0.952	0.952	0.967	0.971	0.971	0.956	0.968	0.968	0.978	0.932	0.951	0.977	0.979
HD_25	*0.883*	*0.893*	*0.893*	0.944	0.954	0.954	0.968	0.972	0.972	0.957	0.969	0.969	0.980	0.933	0.952	0.979	0.981
HD_26	*0.882*	*0.893*	*0.893*	0.943	0.955	0.955	0.967	0.971	0.971	0.954	0.968	0.968	0.979	0.933	0.952	0.978	0.979
HD_27	*0.856*	*0.871*	*0.871*	0.914	0.929	0.929	0.950	0.956	0.956	0.927	0.949	0.949	0.975	0.929	0.948	0.974	0.975
HD_28	*0.882*	*0.892*	*0.892*	0.942	0.952	0.952	0.968	0.971	0.971	0.955	0.967	0.967	0.980	0.932	0.952	0.978	0.980
HD_29	*0.847*	*0.863*	*0.863*	0.904	0.920	0.920	0.941	0.946	0.946	0.915	0.942	0.942	0.973	0.929	0.948	0.972	0.974
HD_30	*0.854*	*0.872*	*0.872*	0.913	0.931	0.931	0.949	0.954	0.954	0.923	0.950	0.951	0.978	0.931	0.950	0.975	0.978
HD_31	*0.864*	*0.880*	*0.880*	0.923	0.940	0.940	0.955	0.959	0.959	0.934	0.957	0.957	0.978	0.931	0.951	0.977	0.979
HD_32	*0.862*	*0.879*	*0.879*	0.921	0.939	0.939	0.953	0.959	0.959	0.933	0.956	0.956	0.979	0.932	0.952	0.977	0.979
HD_33	*0.860*	*0.879*	*0.879*	0.920	0.939	0.939	0.954	0.959	0.959	0.932	0.956	0.956	0.978	0.931	0.951	0.976	0.978
HD_34	*0.859*	*0.878*	*0.878*	0.917	0.937	0.937	0.952	0.958	0.958	0.930	0.954	0.954	0.975	0.930	0.949	0.975	0.976
HD_35	*0.862*	*0.879*	*0.879*	0.921	0.939	0.939	0.953	0.958	0.958	0.933	0.955	0.956	0.978	0.931	0.950	0.976	0.978
HD_36	*0.862*	*0.878*	*0.878*	0.922	0.938	0.938	0.953	0.957	0.957	0.934	0.955	0.955	0.978	0.931	0.951	0.977	0.978
HD_37	*0.855*	*0.873*	*0.873*	0.914	0.932	0.932	0.950	0.954	0.954	0.926	0.951	0.951	0.978	0.931	0.951	0.976	0.978
HD_38	*0.870*	*0.886*	*0.886*	0.930	0.946	0.946	0.959	0.963	0.963	0.942	0.961	0.961	0.978	0.932	0.951	0.977	0.979
HD_39	*0.877*	*0.890*	*0.890*	0.936	0.949	0.949	0.962	0.967	0.967	0.947	0.963	0.963	0.977	0.932	0.951	0.976	0.978
HD_40	*0.859*	*0.876*	*0.876*	0.919	0.936	0.936	0.954	0.959	0.959	0.933	0.954	0.954	0.978	0.932	0.951	0.976	0.979
HD_41	*0.868*	*0.883*	*0.883*	0.927	0.943	0.943	0.959	0.964	0.964	0.940	0.959	0.959	0.978	0.932	0.952	0.977	0.979
HD_42	*0.872*	*0.885*	*0.885*	0.933	0.946	0.946	0.962	0.966	0.966	0.946	0.962	0.962	0.978	0.932	0.952	0.977	0.979
HD_43	*0.856*	*0.871*	*0.871*	0.915	0.930	0.930	0.951	0.955	0.955	0.929	0.950	0.950	0.976	0.930	0.950	0.975	0.977
HD_44	*0.858*	*0.876*	*0.876*	0.918	0.935	0.935	0.952	0.957	0.957	0.928	0.952	0.952	0.977	0.931	0.950	0.976	0.977
HD_45	*0.872*	*0.886*	*0.886*	0.932	0.946	0.946	0.960	0.964	0.964	0.942	0.961	0.961	0.978	0.932	0.952	0.977	0.979
HD_46	*0.859*	*0.875*	*0.875*	0.919	0.935	0.935	0.951	0.956	0.955	0.930	0.951	0.952	0.977	0.930	0.950	0.975	0.977
HD_47	*0.831*	*0.848*	*0.848*	0.889	0.906	0.906	0.930	0.934	0.934	0.899	0.929	0.929	0.973	0.928	0.948	0.972	0.973
HD_48	*0.841*	*0.860*	*0.860*	0.899	0.919	0.919	0.938	0.942	0.942	0.909	0.941	0.941	0.975	0.928	0.948	0.973	0.975
**Average**	**0.862**	**0.877**	**0.878**	**0.920**	**0.937**	**0.937**	**0.953**	**0.958**	**0.958**	**0.934**	**0.955**	**0.955**	**0.977**	**0.931**	**0.950**	**0.976**	**0.978**

b) Non-reference discrepancy (NRD) for single nucleotide variants identified using Platypus, Samtools, UnifiedGenotyper and Haplotype Caller (single and multi sample variant identification) using variants identified with the Illumina BovineHD BeadChip® as a gold standard (BTA1-BTA29)

	**Single sampe variant identification**													**Multi sample variant identification**				
	Platypus			**Platypus (Primitives)**			**Samtools**			**UnifiedGenotyper**			**Haplotype Caller**	**Platypus**	**Platypus (Primitives)**	**Samtools**	**Unified Genotyper**
Animal	***IR+BQSR***	***IR***	***RAW***	**IR+BQSR**	**IR**	**RAW**	**IR+BQSR**	**IR**	**RAW**	**IR+BQSR**	**IR**	**RAW**	**IR+BQSR**	**IR+BQSR**	**IR+BQSR**	**IR+BQSR**	**IR+BQSR**
HD_1	0.003	0.003	0.003	0.008	0.004	0.004	0.003	0.003	0.003	0.004	0.004	0.004	0.007	0.005	0.007	0.023	0.006
HD_2	0.008	0.007	0.007	0.009	0.008	0.008	0.008	0.007	0.007	0.008	0.008	0.008	0.012	0.009	0.010	0.013	0.011
HD_3	0.007	0.007	0.007	0.008	0.008	0.008	0.007	0.007	0.007	0.008	0.007	0.007	0.011	0.009	0.010	0.012	0.011
HD_4	0.008	0.008	0.008	0.009	0.009	0.009	0.008	0.008	0.008	0.008	0.008	0.008	0.013	0.009	0.010	0.014	0.012
HD_5	0.010	0.009	0.009	0.011	0.010	0.010	0.008	0.008	0.008	0.008	0.008	0.008	0.012	0.009	0.010	0.011	0.011
HD_6	0.007	0.007	0.007	0.008	0.008	0.008	0.008	0.008	0.008	0.008	0.008	0.008	0.012	0.009	0.010	0.013	0.012
HD_7	0.019	0.019	0.019	0.021	0.021	0.021	0.021	0.019	0.019	0.021	0.021	0.021	0.026	0.020	0.021	0.028	0.025
HD_8	0.008	0.008	0.008	0.009	0.009	0.009	0.008	0.008	0.008	0.009	0.009	0.009	0.013	0.010	0.010	0.012	0.012
HD_9	0.007	0.007	0.007	0.008	0.008	0.008	0.006	0.006	0.006	0.006	0.006	0.006	0.010	0.008	0.008	0.009	0.009
HD_10	0.006	0.007	0.007	0.007	0.008	0.008	0.006	0.006	0.007	0.006	0.007	0.007	0.010	0.008	0.009	0.009	0.009
HD_11	0.007	0.007	0.007	0.008	0.008	0.007	0.007	0.006	0.006	0.007	0.007	0.007	0.011	0.008	0.009	0.011	0.010
HD_12	0.005	0.004	0.004	0.006	0.005	0.005	0.005	0.004	0.004	0.005	0.005	0.005	0.008	0.006	0.007	0.008	0.008
HD_13	0.006	0.005	0.005	0.007	0.006	0.006	0.006	0.005	0.005	0.006	0.006	0.006	0.010	0.007	0.008	0.010	0.009
HD_14	0.005	0.005	0.005	0.006	0.006	0.006	0.006	0.005	0.005	0.006	0.006	0.006	0.009	0.007	0.008	0.008	0.008
HD_15	0.005	0.004	0.004	0.006	0.005	0.005	0.005	0.005	0.005	0.005	0.005	0.005	0.008	0.007	0.007	0.008	0.008
HD_16	0.007	0.007	0.007	0.008	0.008	0.008	0.007	0.006	0.006	0.007	0.007	0.007	0.011	0.008	0.009	0.011	0.010
HD_17	0.008	0.008	0.008	0.009	0.009	0.009	0.008	0.007	0.007	0.008	0.008	0.008	0.012	0.010	0.010	0.013	0.011
HD_18	0.008	0.008	0.008	0.009	0.009	0.009	0.008	0.008	0.008	0.009	0.009	0.009	0.012	0.010	0.011	0.013	0.012
HD_19	0.008	0.008	0.008	0.009	0.009	0.009	0.009	0.008	0.008	0.009	0.009	0.009	0.012	0.009	0.010	0.013	0.012
HD_20	0.007	0.007	0.007	0.008	0.008	0.008	0.007	0.006	0.006	0.007	0.007	0.007	0.011	0.008	0.009	0.010	0.010
HD_21	0.004	0.005	0.005	0.005	0.006	0.006	0.005	0.005	0.005	0.005	0.005	0.006	0.009	0.007	0.008	0.009	0.008
HD_22	0.005	0.006	0.006	0.006	0.007	0.007	0.005	0.005	0.005	0.006	0.006	0.006	0.010	0.007	0.008	0.008	0.008
HD_23	0.009	0.009	0.009	0.010	0.010	0.010	0.009	0.008	0.008	0.009	0.009	0.009	0.013	0.010	0.011	0.014	0.013
HD_24	0.012	0.012	0.012	0.013	0.013	0.013	0.012	0.011	0.011	0.012	0.012	0.013	0.015	0.013	0.014	0.014	0.014
HD_25	0.005	0.005	0.005	0.006	0.006	0.006	0.005	0.005	0.005	0.005	0.005	0.006	0.009	0.007	0.008	0.008	0.008
HD_26	0.005	0.005	0.005	0.006	0.006	0.006	0.006	0.005	0.005	0.006	0.006	0.006	0.009	0.007	0.008	0.008	0.008
HD_27	0.015	0.014	0.014	0.016	0.016	0.016	0.015	0.014	0.014	0.015	0.015	0.015	0.018	0.015	0.016	0.018	0.017
HD_28	0.005	0.005	0.005	0.006	0.005	0.005	0.005	0.004	0.004	0.005	0.005	0.005	0.009	0.007	0.007	0.008	0.008
HD_29	0.016	0.016	0.016	0.018	0.017	0.017	0.017	0.016	0.015	0.017	0.017	0.017	0.020	0.017	0.017	0.021	0.019
HD_30	0.007	0.007	0.007	0.009	0.008	0.008	0.008	0.007	0.007	0.008	0.007	0.007	0.011	0.009	0.009	0.011	0.010
HD_31	0.006	0.006	0.006	0.007	0.007	0.007	0.007	0.006	0.006	0.007	0.007	0.007	0.011	0.008	0.009	0.011	0.010
HD_32	0.007	0.007	0.007	0.008	0.008	0.008	0.007	0.007	0.007	0.007	0.008	0.008	0.012	0.008	0.009	0.011	0.010
HD_33	0.007	0.007	0.007	0.009	0.009	0.009	0.008	0.008	0.008	0.008	0.008	0.008	0.013	0.010	0.010	0.012	0.011
HD_34	0.014	0.014	0.014	0.015	0.015	0.015	0.014	0.013	0.013	0.014	0.014	0.014	0.017	0.015	0.015	0.017	0.017
HD_35	0.006	0.006	0.006	0.007	0.007	0.007	0.007	0.006	0.006	0.007	0.007	0.007	0.011	0.009	0.009	0.011	0.010
HD_36	0.007	0.006	0.006	0.007	0.007	0.007	0.007	0.006	0.006	0.007	0.007	0.007	0.011	0.008	0.009	0.011	0.010
HD_37	0.009	0.010	0.010	0.010	0.011	0.011	0.009	0.009	0.009	0.009	0.010	0.011	0.015	0.011	0.012	0.014	0.013
HD_38	0.007	0.006	0.006	0.008	0.007	0.007	0.007	0.006	0.006	0.007	0.007	0.007	0.010	0.008	0.008	0.010	0.009
HD_39	0.012	0.011	0.011	0.013	0.013	0.013	0.012	0.011	0.011	0.012	0.012	0.012	0.015	0.013	0.014	0.015	0.014
HD_40	0.007	0.006	0.006	0.008	0.007	0.007	0.007	0.006	0.006	0.007	0.007	0.007	0.010	0.008	0.009	0.010	0.010
HD_41	0.006	0.006	0.006	0.007	0.007	0.007	0.006	0.006	0.006	0.006	0.006	0.006	0.010	0.007	0.008	0.009	0.008
HD_42	0.006	0.006	0.006	0.007	0.007	0.007	0.006	0.005	0.005	0.006	0.006	0.006	0.010	0.007	0.008	0.009	0.008
HD_43	0.007	0.007	0.007	0.008	0.008	0.008	0.007	0.007	0.007	0.008	0.007	0.008	0.012	0.009	0.010	0.012	0.011
HD_44	0.007	0.007	0.007	0.008	0.008	0.008	0.007	0.007	0.007	0.007	0.007	0.008	0.011	0.009	0.009	0.011	0.010
HD_45	0.006	0.006	0.006	0.007	0.007	0.007	0.006	0.006	0.006	0.006	0.007	0.007	0.011	0.008	0.009	0.010	0.009
HD_46	0.008	0.008	0.008	0.009	0.009	0.009	0.008	0.008	0.008	0.008	0.009	0.009	0.013	0.010	0.010	0.013	0.012
HD_47	0.011	0.011	0.011	0.012	0.012	0.012	0.011	0.011	0.011	0.011	0.012	0.012	0.017	0.013	0.014	0.021	0.016
HD_48	0.009	0.008	0.008	0.010	0.009	0.009	0.009	0.008	0.008	0.009	0.009	0.009	0.013	0.011	0.011	0.014	0.012
**Average**	**0.008**	**0.008**	**0.008**	**0.009**	**0.009**	**0.009**	**0.008**	**0.007**	**0.007**	**0.008**	**0.008**	**0.008**	**0.012**	**0.009**	**0.010**	**0.012**	**0.011**

c) Single nucleotide variant concordance identified using Platypus, Samtools, UnifiedGenotyper and Haplotype Caller (single vs. multi sample variant identification) using variants identified with the Illumina BovineHD BeadChip® as a gold standard. (BTA1-BTA29)

	**Single sampe variant identification**												**Multi sample variant identification**				
	Platypus			**Platypus (Primitives)**			**Samtools**			**UnifiedGenotyper**			**Haplotype Caller**	**Platypus**	**Platypus (Primitives)**	**Samtools**	**Unified Genotyper**
Animal	***IR+BQSR***	***IR***	***RAW***	**IR+BQSR**	**IR**	**RAW**	**IR+BQSR**	**IR**	**RAW**	**IR+BQSR**	**IR**	**RAW**	**IR+BQSR**	**IR+BQSR**	**IR+BQSR**	**IR+BQSR**	**IR+BQSR**
HD_1	0.552	0.555	0.555	0.572	0.593	0.593	0.592	0.593	0.597	0.595	0.598	0.598	0.881	0.839	0.857	0.879	0.882
HD_2	0.542	0.552	0.552	0.580	0.589	0.589	0.602	0.604	0.604	0.587	0.602	0.602	0.881	0.838	0.857	0.879	0.882
HD_3	0.529	0.539	0.539	0.565	0.575	0.575	0.588	0.590	0.590	0.571	0.588	0.588	0.881	0.838	0.858	0.879	0.882
HD_4	0.535	0.545	0.545	0.572	0.581	0.581	0.596	0.599	0.599	0.580	0.596	0.596	0.880	0.837	0.857	0.879	0.882
HD_5	0.536	0.548	0.548	0.572	0.584	0.584	0.605	0.609	0.609	0.588	0.603	0.603	0.881	0.838	0.858	0.879	0.882
HD_6	0.550	0.559	0.559	0.588	0.597	0.597	0.606	0.608	0.608	0.595	0.608	0.608	0.881	0.838	0.857	0.879	0.882
HD_7	0.517	0.533	0.533	0.551	0.567	0.567	0.580	0.585	0.585	0.559	0.584	0.584	0.878	0.834	0.853	0.877	0.879
HD_8	0.542	0.554	0.554	0.580	0.591	0.591	0.600	0.603	0.603	0.586	0.602	0.602	0.881	0.838	0.858	0.879	0.882
HD_9	0.551	0.561	0.561	0.589	0.599	0.599	0.614	0.617	0.617	0.601	0.613	0.613	0.881	0.839	0.858	0.879	0.882
HD_10	0.560	0.568	0.568	0.599	0.607	0.607	0.614	0.616	0.616	0.607	0.615	0.615	0.881	0.839	0.858	0.879	0.882
HD_11	0.538	0.549	0.549	0.575	0.586	0.586	0.598	0.601	0.601	0.583	0.599	0.599	0.881	0.839	0.858	0.879	0.882
HD_12	0.564	0.573	0.573	0.602	0.611	0.611	0.618	0.621	0.621	0.611	0.621	0.621	0.881	0.839	0.858	0.879	0.882
HD_13	0.541	0.552	0.552	0.578	0.589	0.589	0.597	0.600	0.600	0.587	0.600	0.600	0.881	0.838	0.858	0.879	0.882
HD_14	0.543	0.553	0.553	0.581	0.590	0.590	0.597	0.600	0.600	0.588	0.599	0.599	0.881	0.839	0.858	0.879	0.882
HD_15	0.542	0.551	0.551	0.579	0.588	0.588	0.596	0.598	0.598	0.587	0.597	0.597	0.881	0.839	0.858	0.879	0.882
HD_16	0.528	0.538	0.538	0.564	0.574	0.574	0.586	0.589	0.589	0.574	0.587	0.587	0.881	0.838	0.858	0.879	0.882
HD_17	0.528	0.541	0.541	0.565	0.577	0.577	0.592	0.596	0.596	0.575	0.592	0.593	0.881	0.838	0.858	0.879	0.882
HD_18	0.515	0.527	0.527	0.551	0.562	0.562	0.577	0.580	0.580	0.560	0.578	0.578	0.881	0.838	0.857	0.879	0.882
HD_19	0.525	0.536	0.536	0.560	0.572	0.572	0.586	0.589	0.589	0.570	0.587	0.587	0.881	0.838	0.857	0.879	0.882
HD_20	0.548	0.557	0.557	0.585	0.595	0.595	0.607	0.610	0.610	0.595	0.607	0.607	0.881	0.838	0.858	0.879	0.882
HD_21	0.535	0.538	0.538	0.572	0.575	0.575	0.584	0.585	0.585	0.576	0.584	0.584	0.881	0.839	0.858	0.879	0.882
HD_22	0.541	0.547	0.547	0.578	0.584	0.584	0.593	0.595	0.595	0.587	0.594	0.594	0.881	0.839	0.858	0.879	0.882
HD_23	0.526	0.537	0.537	0.562	0.573	0.573	0.589	0.592	0.592	0.571	0.589	0.589	0.881	0.838	0.857	0.879	0.882
HD_24	0.552	0.557	0.557	0.589	0.594	0.594	0.605	0.607	0.607	0.598	0.605	0.605	0.879	0.838	0.857	0.878	0.880
HD_25	0.552	0.558	0.558	0.590	0.596	0.596	0.606	0.608	0.608	0.599	0.607	0.607	0.881	0.839	0.858	0.879	0.882
HD_26	0.561	0.569	0.569	0.600	0.607	0.607	0.616	0.618	0.618	0.608	0.616	0.617	0.881	0.839	0.858	0.879	0.882
HD_27	0.534	0.544	0.544	0.570	0.579	0.579	0.594	0.596	0.596	0.579	0.593	0.593	0.879	0.837	0.856	0.878	0.880
HD_28	0.551	0.558	0.558	0.589	0.595	0.595	0.606	0.608	0.608	0.598	0.606	0.606	0.881	0.839	0.858	0.879	0.882
HD_29	0.546	0.556	0.556	0.583	0.593	0.593	0.607	0.610	0.610	0.590	0.608	0.608	0.879	0.836	0.855	0.877	0.880
HD_30	0.552	0.564	0.564	0.590	0.602	0.601	0.614	0.617	0.617	0.597	0.615	0.615	0.881	0.838	0.858	0.879	0.882
HD_31	0.540	0.550	0.550	0.577	0.587	0.587	0.597	0.600	0.600	0.584	0.598	0.598	0.881	0.839	0.858	0.879	0.882
HD_32	0.555	0.566	0.566	0.593	0.605	0.605	0.615	0.618	0.618	0.601	0.617	0.617	0.881	0.838	0.858	0.879	0.882
HD_33	0.551	0.563	0.563	0.589	0.601	0.601	0.611	0.614	0.614	0.597	0.613	0.613	0.881	0.838	0.858	0.879	0.882
HD_34	0.548	0.560	0.560	0.585	0.597	0.597	0.607	0.611	0.611	0.593	0.609	0.609	0.879	0.837	0.856	0.878	0.880
HD_35	0.539	0.550	0.550	0.576	0.587	0.587	0.597	0.600	0.600	0.585	0.599	0.599	0.881	0.838	0.858	0.879	0.882
HD_36	0.540	0.550	0.550	0.577	0.587	0.587	0.598	0.600	0.600	0.586	0.599	0.599	0.881	0.838	0.858	0.879	0.882
HD_37	0.548	0.559	0.559	0.585	0.597	0.597	0.609	0.612	0.612	0.594	0.610	0.610	0.881	0.838	0.858	0.879	0.882
HD_38	0.560	0.570	0.570	0.599	0.609	0.609	0.618	0.621	0.621	0.607	0.619	0.619	0.881	0.839	0.858	0.879	0.883
HD_39	0.544	0.552	0.552	0.580	0.588	0.588	0.597	0.600	0.600	0.588	0.598	0.598	0.879	0.838	0.857	0.878	0.880
HD_40	0.552	0.563	0.563	0.590	0.600	0.600	0.613	0.616	0.616	0.599	0.613	0.613	0.881	0.838	0.858	0.879	0.882
HD_41	0.554	0.564	0.564	0.592	0.602	0.602	0.613	0.616	0.616	0.601	0.613	0.613	0.881	0.839	0.858	0.879	0.882
HD_42	0.556	0.564	0.564	0.594	0.602	0.602	0.614	0.616	0.616	0.604	0.614	0.614	0.881	0.839	0.858	0.879	0.882
HD_43	0.540	0.549	0.549	0.576	0.586	0.586	0.600	0.602	0.602	0.586	0.599	0.600	0.881	0.838	0.858	0.879	0.882
HD_44	0.548	0.560	0.560	0.586	0.597	0.597	0.609	0.612	0.612	0.593	0.609	0.609	0.881	0.839	0.858	0.879	0.882
HD_45	0.532	0.541	0.541	0.569	0.577	0.577	0.586	0.589	0.589	0.576	0.587	0.587	0.881	0.839	0.858	0.879	0.882
HD_46	0.538	0.548	0.548	0.575	0.585	0.585	0.596	0.599	0.599	0.583	0.596	0.596	0.881	0.838	0.857	0.879	0.882
HD_47	0.522	0.533	0.533	0.558	0.569	0.569	0.585	0.587	0.587	0.565	0.584	0.584	0.880	0.837	0.856	0.879	0.882
HD_48	0.527	0.539	0.539	0.564	0.576	0.576	0.589	0.591	0.591	0.571	0.591	0.591	0.881	0.838	0.857	0.879	0.882
**Average**	**0.542**	**0.552**	**0.552**	**0.579**	**0.589**	**0.589**	**0.600**	**0.603**	**0.603**	**0.588**	**0.601**	**0.601**	**0.881**	**0.838**	**0.857**	**0.879**	**0.882**

d) Single nucleotide variant concordance by genotypes identified using Platypus, Samtools, UnifiedGenotyper and Haplotype Caller (single vs. multi sample variant identification) using variants identified with the Illumina BovineHD BeadChip® as a gold standard (BTA1-BTA29)

	**Single sampe variant identification**													**Multi sample variant identification**			
	Platypus			**Platypus (Primitives)**			**Samtools**			**UnifiedGenotyper**			**Haplotype Caller**	**Platypus**	**Platypus (Primitives)**	**Samtools**	**Unified Genotyper**
Animal	***IR+BQSR***	***IR***	***RAW***	**IR+BQSR**	**IR**	**RAW**	**IR+BQSR**	**IR**	**RAW**	**IR+BQSR**	**IR**	**RAW**	**IR+BQSR**	**IR+BQSR**	**IR+BQSR**	**IR+BQSR**	**IR+BQSR**
HD_1	0.997	0.997	0.997	0.989	0.996	0.996	0.997	0.997	0.997	0.996	0.996	0.996	0.995	0.997	0.993	0.984	0.996
HD_2	0.992	0.993	0.993	0.991	0.992	0.992	0.992	0.993	0.993	0.992	0.992	0.992	0.992	0.994	0.993	0.991	0.992
HD_3	0.993	0.993	0.993	0.992	0.992	0.992	0.993	0.993	0.993	0.992	0.993	0.993	0.992	0.994	0.993	0.991	0.993
HD_4	0.992	0.992	0.992	0.991	0.991	0.991	0.992	0.992	0.992	0.992	0.992	0.992	0.991	0.994	0.993	0.990	0.992
HD_5	0.990	0.991	0.991	0.989	0.990	0.990	0.992	0.992	0.992	0.992	0.992	0.992	0.992	0.993	0.993	0.992	0.992
HD_6	0.993	0.993	0.993	0.992	0.992	0.992	0.992	0.992	0.992	0.992	0.992	0.992	0.991	0.994	0.993	0.991	0.992
HD_7	0.981	0.981	0.981	0.979	0.979	0.979	0.979	0.981	0.981	0.979	0.979	0.979	0.982	0.986	0.985	0.980	0.982
HD_8	0.992	0.992	0.992	0.991	0.991	0.991	0.992	0.992	0.992	0.991	0.991	0.991	0.991	0.993	0.993	0.991	0.992
HD_9	0.993	0.993	0.993	0.992	0.992	0.992	0.994	0.994	0.994	0.994	0.994	0.994	0.993	0.995	0.994	0.993	0.994
HD_10	0.994	0.993	0.993	0.993	0.992	0.992	0.994	0.994	0.993	0.994	0.993	0.993	0.993	0.994	0.994	0.994	0.994
HD_11	0.993	0.993	0.993	0.992	0.992	0.993	0.993	0.994	0.994	0.993	0.993	0.993	0.993	0.994	0.994	0.992	0.993
HD_12	0.995	0.996	0.996	0.994	0.995	0.995	0.995	0.996	0.996	0.995	0.995	0.995	0.994	0.995	0.995	0.994	0.995
HD_13	0.994	0.995	0.995	0.993	0.994	0.994	0.994	0.995	0.995	0.994	0.994	0.994	0.993	0.995	0.995	0.993	0.994
HD_14	0.995	0.995	0.995	0.994	0.994	0.994	0.994	0.995	0.995	0.994	0.994	0.994	0.994	0.995	0.995	0.994	0.994
HD_15	0.995	0.996	0.996	0.994	0.995	0.995	0.995	0.995	0.995	0.995	0.995	0.995	0.994	0.995	0.995	0.994	0.995
HD_16	0.993	0.993	0.993	0.992	0.992	0.992	0.993	0.994	0.994	0.993	0.993	0.993	0.993	0.994	0.994	0.993	0.993
HD_17	0.992	0.992	0.992	0.991	0.991	0.991	0.992	0.993	0.993	0.992	0.992	0.992	0.991	0.993	0.993	0.991	0.992
HD_18	0.992	0.992	0.992	0.991	0.991	0.991	0.992	0.992	0.992	0.991	0.991	0.991	0.992	0.993	0.993	0.991	0.992
HD_19	0.992	0.992	0.992	0.991	0.991	0.991	0.991	0.992	0.992	0.991	0.991	0.991	0.992	0.994	0.993	0.991	0.992
HD_20	0.993	0.993	0.993	0.992	0.992	0.992	0.993	0.994	0.994	0.993	0.993	0.993	0.992	0.994	0.994	0.993	0.993
HD_21	0.996	0.995	0.995	0.995	0.994	0.994	0.995	0.995	0.995	0.995	0.995	0.994	0.994	0.995	0.995	0.994	0.995
HD_22	0.995	0.994	0.994	0.994	0.993	0.993	0.995	0.995	0.995	0.994	0.994	0.994	0.993	0.995	0.995	0.994	0.994
HD_23	0.991	0.991	0.991	0.990	0.990	0.990	0.991	0.992	0.992	0.991	0.991	0.991	0.991	0.993	0.993	0.990	0.991
HD_24	0.988	0.988	0.988	0.987	0.987	0.987	0.988	0.989	0.989	0.988	0.988	0.987	0.990	0.991	0.991	0.990	0.990
HD_25	0.995	0.995	0.995	0.994	0.994	0.994	0.995	0.995	0.995	0.995	0.995	0.994	0.994	0.995	0.995	0.994	0.994
HD_26	0.995	0.995	0.995	0.994	0.994	0.994	0.994	0.995	0.995	0.994	0.994	0.994	0.993	0.995	0.995	0.994	0.994
HD_27	0.985	0.986	0.986	0.984	0.984	0.984	0.985	0.986	0.986	0.985	0.985	0.985	0.988	0.990	0.989	0.988	0.988
HD_28	0.995	0.995	0.995	0.994	0.995	0.995	0.995	0.996	0.996	0.995	0.995	0.995	0.994	0.995	0.995	0.994	0.995
HD_29	0.984	0.984	0.984	0.982	0.983	0.983	0.983	0.984	0.985	0.983	0.983	0.983	0.986	0.988	0.988	0.985	0.986
HD_30	0.993	0.993	0.993	0.991	0.992	0.992	0.992	0.993	0.993	0.992	0.993	0.993	0.992	0.994	0.993	0.992	0.993
HD_31	0.994	0.994	0.994	0.993	0.993	0.993	0.993	0.994	0.994	0.993	0.993	0.993	0.993	0.994	0.994	0.993	0.993
HD_32	0.993	0.993	0.993	0.992	0.992	0.992	0.993	0.993	0.993	0.993	0.992	0.992	0.992	0.994	0.994	0.992	0.992
HD_33	0.993	0.993	0.993	0.991	0.991	0.991	0.992	0.992	0.992	0.992	0.992	0.992	0.991	0.993	0.993	0.991	0.992
HD_34	0.986	0.986	0.986	0.985	0.985	0.985	0.986	0.987	0.987	0.986	0.986	0.986	0.988	0.990	0.989	0.988	0.988
HD_35	0.994	0.994	0.994	0.993	0.993	0.993	0.993	0.994	0.994	0.993	0.993	0.993	0.992	0.994	0.994	0.992	0.993
HD_36	0.993	0.994	0.994	0.993	0.993	0.993	0.993	0.994	0.994	0.993	0.993	0.993	0.993	0.994	0.994	0.992	0.993
HD_37	0.991	0.990	0.990	0.990	0.989	0.989	0.991	0.991	0.991	0.991	0.990	0.989	0.989	0.992	0.992	0.990	0.990
HD_38	0.993	0.994	0.994	0.992	0.993	0.993	0.993	0.994	0.994	0.993	0.993	0.993	0.993	0.994	0.994	0.993	0.993
HD_39	0.988	0.989	0.989	0.987	0.987	0.987	0.988	0.989	0.989	0.988	0.988	0.988	0.989	0.991	0.991	0.989	0.990
HD_40	0.993	0.994	0.994	0.992	0.993	0.993	0.993	0.994	0.994	0.993	0.993	0.993	0.992	0.994	0.994	0.993	0.993
HD_41	0.994	0.994	0.994	0.993	0.993	0.993	0.994	0.994	0.994	0.994	0.994	0.994	0.993	0.995	0.994	0.994	0.994
HD_42	0.994	0.994	0.994	0.993	0.993	0.993	0.994	0.995	0.995	0.994	0.994	0.994	0.993	0.995	0.995	0.994	0.994
HD_43	0.993	0.993	0.993	0.992	0.992	0.992	0.993	0.993	0.993	0.992	0.993	0.992	0.992	0.994	0.993	0.991	0.992
HD_44	0.993	0.993	0.993	0.992	0.992	0.992	0.993	0.993	0.993	0.993	0.993	0.992	0.992	0.994	0.994	0.992	0.993
HD_45	0.994	0.994	0.994	0.993	0.993	0.993	0.994	0.994	0.994	0.994	0.993	0.993	0.993	0.994	0.994	0.993	0.994
HD_46	0.992	0.992	0.992	0.991	0.991	0.991	0.992	0.992	0.992	0.992	0.991	0.991	0.991	0.993	0.993	0.991	0.992
HD_47	0.989	0.989	0.989	0.988	0.988	0.988	0.989	0.989	0.989	0.989	0.988	0.988	0.988	0.991	0.990	0.986	0.989
HD_48	0.991	0.992	0.992	0.990	0.991	0.991	0.991	0.992	0.992	0.991	0.991	0.991	0.991	0.993	0.992	0.990	0.991
**Average**	**0.992**	**0.992**	**0.992**	**0.991**	**0.991**	**0.991**	**0.992**	**0.993**	**0.993**	**0.992**	**0.992**	**0.992**	**0.992**	**0.993**	**0.993**	**0.991**	**0.992**

e) Concordance for homozygous reference genotypes identified using Platypus, Samtools, UnifiedGenotyper and Haplotype Caller
(single and multi sample variant identification) using variants identified with the Illumina BovineHD BeadChip® as a gold standard (BTA1-BTA29)

	**Single sampe variant identification**												**Multi sample variant identification**				
	Platypus			**Platypus (Primitives)**			**Samtools**			**UnifiedGenotyper**			**Haplotype Caller**	**Platypus**	**Platypus (Primitives)**	**Samtools**	**Unified Genotyper**
Animal	***IR+BQSR***	***IR***	***RAW***	**IR+BQSR**	**IR**	**RAW**	**IR+BQSR**	**IR**	**RAW**	**IR+BQSR**	**IR**	**RAW**	**IR+BQSR**	**IR+BQSR**	**IR+BQSR**	**IR+BQSR**	**IR+BQSR**
HD_1	0.001	0.002	0.002	0.002	0.003	0.003	0.002	0.002	0.002	0.003	0.003	0.003	0.732	0.702	0.706	0.728	0.734
HD_2	0.001	0.001	0.001	0.003	0.003	0.003	0.003	0.002	0.002	0.003	0.003	0.003	0.713	0.682	0.701	0.709	0.715
HD_3	0.001	0.001	0.001	0.003	0.003	0.003	0.002	0.002	0.002	0.002	0.002	0.003	0.724	0.694	0.712	0.720	0.726
HD_4	0.001	0.001	0.001	0.002	0.002	0.002	0.002	0.002	0.002	0.002	0.002	0.002	0.714	0.683	0.702	0.710	0.716
HD_5	0.001	0.001	0.001	0.002	0.002	0.002	0.002	0.002	0.002	0.002	0.002	0.002	0.713	0.684	0.702	0.709	0.715
HD_6	0.001	0.001	0.001	0.002	0.002	0.002	0.002	0.002	0.002	0.002	0.002	0.002	0.712	0.681	0.700	0.708	0.714
HD_7	0.008	0.009	0.009	0.011	0.011	0.011	0.011	0.009	0.009	0.010	0.011	0.011	0.712	0.680	0.699	0.711	0.715
HD_8	0.001	0.001	0.001	0.002	0.002	0.002	0.002	0.002	0.002	0.002	0.002	0.002	0.719	0.688	0.706	0.713	0.721
HD_9	0.001	0.001	0.001	0.002	0.002	0.002	0.002	0.002	0.002	0.002	0.002	0.002	0.710	0.681	0.699	0.706	0.712
HD_10	0.001	0.001	0.001	0.002	0.002	0.002	0.002	0.002	0.002	0.002	0.002	0.002	0.715	0.687	0.705	0.711	0.717
HD_11	0.001	0.001	0.001	0.002	0.002	0.002	0.002	0.002	0.002	0.002	0.002	0.002	0.719	0.689	0.708	0.714	0.721
HD_12	0.001	0.001	0.001	0.003	0.003	0.003	0.002	0.002	0.002	0.002	0.002	0.002	0.712	0.681	0.700	0.706	0.714
HD_13	0.001	0.001	0.001	0.002	0.003	0.002	0.002	0.002	0.002	0.002	0.002	0.002	0.723	0.692	0.711	0.719	0.725
HD_14	0.001	0.001	0.001	0.002	0.002	0.002	0.002	0.002	0.002	0.002	0.002	0.002	0.724	0.694	0.712	0.719	0.726
HD_15	0.001	0.001	0.001	0.003	0.003	0.003	0.002	0.002	0.002	0.002	0.003	0.003	0.725	0.695	0.714	0.721	0.727
HD_16	0.001	0.001	0.001	0.002	0.002	0.002	0.002	0.002	0.002	0.002	0.002	0.002	0.726	0.696	0.715	0.723	0.728
HD_17	0.001	0.001	0.001	0.003	0.003	0.003	0.002	0.002	0.002	0.002	0.003	0.003	0.720	0.690	0.709	0.716	0.722
HD_18	0.001	0.001	0.001	0.002	0.002	0.002	0.002	0.002	0.002	0.002	0.002	0.002	0.727	0.697	0.716	0.724	0.729
HD_19	0.001	0.001	0.001	0.002	0.002	0.002	0.002	0.002	0.002	0.002	0.002	0.002	0.723	0.693	0.711	0.718	0.725
HD_20	0.001	0.001	0.001	0.003	0.003	0.003	0.002	0.002	0.002	0.003	0.003	0.003	0.717	0.688	0.706	0.713	0.719
HD_21	0.001	0.001	0.001	0.003	0.002	0.002	0.002	0.002	0.002	0.002	0.002	0.002	0.733	0.702	0.721	0.729	0.735
HD_22	0.001	0.001	0.001	0.002	0.002	0.002	0.002	0.002	0.002	0.002	0.002	0.002	0.729	0.699	0.718	0.724	0.731
HD_23	0.001	0.001	0.001	0.003	0.003	0.003	0.002	0.002	0.002	0.003	0.003	0.003	0.720	0.689	0.708	0.716	0.722
HD_24	0.008	0.008	0.008	0.010	0.010	0.010	0.009	0.009	0.009	0.010	0.010	0.010	0.718	0.689	0.708	0.715	0.720
HD_25	0.002	0.002	0.002	0.003	0.003	0.003	0.003	0.003	0.003	0.003	0.003	0.003	0.720	0.691	0.710	0.716	0.722
HD_26	0.001	0.001	0.001	0.003	0.003	0.003	0.002	0.002	0.002	0.002	0.002	0.003	0.714	0.684	0.702	0.709	0.716
HD_27	0.007	0.008	0.008	0.009	0.009	0.009	0.009	0.008	0.008	0.009	0.009	0.010	0.720	0.690	0.709	0.716	0.722
HD_28	0.001	0.001	0.001	0.002	0.002	0.002	0.002	0.002	0.002	0.002	0.002	0.002	0.718	0.689	0.707	0.714	0.720
HD_29	0.009	0.010	0.010	0.012	0.012	0.012	0.012	0.011	0.011	0.012	0.012	0.012	0.705	0.675	0.694	0.701	0.707
HD_30	0.002	0.002	0.002	0.003	0.003	0.003	0.003	0.002	0.002	0.003	0.003	0.003	0.706	0.677	0.695	0.702	0.708
HD_31	0.002	0.002	0.002	0.003	0.003	0.003	0.003	0.002	0.002	0.003	0.003	0.003	0.721	0.692	0.710	0.717	0.723
HD_32	0.001	0.001	0.001	0.003	0.003	0.003	0.003	0.002	0.002	0.003	0.003	0.003	0.706	0.678	0.696	0.703	0.709
HD_33	0.002	0.002	0.002	0.003	0.003	0.003	0.003	0.003	0.003	0.003	0.003	0.003	0.710	0.680	0.699	0.706	0.712
HD_34	0.008	0.008	0.008	0.010	0.010	0.010	0.010	0.009	0.009	0.010	0.010	0.010	0.712	0.681	0.700	0.707	0.714
HD_35	0.001	0.001	0.001	0.002	0.002	0.002	0.002	0.002	0.002	0.002	0.002	0.002	0.721	0.690	0.709	0.717	0.723
HD_36	0.001	0.001	0.001	0.002	0.002	0.002	0.002	0.002	0.002	0.002	0.002	0.002	0.719	0.690	0.708	0.715	0.722
HD_37	0.001	0.001	0.001	0.003	0.003	0.003	0.002	0.002	0.002	0.003	0.003	0.003	0.710	0.679	0.698	0.706	0.712
HD_38	0.001	0.001	0.001	0.002	0.002	0.002	0.002	0.002	0.002	0.002	0.002	0.002	0.708	0.679	0.697	0.704	0.710
HD_39	0.008	0.008	0.008	0.010	0.010	0.010	0.009	0.009	0.009	0.010	0.010	0.010	0.723	0.692	0.711	0.719	0.725
HD_40	0.002	0.002	0.002	0.003	0.003	0.003	0.003	0.003	0.003	0.003	0.003	0.003	0.708	0.679	0.698	0.705	0.710
HD_41	0.001	0.001	0.001	0.002	0.002	0.002	0.002	0.002	0.002	0.002	0.002	0.002	0.712	0.681	0.700	0.707	0.714
HD_42	0.001	0.001	0.001	0.002	0.002	0.002	0.002	0.002	0.002	0.002	0.002	0.002	0.713	0.683	0.701	0.708	0.715
HD_43	0.001	0.001	0.001	0.002	0.002	0.002	0.002	0.002	0.002	0.002	0.002	0.002	0.718	0.687	0.705	0.713	0.720
HD_44	0.001	0.001	0.001	0.002	0.002	0.002	0.002	0.002	0.002	0.002	0.002	0.002	0.713	0.683	0.701	0.709	0.715
HD_45	0.001	0.001	0.001	0.002	0.002	0.002	0.002	0.002	0.002	0.002	0.002	0.002	0.730	0.698	0.717	0.725	0.732
HD_46	0.001	0.001	0.001	0.002	0.002	0.002	0.002	0.002	0.002	0.002	0.002	0.002	0.721	0.691	0.709	0.717	0.723
HD_47	0.001	0.001	0.001	0.003	0.003	0.003	0.003	0.002	0.002	0.003	0.003	0.003	0.718	0.685	0.703	0.714	0.720
HD_48	0.001	0.001	0.001	0.002	0.002	0.002	0.002	0.002	0.002	0.002	0.002	0.002	0.719	0.690	0.708	0.715	0.722
**Average**	**0.002**	**0.002**	**0.002**	**0.003**	**0.004**	**0.003**	**0.003**	**0.003**	**0.003**	**0.003**	**0.003**	**0.003**	**0.718**	**0.687**	**0.706**	**0.713**	**0.720**

f) Concordance for heterozygous genotypes identified using Platypus, Samtools, UnifiedGenotyper and Haplotype Caller
(single and multi sample variant identification) using variants identified with the Illumina BovineHD BeadChip® as a gold standard (BTA1-BTA29)

	**Single sampe variant identification**												**Multi sample variant identification**				
	Platypus			**Platypus (Primitives)**			**Samtools**			**UnifiedGenotyper**			**Haplotype Caller**	**Platypus**	**Platypus (Primitives)**	**Samtools**	**Unified Genotyper**
Animal	***IR+BQSR***	***IR***	***RAW***	**IR+BQSR**	**IR**	**RAW**	**IR+BQSR**	**IR**	**RAW**	**IR+BQSR**	**IR**	**RAW**	**IR+BQSR**	**IR+BQSR**	**IR+BQSR**	**IR+BQSR**	**IR+BQSR**
HD_1	0.886	0.899	0.899	0.870	0.963	0.963	0.958	0.961	0.971	0.959	0.972	0.972	0.980	0.939	0.959	0.980	0.981
HD_2	0.787	0.817	0.817	0.845	0.875	0.875	0.901	0.911	0.911	0.851	0.900	0.900	0.984	0.938	0.960	0.981	0.985
HD_3	0.774	0.811	0.811	0.831	0.868	0.868	0.897	0.910	0.910	0.838	0.896	0.896	0.983	0.938	0.960	0.980	0.983
HD_4	0.776	0.807	0.807	0.834	0.865	0.865	0.894	0.905	0.905	0.843	0.891	0.891	0.983	0.936	0.959	0.981	0.984
HD_5	0.760	0.799	0.799	0.815	0.855	0.855	0.902	0.917	0.917	0.849	0.895	0.895	0.984	0.938	0.960	0.980	0.985
HD_6	0.815	0.843	0.843	0.875	0.902	0.902	0.915	0.925	0.925	0.882	0.920	0.920	0.985	0.936	0.959	0.980	0.985
HD_7	0.737	0.785	0.785	0.787	0.837	0.837	0.859	0.880	0.880	0.794	0.869	0.869	0.984	0.936	0.958	0.978	0.985
HD_8	0.791	0.830	0.830	0.849	0.887	0.887	0.905	0.920	0.920	0.856	0.909	0.909	0.982	0.938	0.959	0.980	0.983
HD_9	0.800	0.831	0.831	0.859	0.890	0.890	0.922	0.934	0.934	0.883	0.919	0.919	0.986	0.938	0.960	0.980	0.986
HD_10	0.844	0.870	0.870	0.906	0.932	0.932	0.944	0.952	0.952	0.922	0.947	0.947	0.985	0.937	0.959	0.980	0.986
HD_11	0.784	0.823	0.823	0.842	0.881	0.881	0.906	0.920	0.920	0.855	0.907	0.907	0.982	0.939	0.960	0.980	0.983
HD_12	0.839	0.866	0.866	0.900	0.928	0.928	0.938	0.948	0.948	0.913	0.943	0.944	0.985	0.938	0.959	0.980	0.986
HD_13	0.812	0.849	0.849	0.870	0.908	0.908	0.921	0.935	0.935	0.884	0.928	0.928	0.983	0.939	0.960	0.980	0.984
HD_14	0.820	0.852	0.852	0.880	0.913	0.913	0.926	0.939	0.939	0.894	0.932	0.932	0.984	0.937	0.959	0.980	0.985
HD_15	0.823	0.855	0.855	0.883	0.915	0.915	0.929	0.941	0.941	0.896	0.933	0.933	0.985	0.938	0.960	0.980	0.986
HD_16	0.787	0.823	0.823	0.845	0.881	0.881	0.908	0.923	0.923	0.862	0.907	0.907	0.985	0.938	0.960	0.980	0.985
HD_17	0.762	0.803	0.803	0.818	0.859	0.859	0.893	0.908	0.908	0.833	0.891	0.891	0.983	0.938	0.960	0.980	0.983
HD_18	0.755	0.796	0.796	0.811	0.852	0.852	0.886	0.901	0.901	0.825	0.886	0.886	0.986	0.937	0.959	0.980	0.986
HD_19	0.766	0.805	0.805	0.822	0.861	0.861	0.891	0.905	0.905	0.836	0.892	0.892	0.983	0.937	0.959	0.981	0.984
HD_20	0.805	0.837	0.837	0.864	0.895	0.895	0.922	0.934	0.934	0.882	0.921	0.921	0.983	0.938	0.960	0.980	0.984
HD_21	0.833	0.849	0.849	0.894	0.909	0.909	0.931	0.942	0.942	0.899	0.931	0.931	0.984	0.938	0.960	0.981	0.985
HD_22	0.839	0.861	0.861	0.900	0.921	0.922	0.942	0.950	0.950	0.918	0.943	0.943	0.982	0.938	0.960	0.980	0.983
HD_23	0.760	0.799	0.799	0.815	0.854	0.854	0.888	0.902	0.902	0.827	0.886	0.886	0.985	0.938	0.960	0.981	0.986
HD_24	0.838	0.859	0.859	0.897	0.918	0.918	0.940	0.950	0.950	0.916	0.940	0.940	0.984	0.938	0.960	0.980	0.984
HD_25	0.839	0.861	0.861	0.899	0.921	0.921	0.942	0.951	0.951	0.918	0.942	0.943	0.984	0.938	0.960	0.981	0.985
HD_26	0.839	0.863	0.863	0.900	0.924	0.924	0.940	0.950	0.950	0.913	0.940	0.941	0.984	0.938	0.959	0.981	0.984
HD_27	0.787	0.819	0.819	0.842	0.875	0.875	0.906	0.920	0.919	0.857	0.902	0.902	0.982	0.937	0.959	0.980	0.983
HD_28	0.830	0.853	0.853	0.890	0.913	0.913	0.937	0.945	0.945	0.909	0.935	0.935	0.983	0.937	0.960	0.981	0.984
HD_29	0.785	0.817	0.817	0.841	0.873	0.873	0.899	0.911	0.911	0.848	0.900	0.900	0.984	0.938	0.960	0.980	0.985
HD_30	0.792	0.828	0.828	0.849	0.886	0.886	0.909	0.922	0.922	0.858	0.911	0.911	0.984	0.939	0.960	0.981	0.985
HD_31	0.797	0.833	0.833	0.856	0.892	0.892	0.913	0.926	0.926	0.866	0.915	0.915	0.984	0.938	0.960	0.981	0.985
HD_32	0.806	0.840	0.840	0.865	0.900	0.900	0.917	0.930	0.930	0.876	0.922	0.922	0.985	0.938	0.960	0.981	0.986
HD_33	0.797	0.836	0.836	0.855	0.894	0.894	0.914	0.927	0.927	0.869	0.917	0.917	0.985	0.939	0.960	0.980	0.985
HD_34	0.800	0.837	0.837	0.856	0.894	0.894	0.913	0.928	0.928	0.870	0.917	0.917	0.983	0.938	0.959	0.980	0.984
HD_35	0.797	0.834	0.834	0.856	0.893	0.893	0.912	0.925	0.925	0.869	0.916	0.916	0.984	0.938	0.959	0.980	0.985
HD_36	0.802	0.835	0.835	0.861	0.895	0.895	0.914	0.925	0.925	0.873	0.917	0.917	0.985	0.938	0.960	0.982	0.986
HD_37	0.792	0.827	0.827	0.849	0.885	0.885	0.910	0.921	0.921	0.861	0.911	0.911	0.984	0.939	0.960	0.981	0.985
HD_38	0.818	0.849	0.849	0.877	0.909	0.909	0.927	0.937	0.937	0.891	0.928	0.928	0.984	0.938	0.959	0.981	0.985
HD_39	0.824	0.852	0.852	0.882	0.910	0.910	0.928	0.940	0.940	0.896	0.930	0.930	0.983	0.939	0.959	0.981	0.984
HD_40	0.798	0.833	0.833	0.856	0.891	0.891	0.917	0.929	0.929	0.874	0.916	0.916	0.985	0.938	0.960	0.981	0.986
HD_41	0.807	0.839	0.839	0.865	0.898	0.898	0.923	0.935	0.935	0.882	0.921	0.921	0.984	0.939	0.960	0.981	0.985
HD_42	0.821	0.848	0.848	0.880	0.907	0.907	0.930	0.940	0.941	0.898	0.929	0.929	0.983	0.937	0.959	0.980	0.984
HD_43	0.789	0.823	0.823	0.845	0.879	0.879	0.907	0.919	0.919	0.860	0.905	0.905	0.984	0.939	0.960	0.980	0.985
HD_44	0.794	0.830	0.830	0.851	0.888	0.887	0.911	0.924	0.924	0.862	0.910	0.910	0.983	0.938	0.959	0.981	0.984
HD_45	0.807	0.839	0.839	0.865	0.897	0.897	0.917	0.931	0.931	0.876	0.918	0.918	0.983	0.939	0.960	0.980	0.983
HD_46	0.794	0.827	0.827	0.852	0.886	0.886	0.909	0.921	0.921	0.863	0.908	0.908	0.983	0.937	0.960	0.980	0.984
HD_47	0.742	0.780	0.780	0.797	0.836	0.836	0.870	0.882	0.882	0.801	0.865	0.865	0.980	0.935	0.958	0.981	0.982
HD_48	0.763	0.803	0.803	0.818	0.858	0.858	0.884	0.898	0.898	0.823	0.889	0.889	0.982	0.937	0.960	0.981	0.983
**Average**	**0.800**	**0.833**	**0.833**	**0.857**	**0.891**	**0.891**	**0.914**	**0.926**	**0.926**	**0.871**	**0.915**	**0.915**	**0.984**	**0.938**	**0.960**	**0.980**	**0.984**

g) Concordance for homozygous alternative genotypes identified using Platypus, Samtools, UnifiedGenotyper and Haplotype Caller
(single and multi sample variant identification) using variants identified with the Illumina BovineHD BeadChip® as a gold standard (BTA1-BTA29)

	**Single sampe variant identification**												**Multi sample variant identification**				
	Platypus			**Platypus (Primitives)**			**Samtools**			**UnifiedGenotyper**			**Haplotype Caller**	**Platypus**	**Platypus (Primitives)**	**Samtools**	**Unified Genotyper**
Animal	***IR+BQSR***	***IR***	***RAW***	**IR+BQSR**	**IR**	**RAW**	**IR+BQSR**	**IR**	**RAW**	**IR+BQSR**	**IR**	**RAW**	**IR+BQSR**	**IR+BQSR**	**IR+BQSR**	**IR+BQSR**	**IR+BQSR**
HD_1	0.886	0.899	0.899	0.870	0.963	0.963	0.958	0.961	0.971	0.959	0.972	0.972	0.980	0.939	0.959	0.980	0.981
HD_2	0.787	0.817	0.817	0.845	0.875	0.875	0.901	0.911	0.911	0.851	0.900	0.900	0.984	0.938	0.960	0.981	0.985
HD_3	0.774	0.811	0.811	0.831	0.868	0.868	0.897	0.910	0.910	0.838	0.896	0.896	0.983	0.938	0.960	0.980	0.983
HD_4	0.776	0.807	0.807	0.834	0.865	0.865	0.894	0.905	0.905	0.843	0.891	0.891	0.983	0.936	0.959	0.981	0.984
HD_5	0.760	0.799	0.799	0.815	0.855	0.855	0.902	0.917	0.917	0.849	0.895	0.895	0.984	0.938	0.960	0.980	0.985
HD_6	0.815	0.843	0.843	0.875	0.902	0.902	0.915	0.925	0.925	0.882	0.920	0.920	0.985	0.936	0.959	0.980	0.985
HD_7	0.737	0.785	0.785	0.787	0.837	0.837	0.859	0.880	0.880	0.794	0.869	0.869	0.984	0.936	0.958	0.978	0.985
HD_8	0.791	0.830	0.830	0.849	0.887	0.887	0.905	0.920	0.920	0.856	0.909	0.909	0.982	0.938	0.959	0.980	0.983
HD_9	0.800	0.831	0.831	0.859	0.890	0.890	0.922	0.934	0.934	0.883	0.919	0.919	0.986	0.938	0.960	0.980	0.986
HD_10	0.844	0.870	0.870	0.906	0.932	0.932	0.944	0.952	0.952	0.922	0.947	0.947	0.985	0.937	0.959	0.980	0.986
HD_11	0.784	0.823	0.823	0.842	0.881	0.881	0.906	0.920	0.920	0.855	0.907	0.907	0.982	0.939	0.960	0.980	0.983
HD_12	0.839	0.866	0.866	0.900	0.928	0.928	0.938	0.948	0.948	0.913	0.943	0.944	0.985	0.938	0.959	0.980	0.986
HD_13	0.812	0.849	0.849	0.870	0.908	0.908	0.921	0.935	0.935	0.884	0.928	0.928	0.983	0.939	0.960	0.980	0.984
HD_14	0.820	0.852	0.852	0.880	0.913	0.913	0.926	0.939	0.939	0.894	0.932	0.932	0.984	0.937	0.959	0.980	0.985
HD_15	0.823	0.855	0.855	0.883	0.915	0.915	0.929	0.941	0.941	0.896	0.933	0.933	0.985	0.938	0.960	0.980	0.986
HD_16	0.787	0.823	0.823	0.845	0.881	0.881	0.908	0.923	0.923	0.862	0.907	0.907	0.985	0.938	0.960	0.980	0.985
HD_17	0.762	0.803	0.803	0.818	0.859	0.859	0.893	0.908	0.908	0.833	0.891	0.891	0.983	0.938	0.960	0.980	0.983
HD_18	0.755	0.796	0.796	0.811	0.852	0.852	0.886	0.901	0.901	0.825	0.886	0.886	0.986	0.937	0.959	0.980	0.986
HD_19	0.766	0.805	0.805	0.822	0.861	0.861	0.891	0.905	0.905	0.836	0.892	0.892	0.983	0.937	0.959	0.981	0.984
HD_20	0.805	0.837	0.837	0.864	0.895	0.895	0.922	0.934	0.934	0.882	0.921	0.921	0.983	0.938	0.960	0.980	0.984
HD_21	0.833	0.849	0.849	0.894	0.909	0.909	0.931	0.942	0.942	0.899	0.931	0.931	0.984	0.938	0.960	0.981	0.985
HD_22	0.839	0.861	0.861	0.900	0.921	0.922	0.942	0.950	0.950	0.918	0.943	0.943	0.982	0.938	0.960	0.980	0.983
HD_23	0.760	0.799	0.799	0.815	0.854	0.854	0.888	0.902	0.902	0.827	0.886	0.886	0.985	0.938	0.960	0.981	0.986
HD_24	0.838	0.859	0.859	0.897	0.918	0.918	0.940	0.950	0.950	0.916	0.940	0.940	0.984	0.938	0.960	0.980	0.984
HD_25	0.839	0.861	0.861	0.899	0.921	0.921	0.942	0.951	0.951	0.918	0.942	0.943	0.984	0.938	0.960	0.981	0.985
HD_26	0.839	0.863	0.863	0.900	0.924	0.924	0.940	0.950	0.950	0.913	0.940	0.941	0.984	0.938	0.959	0.981	0.984
HD_27	0.787	0.819	0.819	0.842	0.875	0.875	0.906	0.920	0.919	0.857	0.902	0.902	0.982	0.937	0.959	0.980	0.983
HD_28	0.830	0.853	0.853	0.890	0.913	0.913	0.937	0.945	0.945	0.909	0.935	0.935	0.983	0.937	0.960	0.981	0.984
HD_29	0.785	0.817	0.817	0.841	0.873	0.873	0.899	0.911	0.911	0.848	0.900	0.900	0.984	0.938	0.960	0.980	0.985
HD_30	0.792	0.828	0.828	0.849	0.886	0.886	0.909	0.922	0.922	0.858	0.911	0.911	0.984	0.939	0.960	0.981	0.985
HD_31	0.797	0.833	0.833	0.856	0.892	0.892	0.913	0.926	0.926	0.866	0.915	0.915	0.984	0.938	0.960	0.981	0.985
HD_32	0.806	0.840	0.840	0.865	0.900	0.900	0.917	0.930	0.930	0.876	0.922	0.922	0.985	0.938	0.960	0.981	0.986
HD_33	0.797	0.836	0.836	0.855	0.894	0.894	0.914	0.927	0.927	0.869	0.917	0.917	0.985	0.939	0.960	0.980	0.985
HD_34	0.800	0.837	0.837	0.856	0.894	0.894	0.913	0.928	0.928	0.870	0.917	0.917	0.983	0.938	0.959	0.980	0.984
HD_35	0.797	0.834	0.834	0.856	0.893	0.893	0.912	0.925	0.925	0.869	0.916	0.916	0.984	0.938	0.959	0.980	0.985
HD_36	0.802	0.835	0.835	0.861	0.895	0.895	0.914	0.925	0.925	0.873	0.917	0.917	0.985	0.938	0.960	0.982	0.986
HD_37	0.792	0.827	0.827	0.849	0.885	0.885	0.910	0.921	0.921	0.861	0.911	0.911	0.984	0.939	0.960	0.981	0.985
HD_38	0.818	0.849	0.849	0.877	0.909	0.909	0.927	0.937	0.937	0.891	0.928	0.928	0.984	0.938	0.959	0.981	0.985
HD_39	0.824	0.852	0.852	0.882	0.910	0.910	0.928	0.940	0.940	0.896	0.930	0.930	0.983	0.939	0.959	0.981	0.984
HD_40	0.798	0.833	0.833	0.856	0.891	0.891	0.917	0.929	0.929	0.874	0.916	0.916	0.985	0.938	0.960	0.981	0.986
HD_41	0.807	0.839	0.839	0.865	0.898	0.898	0.923	0.935	0.935	0.882	0.921	0.921	0.984	0.939	0.960	0.981	0.985
HD_42	0.821	0.848	0.848	0.880	0.907	0.907	0.930	0.940	0.941	0.898	0.929	0.929	0.983	0.937	0.959	0.980	0.984
HD_43	0.789	0.823	0.823	0.845	0.879	0.879	0.907	0.919	0.919	0.860	0.905	0.905	0.984	0.939	0.960	0.980	0.985
HD_44	0.794	0.830	0.830	0.851	0.888	0.887	0.911	0.924	0.924	0.862	0.910	0.910	0.983	0.938	0.959	0.981	0.984
HD_45	0.807	0.839	0.839	0.865	0.897	0.897	0.917	0.931	0.931	0.876	0.918	0.918	0.983	0.939	0.960	0.980	0.983
HD_46	0.794	0.827	0.827	0.852	0.886	0.886	0.909	0.921	0.921	0.863	0.908	0.908	0.983	0.937	0.960	0.980	0.984
HD_47	0.742	0.780	0.780	0.797	0.836	0.836	0.870	0.882	0.882	0.801	0.865	0.865	0.980	0.935	0.958	0.981	0.982
HD_48	0.763	0.803	0.803	0.818	0.858	0.858	0.884	0.898	0.898	0.823	0.889	0.889	0.982	0.937	0.960	0.981	0.983
**Average**	**0.800**	**0.833**	**0.833**	**0.857**	**0.891**	**0.891**	**0.914**	**0.926**	**0.926**	**0.871**	**0.915**	**0.915**	**0.984**	**0.938**	**0.960**	**0.980**	**0.984**

Raw= no InDel realignment or base quality score recalibration
IR= InDel realignment
IR+BQSR= InDel realignment followed by base quality score recalibration
