# Supplementary material for: Evaluation of variant identification methods for whole genome sequencing data in dairy cattle
Source: BMC Genomics. 2014 Nov 1;15(1):948. doi: 10.1186/1471-2164-15-948 (PMC4289218; doi:10.1186/1471-2164-15-948)
Supplement: Supplementary file 5 — Additional file 5: PipelineCode. (PDF 52 KB) [file 12864_2014_6640_MOESM5_ESM.pdf]

Additional File S5: Pipeline commands

Pre-Processing

Indel Realignment:

```
${JAVADIR}java -Xmx3g -jar ${GATK}GenomeAnalysisTK.jar \
-T RealignerTargetCreator \
-R ${reference} \
-I ${temp}${region}.bam \
-known ${dbSNP_work} \
-o ${temp}${region}.RTCintervals.list \
-L ${region} \
-l INFO \
-log ${log}${region}.RealignerTargetCreator.log

${JAVADIR}java -Xmx3g -jar ${GATK}GenomeAnalysisTK.jar \
-T IndelRealigner \
-R ${reference} \
-I ${temp}${region}.bam \
-targetIntervals ${temp}${region}.RTCintervals.list \
-known ${dbSNP_work} \
-L ${region} \
--consensusDeterminationModel USE_SW \
-o ${temp}${region}.realigned.bam \
-l INFO \
-log ${log}${region}.IndelRealigner.log
```

Base Quality Score Recalibration:

```
${JAVADIR}java -Xmx3g -jar ${GATK}GenomeAnalysisTK.jar \
-T BaseRecalibrator \
-R ${reference} \
-L ${region} \
-I ${temp}${region}.realigned.bam \
-knownSites ${dbSNP_work} \
-o ${temp}${region}.realigned.grp \
-l INFO

${JAVADIR}java -Xmx3g -jar ${GATK}GenomeAnalysisTK.jar \
-T BaseRecalibrator \
-R ${reference} \
-L ${region} \
-I ${temp}${region}.realigned.bam \
-BQSR ${temp}${region}.realigned.grp \
-knownSites ${dbSNP_work} \
-o ${temp}${region}.realigned.recal.grp \
-l INFO

${JAVADIR}java -Xmx3g -jar ${GATK}GenomeAnalysisTK.jar \
-T PrintReads \
-R ${reference} \
-L ${region} \
-I ${temp}${region}.realigned.bam \
-BQSR ${temp}${region}.realigned.grp \
-o  ${temp}${region}.realigned.recal.bam \
-l INFO \
-log ${log}${region}.realigned.recal.bam.log
```

Variant Calling

Unified Genotyper:

```
${JAVADIR}java -Xmx3g -jar ${GATK}GenomeAnalysisTK.jar \
-T UnifiedGenotyper \
-R ${reference} \
-L ${region} \
-I ${temp}${region}.bam \
-o ${results}${region}.UG.raw.vcf \
--dbsnp $dbSNP_work \
-out_mode EMIT_VARIANTS_ONLY \
-stand_call_conf 30.0 \
-stand_emit_conf 30.0 \
--genotype_likelihoods_model BOTH \
-l INFO  \
-log ${log}${region}.UnifiedGenotyper.log
```

Haplotype Caller:

```
${JAVADIR}java -Xmx3g -jar ${GATK}GenomeAnalysisTK.jar \
-T HaplotypeCaller \
-R ${reference} \
-L ${region} \
-I ${temp}${region}.bam \
--dbsnp $dbSNP_work \
-stand_call_conf 30.0 \
-stand_emit_conf 30.0 \
-o ${results}${region}.HC.raw.vcf \
-l INFO \
-log ${log}${region}.HaplotypeCaller.log
```

Samtools:

```
${SAMTOOLS}samtools mpileup -f ${reference} -r ${region} -A -C50 -E -D -S -u ${temp}${region}.bam | \
${BCFTOOLS}bcftools view -cvgb - > ${temp}${region}.raw.bcf 2> ${temp}${region}.raw.bcf.log
${BCFTOOLS}bcftools view ${temp}${region}.raw.bcf | ${vcfutils} varFilter -D800 - > ${results}${region}.flt.vcf
```

Platypus:

```
python ${platypus}Platypus.py callVariants --refFile=${reference} --bamFiles=${temp}${region}.bam --output=${results}${region}.platypus.vcf
```
