# Supplementary material for: Evaluation of variant identification methods for whole genome sequencing data in dairy cattle
Source: BMC Genomics. 2014 Nov 1;15(1):948. doi: 10.1186/1471-2164-15-948 (PMC4289218; doi:10.1186/1471-2164-15-948)
Supplement: Supplementary file 6 — Additional file 6: A schematic overview of variant identification pipelines and methods examined in this study. (PDF 241 KB) [file 12864_2014_6640_MOESM6_ESM.pdf]

Additional File S6: A schematic overview of variant identification pipelines and methods examined in this study

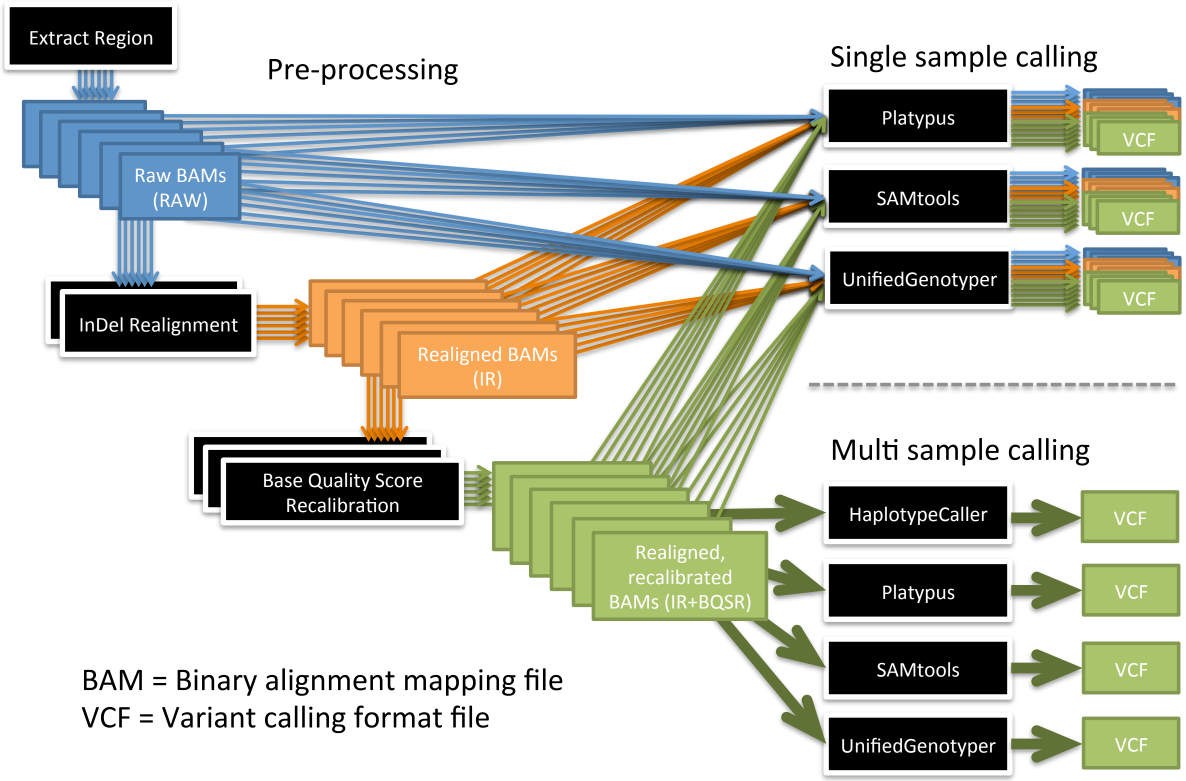

BAM = Binary alignment mapping file
VCF = Variant calling format file
